# Radiosensitising strategies in locally advanced head and neck cancer: a systematic review and network meta-analysis of randomised controlled trials (RADIANCE-NMA)

**DOI:** 10.1016/j.eclinm.2026.104263

**Published:** 2026-10-26

**Authors:** Sreeram MP, Prajwal Dange, Karthik N. Rao, Kevin J. Harrington, Jan B. Vermorken, Arlene A. Forastiere, William M. Mendenhall, Alfio Ferlito, International Head and Neck Scientific Group, Abbas Agaimy, Anna Luíza D. Araújo, Ameya Asarkar, Munita Bal, Jonathan J. Beitler, Carol Bradford, Patrick J. Bradley, Martina Bradová, Eelco de Bree, Remco de Bree, James C.H. Chow, Francisco J. Civantos, Andrés Coca Pelaz, Oded Cohen, Fernando Luiz Dias, Avraham Eisbruch, Veronica Fernández-Alvarez, Gustavo G. Fernandez Ranvier, Renata Ferrarotto, Ewa Florek, Alessandro Franchi, Nina Gale, Oreste Gallo, Eric M. Genden, Paweł Golusinski, Pim de Graaf, Orlando Guntinas-Lichius, Nathan W. Hales, Gyorgy B. Halmos, Marc Hamoir, Ehab Y. Hanna, Dana M. Hartl, Henrik Hellquist, Kevin Higgins, Justin M. Hintze, Akihiro Homma, David Kalfert, Jens Peter Klußmann, Luiz P. Kowalski, Suren Krishnan, Anne W.M. Lee, Reydson A. de Lima Souza, Fernando López, Manish Mair, Fernanda V. Mariano, Jean-Paul Marie, Lauge Hjorth Mikkelsen, Sarina Müller-Hübner, Alfons Nadal, Toshitaka Nagao, Cherie-Ann Nathan, Iain J. Nixon, Wai Tong Ng, Sweet Ping Ng, Ken-ichi Nibu, Sandra Nuyts, Pia Pace-Asciak, Sanjay Pai, Carsten Palme, Cesare Piazza, Miquel Quer, Gregory W. Randolph, Alessandra Rinaldo, K. Thomas Robbins, Juan P. Rodrigo, Ohad Ronen, Nabil F. Saba, Alvaro Sanabria, Ann Sandison, Ashok R. Shaha, Cornelis F.M. Sier, Carl E. Silver, Roderick H.W. Simpson, Elisabeth V. Sjögren, Alena Skálová, Piet J. Slootweg, Robert Smee, Carl H. Snyderman, Maie A. St.John, Göran Stenman, Primož Strojan, Carlos Suárez, Robert P. Takes, Yong Teng, Lester D.R. Thompson, Vincent Vander Poorten, Srivatsa Surya Vasudevan, Isabel Vilaseca, Małgorzata Wierzbicka, Michelle D. Williams, Mark Zafereo, Nina Zidar

**Affiliations:** a Department of Head and Neck Oncology, Sri Shankara Cancer Hospital and Research Centre, Bangalore, India; b Division of Radiotherapy and Imaging, The Institute of Cancer Research and The Royal Marsden NHS Foundation Trust, London, UK; c Faculty of Medicine and Health Sciences, Department of Medical Oncology, Antwerp University Hospital, University of Antwerp, Edegem, Belgium; d Department of Oncology, Johns Hopkins University School of Medicine, Baltimore, MD, USA; e Department of Radiation Oncology, University of Florida College of Medicine, Gainesville, FL, USA; f Coordinator of the International Head and Neck Scientific Group, Padua, Italy

**Keywords:** Head and neck squamous cell carcinoma, Radiosensitiser, Network meta-analysis, Concurrent chemoradiotherapy, Cisplatin, Systematic review

## Abstract

**Background:**

Radiosensitiser enhance radiotherapy efficacy in locally advanced head and neck squamous cell carcinoma (HNSCC). Although cisplatin-based chemoradiotherapy is the standard-of-care, over two dozen radiosensitising strategies have been evaluated in randomised trials with no network meta-analysis comparing all drug classes.

**Methods:**

PubMed, Embase, Cochrane CENTRAL, and ClinicalTrials.gov were searched from inception to 26 June 2026 for Phase IIb–III randomised controlled trials evaluating concurrent radiosensitiser therapy in adults with locally advanced head and neck squamous cell carcinoma (nasopharyngeal carcinoma excluded). Studies lacking overall survival data and induction-only comparisons were excluded. The primary outcome was overall survival (OS). All treatment-effect estimates are reported as hazard ratios (HRs) with 95% confidence intervals (CIs). Data were independently extracted by two reviewers. Risk of bias in individual trials was assessed with the Cochrane Risk of Bias (RoB 2) tool and confidence in the network estimates using the Confidence in Network Meta-Analysis (CINeMA) framework. Random-effects network meta-analysis was done with frequentist (netmeta) and Bayesian (gemtc) frameworks. Between-study heterogeneity was quantified using the I^2^ statistic and τ^2^; publication bias and small-study effects were assessed using comparison-adjusted funnel plots and Egger's regression test. This study is registered with the Open Science Framework (https://osf.io/cygsh).

**Findings:**

A total of 44 trials (13,768 patients; 84% male) were included in the primary analysis, with 23 additional trials in sensitivity analyses (67 total; 19,527 patients). Risk of bias showed some concerns in 39 of 44 (89%) primary trials on the Cochrane RoB 2 tool. Four treatments showed significant overall survival (OS) benefit versus radiotherapy alone: cisplatin plus 5-fluorouracil (hazard ratio (HR) 0.35; 95% confidence interval (CI) 0.18–0.66), nimotuzumab plus weekly cisplatin (0.48; 0.29–0.81), weekly cisplatin (0.57; 0.44–0.74), and three-weekly cisplatin (0.79; 0.65–0.95). In the network estimate, weekly cisplatin was favoured over three-weekly (HR 0.73; 0.58–0.93); the underlying randomised trials were designed as non-inferiority comparisons. Between-study heterogeneity was moderate (I^2^ = 48.9%; τ^2^ = 0.036; Cochran Q = 37.15, df = 19, p = 0.008), with no detected inconsistency (design-by-treatment global test p = 0.88). The comparison-adjusted funnel plot and Egger's regression test showed no evidence of small-study effects, but are interpreted as exploratory given fewer than ten trials per edge on most comparisons. Grade 3–4 toxicity was highest with weekly cisplatin (pooled 71.6% across contributing trials) and lowest with immunotherapy combinations. Meta-regression confirmed enrolment era as a significant effect modifier (β = 0.23 post-2005 versus pre-2005; p = 0.010), consistent with the transition from two-dimensional or conformal radiotherapy in earlier trials to intensity-modulated radiotherapy in modern trials.

**Interpretation:**

Platinum-based multi-agent chemoradiotherapy and weekly cisplatin are the most effective radiosensitising strategies for locally advanced HNSCC. Weekly cisplatin appears at least non-inferior to the widely used three-weekly schedule and may be favoured in network-integrated estimates, though confidence intervals do not exclude clinical equivalence. Neither cetuximab-based regimens (with radiotherapy or added to chemoradiotherapy) nor immune-checkpoint inhibitors added to cisplatin-based chemoradiotherapy significantly improved survival beyond standard cisplatin chemoradiotherapy. These findings provide a unified evidence framework for treatment selection and trial design.

**Funding:**

None.

**Research in context**

**Evidence before this study**

PubMed, Embase, Cochrane CENTRAL, and ClinicalTrials.gov were searched from inception to 26 June 2026, using search terms combining head and neck neoplasms, radiotherapy, radiosensitisers, chemotherapy, and randomised controlled trials, with no language restrictions. Eligible studies were Phase IIb–III randomised controlled trials of concurrent radiosensitiser therapy in adults with locally advanced head and neck squamous cell carcinoma reporting overall survival. Studies were excluded if they lacked overall survival data, compared only induction regimens, or enrolled fewer than 20 patients per arm. The Meta-Analysis of Chemotherapy in Head and Neck Cancer (MACH-NC) individual patient data meta-analysis (107 trials, 19,805 patients) established that concurrent chemoradiotherapy improves overall survival (HR 0.83) compared with radiotherapy alone, but compared only broad timing categories without distinguishing specific agents or schedules. The Meta-Analysis of Radiotherapy in Carcinomas of Head and neck (MARCH) meta-analysis quantified altered-fractionation benefits. Risk of bias in existing evidence was variable, with most trials at some concern on the Cochrane RoB 2 tool. Existing network meta-analyses focused predominantly on chemotherapy and cetuximab, without incorporating hypoxic sensitisers, nanoparticles, or immunotherapy combinations. No prior study simultaneously compared all major radiosensitiser drug classes within a unified network framework.

**Added value of this study**

This network meta-analysis brings together 44 randomised controlled trials (13,768 patients) across 24 treatment nodes to compare concurrent radiosensitising strategies for locally advanced head and neck squamous cell carcinoma within a single analytical framework, spanning platinum agents, fluoropyrimidines, epidermal growth factor receptor (EGFR) inhibitors, hypoxic cell sensitisers, immunotherapy combinations, and novel molecular agents. It provides a hierarchical ranking of these strategies alongside their toxicity profiles, and integrates direct and indirect evidence not available from pairwise meta-analyses.

**Implications of all the available evidence**

For practice, cisplatin-based chemoradiotherapy remains the preferred option for adults with locally advanced HNSCC who can tolerate platinum therapy; weekly cisplatin may be considered an acceptable alternative to three-weekly cisplatin, recognising that the underlying randomised trials were non-inferiority or exploratory designs. Cetuximab-based chemoradiotherapy has not demonstrated equivalent overall-survival outcomes to cisplatin-based chemoradiotherapy in the Phase III trials to date and is best reserved for those who are cisplatin-ineligible. For policy, treatment recommendations should be interpreted in the context of contemporary radiotherapy standards, given the attenuation of chemotherapy benefit observed in the modern radiotherapy era. For future research, priorities include individual-participant-data network meta-analyses to enable human papillomavirus (HPV)-stratified recommendations, adequately powered trials of concurrent immune-checkpoint inhibition, low-dose or attenuated-frequency immunotherapy schedules, sequencing strategies, and emerging nanoparticle and DNA-damage-response agents.

## Introduction

Head and neck squamous cell carcinoma (HNSCC) is the sixth most common malignancy globally, with approximately 467,000 deaths annually. Despite advances in multimodal treatment, the 5-year survival rate for locally-advanced disease remains approximately 40–50%. Radiotherapy plays a pivotal role in HNSCC management, but its efficacy is limited by inherent radioresistance driven by tumour hypoxia, DNA repair mechanisms, and cell cycle checkpoint activation.^1^^,^^2^

Radiosensitiser enhance tumour cell death when combined with radiation without proportionally increasing normal tissue damage.^3^^,^^4^ The concept has evolved from conventional chemotherapeutic agents to targeted inhibitors and nanoparticle platforms.^5^^,^^6^ Cisplatin-based chemoradiotherapy has remained the standard-of-care since landmark trials demonstrated improved survival,^7^^,^^8^ but is associated with substantial toxicities that limit tolerability in patients who are elderly or frail.

The Meta-Analysis of Chemotherapy in Head and Neck Cancer (MACH-NC) established the overall survival benefit of concurrent chemoradiotherapy (hazard ratio (HR) 0.83) but compared only broad treatment categories.^7^^,^^8^ The Meta-Analysis of Radiotherapy in Carcinomas of Head and Neck (MARCH) meta-analysis quantified altered fractionation benefits but not molecular radiosensitiser.^9^^,^^10^ Prior network meta-analysis (NMA) work focused on chemotherapy and cetuximab without incorporating hypoxic sensitisers, nanoparticles, or immunotherapy,^11^ and a recent non-cisplatin NMA was restricted to patients who are cisplatin-ineligible.^12^ Pairwise meta-analyses cannot simultaneously rank multiple treatments.^13^

Recent Phase III trials in patients with human papillomavirus (HPV)-positive disease have reshaped the landscape: the De-ESCALaTE HPV trial, the Radiation Therapy Oncology Group (RTOG) 1016 trial, and the Trans-Tasman Radiation Oncology Group (TROG) 12.01 trial demonstrated cetuximab inferiority to cisplatin,^14^, ^15^, ^16^ while the Japan Clinical Oncology Group (JCOG) 1008 trial established weekly cisplatin non-inferiority.^17^ Concurrent immunotherapy with pembrolizumab (KEYNOTE-412),^18^ avelumab,^19^ and durvalumab (COMPARE)^20^ delivered negative results. Xevinapant (TrilynX)^21^ disappointed despite promising Phase II data. Perioperative pembrolizumab (KEYNOTE-689)^22^ and adjuvant nivolumab (NIVOPOSTOP)^23^ showed benefit without mature overall survival (OS) data. This evidence demands an integrative framework.

Against this background, we conducted a systematic review and network meta-analysis of concurrent radiosensitising strategies in adults with locally advanced HNSCC. Our primary research question was whether, and to what extent, different mechanistic classes of concurrent radiosensitiser (conventional chemotherapeutic agents, epidermal growth factor receptor (EGFR)-targeted antibodies, hypoxic cell sensitisers, immune-checkpoint inhibitors, and novel molecular agents) modify overall survival relative to radiotherapy alone and to one another. Secondary questions concerned progression-free and locoregional-recurrence-free survival, treatment-related toxicity, and the modification of comparative effects by trial-level, tumour-level, and patient-level characteristics. The systematic review was conducted in accordance with the Preferred Reporting Items for Systematic Reviews and Meta-Analyses extension for network meta-analyses (PRISMA-NMA); risk of bias for individual trials was assessed with the Cochrane Risk-of-Bias tool for randomised trials (RoB 2); and the certainty of pooled network estimates was rated using the Confidence in Network Meta-Analysis (CINeMA) framework.

## Methods

This systematic review and NMA followed the Preferred Reporting Items for Systematic Reviews and Meta-Analyses (PRISMA) 2020 guidelines^1^ and NMA extension.^24^ The study protocol was developed a priori and followed established methodological frameworks for network meta-analysis.^25^ The primary outcome was overall survival, and the primary effect measure was the hazard ratio with 95% confidence intervals. This study is registered with the Open Science Framework (OSF; https://osf.io/cygsh). An artificial intelligence (AI)-assisted technology tool (Claude, Anthropic; claude-sonnet-4-20250514) was used for manuscript formatting. All AI-generated outputs were critically reviewed and verified by the authors. The research question followed a Population, Intervention, Comparator, Outcome (PICO) framework—Population: adults with locally advanced HNSCC; Intervention: any concurrent radiosensitiser given with radiotherapy; Comparator: any other radiosensitiser or radiotherapy alone; Outcome: overall survival. Sex was reported at the study level (proportion male/female) where available; gender identity was not consistently reported across included trials, in line with the historic vintage of many studies (Sex and Gender Equity in Research (SAGER) guidance limitations). The full Supplementary Appendix accompanying this manuscript comprises: Search Strategy; Supplementary Figures S1 (RoB traffic-light), S1a (RoB per-domain summary), S1b (RoB weighted summary bar plot), S2 (full sensitivity network), S3 (net heat plot for inconsistency), S4 (sensitivity-analysis forest), S5–S6 (setting subgroup forests), S7 (comparison-adjusted funnel), S8 (Grade 3–4 toxicity heatmap), S9 (efficacy–toxicity scatter), S10 (direct-evidence forest plots), S11 (node-splitting), S12 (pre-specified subgroup forest), S13 (subsite meta-regression bubble), S14 (era meta-regression bubble), S15 (era-stratified forest), and S16 (weekly versus three-weekly cisplatin direct-evidence forest); and Supplementary Tables S1 (exclusion codes and reasons), S2 (RoB per-study per-domain), S3 (treatment-node definitions), S4 (P-scores versus SUCRA concordance), S5 (setting and sensitivity subgroup NMA HRs), S6 (full pairwise league table), S7 (excluded studies with codes), S8 (node-splitting per comparison), S9 (per-edge heterogeneity), S10 (Grade 3–4 toxicity per treatment), S11 (treatment-related deaths per drug class), S12 (meta-regression covariate table), and S13 (era-stratified NMA—P-scores and rankings).

### Search strategy and selection criteria

PubMed/MEDLINE, Embase, and Cochrane CENTRAL were searched from inception to 26 June 2026 with no language restrictions. ClinicalTrials.gov was searched for completed trials. Search terms combined controlled vocabulary and free-text terms across three domains: head and neck cancer, radiosensitising agents, and randomised trial design. The complete search strategy is provided in the Supplementary Material. Backward reference checking (“snowballing”) of all included studies' reference lists yielded 6 additional studies not identified through database searching. Conference abstracts and grey literature were not systematically searched. No unpublished data were sought from study authors.

Studies were eligible if they met all of the following criteria: (1) randomised controlled trial design (Phase IIb or III; Phase IIa single-arm screening designs excluded); (2) at least 20 patients per arm (threshold chosen to ensure stable hazard ratio estimation and minimum precision for inclusion in network meta-analysis) with histologically-confirmed, locally-advanced HNSCC of the oral cavity, oropharynx, hypopharynx, or larynx (Stage III-IVB; nasopharyngeal carcinoma excluded); (3) at least one arm including a concurrent radiosensitiser with external beam radiotherapy (RT); (4) OS hazard ratio reported or estimable; and (5) at least one arm mappable to an existing network node. Studies without a radiotherapy-alone control arm were eligible provided both treatment arms could be mapped to existing network nodes (e.g., weekly versus three-weekly cisplatin schedules).

Studies were excluded for the following: no OS data; non-estimable hazard ratio; induction-only comparisons without concurrent radiosensitiser difference; treatment modality comparisons (radiotherapy (RT) versus surgery); safety reports without efficacy data; single-arm Phase IIa designs; subgroup analyses of included parent trials (to avoid double-counting; eligible biomarker-stratified subgroup data published separately could be used in subgroup or sensitivity analyses); or de-escalation trials with non-standard radiotherapy doses; and trials in which both arms received accelerated or altered fractionation as the backbone (such studies were retained in sensitivity analyses where node connectivity permitted). Complete exclusion criteria are provided in Supplementary Table S1. Screening was performed independently by two reviewers, PD (Prajwal Dange) and SMP (Sreeram MP), with disagreements resolved by a third reviewer (KNR).

### Data extraction

Data were extracted independently by two reviewers (PD, SMP; conflicts resolved by KNR) covering study characteristics, demographics, treatment details, outcomes). Where HRs were not directly reported (∼36% of studies, 15/42 OS-reporting trials in the primary network), indirect methods were used: the Peto log-rank approach or the Tierney method.^26^ All HRs were log-transformed and harmonised (HR < 1 favours experimental treatment). For multi-arm trials, only pairwise comparisons relevant to network structure were retained: Forastiere et al.^27^ retained concurrent cisplatin versus RT alone only; Adelstein et al.^28^ excluded the split-course arm. Dietz et al.^29^ was moved to sensitivity analysis due to its treatment nodes lacking connectivity to the primary network.^29^ For each study, the reported (or derived) hazard ratio was treated as a time-invariant summary under the proportional-hazards assumption, consistent with standard NMA practice; we did not undertake landmark (2-year or 5-year) pooling. Race, ethnicity, and disability status were not collected as part of this systematic review beyond what individual trials had reported. Race and ethnicity were inconsistently recorded across the included trials, and the majority of the older randomised trials (particularly those enrolled before the mid-2000s) did not report race or ethnicity at all; where reported, categories followed the original study definitions, and we did not attempt to harmonise or re-categorise them because the original definitions, conceptual frameworks (self-identified versus investigator-assigned), and geographic contexts differed substantially between trials. Disability status was likewise not systematically reported by the included trials; where performance-status measures (Karnofsky Performance Scale or Eastern Cooperative Oncology Group score) were reported, these captured functional performance rather than validated disability measures. We therefore did not undertake any stratified analyses by race, ethnicity, or disability. This limitation is acknowledged in the Discussion.

### Treatment node classification

Treatment nodes were defined by pharmacological mechanism, dosing schedule, and route of administration. The primary network comprises 24 nodes connected by 27 unique direct treatment-pair comparisons across 42 trials reporting overall survival (44 trials in the broader main network when including 2 trials without OS data—Overgaard 1998^30^ and Rischin 2021^16^). Studies comparing the same drug via different routes or schedules were classified as distinct nodes based on differing radiosensitising interactions (Supplementary Table S3 details all treatment nodes and their constituent regimens).

### Quality assessment

Risk of bias was assessed using the Cochrane RoB 2 tool^31^ (Supplementary Figure S1, Supplementary Table S2). Confidence in NMA estimates was evaluated using the CINeMA framework.^32^

### Statistical analysis

The primary analysis used a frequentist random-effects network meta-analysis via the graph-theoretical approach^25^ implemented in the netmeta package,^33^ with radiotherapy alone as the reference node. Treatment rankings were derived using P-scores,^34^ which extend the frequentist surface under the cumulative ranking curve (SUCRA) conceptwithout resampling. All reported hazard ratios are pooled random-effects estimates on the log-hazard-ratio scale with 95% confidence intervals; prediction intervals were reported alongside confidence intervals to convey the range of true underlying treatment effects expected in a new study.

### Bayesian analysis

A Bayesian random-effects network meta-analysis was performed in parallel with the frequentist analysis as a pre-specified robustness check, using the gemtc R package (version 1·0-2) with rjags as the Markov chain Monte Carlo (MCMC) sampler. The rationale for the parallel Bayesian analysis was threefold: (i) to derive full posterior distributions for treatment rankings and SUCRA values,^35^ which are natively probabilistic; (ii) to confirm treatment-hierarchy stability across two conceptually distinct estimation frameworks; and (iii) to allow explicit propagation of parameter uncertainty into ranking statements. A consistency model was fitted with a random-effects structure, a Normal likelihood, and an identity link on the log-hazard-ratio scale. Vague (weakly informative) priors as implemented by default in gemtc were used to allow the observed trial data to dominate the posterior: a very wide Normal prior (mean 0, precision approaching 0) was placed on each log hazard ratio for treatment effects, and a Uniform(0, ω) prior was placed on the between-study heterogeneity standard deviation τ, where ω is auto-scaled by gemtc from the empirical range of observed effect sizes (approximately 15 × the maximum reported standard error). Four MCMC chains were run with 5000 adaptation/burn-in iterations followed by 50,000 sampling iterations per chain, with a thinning interval of 10. Convergence was assessed by trace-plot inspection and the Gelman–Rubin diagnostic; all treatment-effect potential scale reduction factors were <1.05, indicating satisfactory convergence. Concordance between frequentist P-scores and Bayesian SUCRA values was quantified as the mean absolute difference in percentage ranks.

Consistency between direct and indirect evidence was evaluated globally using the design-by-treatment interaction model and locally using node-splitting analysis^36^; the net heat plot was used to visualise design contributions and any local inconsistency.^37^ Between-study heterogeneity was quantified using the I^2^ statistic,^38^ τ^2^ (between-study variance), and Cochran Q. Risk of bias in individual trials was assessed with the Cochrane RoB 2 tool,^31^ and confidence in the network estimates was rated using the CINeMA framework.^32^ Publication bias and small-study effects were assessed using comparison-adjusted funnel plots and Egger's regression test; because most treatment-pair edges contained fewer than ten trials, these small-study-effect analyses are labelled as exploratory and interpreted with caution.

Detailed study characteristics for the primary NMA are summarised in Table 1 (see Table 2 for sensitivity-network studies; the complete list of excluded studies with codes is provided in Supplementary Table S7). Pre-specified sensitivity analyses included: (1) full 67-study network (44 primary + 23 sensitivity studies); (2) exclusion of an extreme outlier (Rodriguez et al. 2015,^85^ HR 5.3 from 9 deaths); (3) exclusion of two asymmetric induction studies (Merlano et al. 2020^88^ and Geoffrois et al. 2018,^86^ where only the experimental arm received TPF (docetaxel, cisplatin, and 5-fluorouracil) induction); and(4) restriction to definitive-setting studies only (definitive = primary, curative-intent chemoradiotherapy without upfront surgery, as opposed to postoperative adjuvant chemoradiotherapy). Subgroup analyses by treatment setting and meta-regression of oropharyngeal proportion and enrolment year were also performed. One trial (NIMRAD-2; Grégoire et al.^93^) could not be integrated into the network because its accelerated-fractionation backbone with non-randomised cisplatin scheduling did not map to any standard treatment node. All subgroup, sensitivity, and meta-regression analyses reported here were pre-specified in the study protocol; no post-hoc analyses were undertaken during peer review Full trial-level characteristics of the 23 sensitivity-analysis studies are provided in Table 2.

**Table 1:** Characteristics of the 42 OS-reporting randomised controlled trials included in the primary network meta-analysis (the broader main NMA includes 2 additional trials with only LRC or FFS endpoints: Overgaard 1998 and Rischin 2021, for a total of 44 primary-NMA trials).

Study	Trial name	Country	Phase	Setting	NOSScore	N(Total)	Control Arm	n(Ctrl)	Experimental Arm	n(Exp)	RT(Gy)	F/U(mo)	HR (95% CI)	p-value	Male %	Female %	HPVStatus	Funding
Henk et al. (1977)^39^	Cardiff HBO Trial 2	United Kingdom	III	Definitive	3/8	103	RT ALONE	52	HBO RT	51	64	NR	0.69 (0.40–1.20)	0.020	NR	NR	NR	Government
Fazekas et al. (1987) ^40^	RTOG 79-15	USA	III	Definitive	3/8	297	RT ALONE	150	MISO RT	147	NR	NR	1.27 (0.71–2.27)	NR	NR	NR	NR	Government
Browman et al. (1994)^41^	Ontario I-FU Trial	Canada	III	Definitive	6/8	175	RT ALONE	87	5FU RT	88	66	42	0.73 (0.46–1.15)	0.076	78.2	21.8	NR	Government
Lee et al. (1995)^42^	RTOG 85-27	United States	III	Definitive	6/8	504	RT ALONE	252	ETANIDAZOLE RT	252	66–74	40.6	0.95 (0.77–1.17)	0.650	72	28	NR	Government
Bachaud et al. (1996)^43^	French Adjuvant Cisplatin Trial	France	III	Adjuvant	4/8	88	RT ALONE	45	CIS WK	43	65–74	60	0.26 (0.10–0.71)	0.010	NR	NR	NR	NR
Eschwege et al. (1997)^44^	European Etanidazole Trial	Europe (Multicenter)	III	Definitive	4/8	374	RT ALONE	187	ETANIDAZOLE RT	187	NR	60	1.05 (0.68–1.61)	NR	NR	NR	NR	Mixed
Overgaard et al. (1998)^30^	DAHANCA 5-85	Denmark	III	Definitive	8/8	414	RT ALONE	195	NIMORAZOLE RT	219	NR	126	0.69 (0.52–0.90)^a^	NR	NR	NR	NR	Government
Wendt et al. (1998)^45^	German Multicenter RCT Study	Germany	III	Definitive	4/8	298	RT ALONE	145	CIS 5FU RT	153	70.2	NR	0.35 (0.21–0.57)	<0.001	90	10	NR	NR
Calais et al. (1999)^46^	GORTEC 94-01	France	III	Definitive	5/8	222	RT ALONE	113	CARBO 5FU RT	109	70	66	0.75 (0.56–1.00)	0.050	89	11	NR	Government
Adelstein et al. (2003)^28^	E1392 (Head and Neck Intergroup)	United States	III	Definitive	5/8	182	RT ALONE	95	CIS 3W	87	70	41	0.68 (0.48–0.96)	0.028	90	10	NR	Government
Henke et al. (2003)^47^	ENHANCE	Multicenter (Austria/France/Germany/Switzerland)	III	Mixed (Definitive + Postoperative)	6/8	351	RT ALONE	171	EPO RT	180	60–70	NR	1.39 (1.05–1.84)	0.020	85	15	NR	Industry
Bernier et al. (2004)^48^	EORTC 22931	International (Europe)	III	Adjuvant	6/8	334	RT ALONE	167	CIS 3W	167	66	60	0.70 (0.52–0.95)	0.020	92	8	NR	Government
Hoskin et al. (2009)^49^	UK EPO RT Trial	United Kingdom	III	Mixed (Definitive + Postoperative)	6/8	300	RT ALONE	149	EPO RT	151	60–70	60	1.04 (0.75–1.43)	0.823	79	21	NR	Industry
Bonner et al. (2010)^50^	IMCL-9815	United States and 14 other countries	III	Definitive	5/8	424	RT ALONE	213	CETUX RT	211	70–76.8	NR	0.73 (0.56–0.95)	0.018	NR	NR	NR	Industry
Cooper et al. (2012)^51^	RTOG 9501	USA	III	Adjuvant	6/8	410	RT ALONE	208	CIS 3W	202	60	112.8	0.89 (0.70–1.12)	202.000	NR	NR	NR	Government
Ghadjar et al. (2012)^52^	Swiss HN Trial	Switzerland	III	Definitive	5/8	224	RT ALONE	112	CIS DAILY	112	NR	NR	0.77 (0.56–1.11)	NR	NR	NR	NR	Government
Forastiere et al. (2013)^27^	RTOG 91-11	United States	III	Definitive	6/8	367	CIS 3W	182	RT ALONE	185	70	129.6	1.08 (0.85–1.39)	0.080	NR	NR	NR	Government
Giralt et al. (2015)^53^	CONCERT-2	International (8 countries)	II	Definitive	5/8	152	CIS 3W	62	PANI RT	90	70–72	28.4	1.59 (0.91–2.79)	0.100	90	10	17%	Amgen
Ghosh-Laskar et al. (2016)^54^	NR	India	III	Definitive	4/8	131	RT ALONE	62	CIS WK	69	70	96	0.48 (0.24–0.97)	0.040	93	7	NR	Government
Heukelom et al. (2016)^55^	RADPLAT	Netherlands/New Zealand	III	Definitive	5/8	237	CIS 3W	119	IA CIS RT	118	70	90	0.85 (0.62–1.17)	0.316	83	17	NR	Government
Yi et al. (2017)^56^	NR	China	III	Definitive	5/8	222	RT ALONE	118	CIS WK	104	50	59	0.74 (0.50–1.10)	0.130	82.2	17.8	NR	Government
Gillison et al. (2019)^15^	NRG Oncology RTOG 1016	United States and Canada	III	Definitive	7/8	805	CETUX RT	399	CIS 3W	406	70	54	1.45 (1.03–2.05)	0.016	89	11	100%	Mixed
Mehanna et al. (2019)^14^	De-ESCALaTE HPV	UK Ireland Netherlands	III	Definitive	6/8	334	CIS 3W	166	CETUX RT	168	70	25.9	5.00 (1.70–14.70)	0.001	80	20	94%	Government
Patil et al. (2019)^57^	TMC Nimotuzumab Phase III	India	III	Definitive	6/8	536	CIS WK	268	NIMO CIS WK	268	66–70	39.13	0.84 (0.65–1.08)	0.163	86.2	13.8	8%	Mixed
Fietkau et al. (2020)^58^	PacCis	Germany	II	Definitive	4/8	216	PAC CIS RT	105	CIS 5FU RT	111	63.6	33.9	0.82 (0.54–1.24)	0.350	80	20	NR	German Research Foundation
Maddalo et al. (2020)^59^	NCT01216020	Italy	III	Definitive	4/8	70	CIS WK	35	CETUX RT	35	NR	64.8	1.41 (0.55–3.59)	NR	NR	NR	Mixed	Government
Wozniak et al. (2020)^60^	NR	Poland	III	Adjuvant	5/8	111	RT ALONE	57	CIS 3W	54	63	64.8	1.03 (0.57–1.87)	0.900	68	32	NR	Government
Lee et al. (2021)^19^	JAVELIN HN 100	International	III	Definitive	7/8	697	CIS 3W	347	AVELUMAB CIS 3W	350	NR	29	1.31 (0.93–1.85)	NR	NR	NR	Mixed	Industry
Rischin et al. (2021)^16^	TROG 12.01	Australia/New Zealand	III	Definitive	7/8	189	CIS WK	96	CETUX RT	93	70	49.2	3.00 (1.20–7.70)^a^	0.015	90	10	100%	Mixed
Hitt et al. (2022)^61^	TTCC-2007-01	Spain	III	Definitive	6/8	407	CIS 3W	205	CETUX RT	202	NR	67	1.11 (0.85–1.44)	NR	NR	NR	Mixed (majority p16-)	Government
Kiyota et al. (2022)^17^	JCOG1008	Japan	II/III	Adjuvant	5/8	261	CIS 3W	132	CIS WK	129	NR	50	0.75 (0.50–1.13)	NR	NR	NR	NR	Government
Tao et al. (2022)^62^	GORTEC 2015–01 PembroRad	France and Switzerland	II	Definitive	5/8	133	CETUX RT	66	IMMUNO CRT	67	69.96	25	0.83 (0.49–1.40)	0.490	82	18	27%	Industry
Caudell et al. (2023)^63^	NRG/RTOG 0522	USA/Canada	III	Definitive	6/8	891	CIS 3W	447	CIS 3W CETUX	444	70	121	0.97 (0.80–1.16)	0.360	NR	NR	Mixed (235 p16+ OPC)	NCI/Eli Lilly
Patil et al. (2023)^64^	TMC DOC-RT	India	III	Mixed	5/8	356	RT ALONE	176	DOC WK RT	180	NR	42	0.75 (0.57–0.98)	NR	NR	NR	NR	Government
Tao et al. (2023)^65^	NCT02022098 Phase 2 (Debio 1143)	France, Switzerland	II	Definitive	7/8	96	CIS 3W	48	XEVINAPANT CRT	48	70	60.1	0.47 (0.27–0.84)	0.010	NR	NR	6%	Industry
Zhang et al. (2023)^66^	NR	China	II	Both	4/8	128	CIS 3W	62	LOBAPLATIN 3W	66	69.96	42	0.88 (0.40–1.94)	0.841	91	9	NR	Government
Machiels et al. (2024)^18^	KEYNOTE-412	Global (130 centres)	III	Definitive	8/8	804	CIS 3W	402	PEMBRO CRT	402	70	47.7	0.90 (0.71–1.15)	NR	82	18	27%	Industry
Thomson et al. (2024)^67^	NIMRAD	United Kingdom	III	Definitive	8/8	338	RT ALONE	170	NIMORAZ	168	65	37.2	0.92 (0.65–1.31)	0.880	75.9	24.1	45%	Academic
Bourhis et al. (2025)^23^	TrilynX	International (26 countries)	III	Definitive	7/8	730	CIS 3W	366	XEVINAPANT CIS 3W	364	70	18	1.39 (1.04–1.86)	0.993	NR	NR	0%	Industry
Gebre-Medhin et al. (2025)^68^	ARTSCAN III	Sweden	III	Definitive	7/8	298	CIS WK	149	CETUX RT	149	68.0	90	1.66 (1.08–2.56)	0.021	80	20	90%	Swedish Cancer Society
Noronha et al. (2025)^69^	Tata Memorial Weekly versus 3-Weekly Cisplatin	India	III	Adjuvant (93%) Definitive (7%)	5/8	300	CIS WK	150	CIS 3W	150	60–70	82.9	1.21 (0.90–1.62)	0.190	90	10	NR	Institution
Nair et al. (2017)^70^	Trivandrum Cisplatin Schedule	India	IIb	Definitive	3/8	55	CIS 3W	31	CIS WK	24	66	26	1.27 (0.51–3.14)	0.61	NR	NR	NR	Institution
Jiang et al. (2024)^71^	Shanghai Adjuvant Docetaxel versus Cisplatin	China	IIb	Adjuvant	6/8	224	CIS 3W	112	DOC WK RT	112	60–66	28.8	0.87 (0.55–1.38)	0.56	NR	NR	NR	Institution
Nanda et al. (2019)^72^	NR	India	IIb	Definitive	3/8	60	CIS 3W	31	CIS WK	29	70	28	0.69 (0.34–1.40)	0.303	93.1	6.9	NR	None

NOS, Newcastle-Ottawa Scale (adapted for RCTs; maximum 8 stars: Selection 0–3 [randomisation, multicenter, adequate N], Comparability 0–2 [blinding, HPV reporting], Outcome 0–3 [follow-up >36mo, objective OS endpoint, HR directly reported]); RCT, randomised controlled trial; N (Total), total number of participants; n (Ctrl), number in control arm; n (Exp), number in experimental arm; Male %, proportion of male participants at study level (per Sex and Gender Equity in Research (SAGER) guidance); Female %, proportion of female participants at study level; p-value, two-sided p-value for the primary treatment comparison; F/U, follow-up; mo, months; HR, hazard ratio; CI, confidence interval; OS, overall survival; LRC, locoregional control; FFS, failure-free survival; RT, radiotherapy; Gy, Grey; NR, not reported; Ctrl, control arm; Exp, experimental arm; HPV, human papillomavirus. Drug and schedule abbreviations: CIS, cisplatin; WK, weekly; 3W, three-weekly; 5FU, 5-fluorouracil; CARBO, carboplatin; CETUX, cetuximab; NIMO, nimotuzumab; NIMORAZ, nimorazole; PANI, panitumumab; DURVA, durvalumab; NIVO, nivolumab; PEMBRO, pembrolizumab; TPF, docetaxel plus cisplatin plus 5-fluorouracil induction; HBO, hyperbaric oxygen. Fractionation and delivery abbreviations: ACCEL, accelerated fractionation; AFX, altered fractionation; CBRT, concomitant boost radiotherapy; CRT, chemoradiotherapy. HR < 1 favours experimental arm; HR > 1 favours control arm.

a Denotes non-OS endpoint: Overgaard 1998 reports LRC HR; Rischin 2021 reports FFS HR (OS not reported).

**Table 2:** Characteristics of the 23 sensitivity-only studies included in the expanded network meta-analysis.^a^

Study	Trial name	Country	Phase	Setting	NOSScore	N(Total)	Control Arm	n(Ctrl)	Experimental Arm	n(Exp)	RT(Gy)	F/U(mo)	HR (95% CI)	p-value	Male %	Female %	HPVStatus	Funding
Bourhis et al. (2011)^73^	GORTEC Dose-Intensification	France	III	Definitive	4/8	109	VERY ACCEL RT	56	CIS 5FU RT	53	64	NR	0.95 (0.63–1.43)	0.700	NR	NR	NR	Government
Gregoire et al. (2011)^74^	EORTC 24063 (1839IL/0706)	International (26 centres: Belgium, Germany, India, Poland, Serbia, Taiwan, USA)	II	Definitive	5/8	226	CIS 3W	116	GEFIT CIS RT	110	70	NR	1.06 (0.68–1.66)	0.791	88	12	NR	Industry
Bourhis et al. (2012)^75^	GORTEC 99-02	France/Belgium	III	Definitive	6/8	560	VERY ACCEL RT	281	CARBO 5FU RT	279	64.8	62.4	0.81 (0.67–0.99)	0.040	NR	NR	NR	Government
Janssens et al. (2012)^76^	ARCON Phase III	Netherlands/United Kingdom	III	Definitive	6/8	345	ACCEL RT	174	ARCON ACCEL RT	171	68	44	1.03 (0.73–1.46)	0.860	78	22	NR	Government
Gupta et al. (2013)^77^	NR	India	NR (RCT)	Definitive	3/8	138	CIS 5FU RT	67	CIS CAPE RT	71	70	36	1.67 (0.72–3.89)	0.229	84	16	NR	nan
Harrington et al. (2013)^78^	NCT00387127	International (9 countries)	II	Definitive	4/8	67	CIS 3W	33	LAPAT CIS RT	34	70	NR	0.90 (0.44–1.84)	0.382	NR	NR	NR	Industry
Martins et al. (2013)^79^	NCT00410826	United States	II	Definitive	4/8	204	CIS 3W	105	ERLO CIS RT	99	70	26	1.05 (0.56–1.98)	0.880	81	19	Mixed	Industry
Rishi et al. (2013)^80^	PGIMER Oropharynx CBRT versus CRT	India	III	Definitive	4/8	216	CIS 3W	106	CBRT RT	110	66	NR	0.73 (0.34–1.18)	0.170	95	5	NR	nan
Nguyen-Tan et al. (2014)^81^	RTOG 0129	USA/Canada	III	Definitive	6/8	743	CIS 3W	372	AFX CIS RT	371	70	94.8	0.96 (0.79–1.18)	0.370	85.6	14.4	Mixed	Government
Gupta et al. (2015)^82^	Indian Accelerated RT versus CCRT	India	III	Definitive	3/8	133	ACCEL RT	66	CIS WK	67	66	12	1.02 (0.60–1.74)	0.935	NR	NR	NR	Government
Hassan Metwally et al. (2015)^83^	IAEA-HypoX	International (6 centres)	III	Definitive	4/8	82	ACCEL RT	43	NIMO ACCEL RT	39	66–70	19	0.65 (0.35–1.19)	0.160	NR	NR	NR	Government
Mesia et al. (2015)^84^	CONCERT-1	International (9 countries)	II	Definitive	4/8	153	CIS 3W	64	PANI CIS RT	89	70	25.4	1.63 (0.88–3.02)	0.120	90	10	Mixed	Industry
Rodriguez et al. (2015)^85^	NR	United States	III	Definitive	5/8	69	CIS 3W	35	CIS 5FU RT	34	70–74.4	41	5.30 (1.30–21.60)	0.013	91	9	68%	Academic
Dietz et al. (2018)^29^	DeLOS-II	Germany/Austria	II	Definitive (larynx preservation)	4/8	173	INDUCTION RT	85	INDUCTION CETUX RT	88	69.6	24	1.04 (0.60–1.78)	0.779	85.9	14.1	NR	Merck/Sanofi
Geoffrois et al. (2018)^86^	GORTEC 2007-02	France/Belgium/Switzerland	III	Definitive	6/8	360	CARBO 5FU RT	179	TPF CETUX RT	181	70	33.6	1.12 (0.86–1.46)	0.390	85	15	22%	Academic
Janoray et al. (2020)^87^	TREMPLIN	France	II	Definitive	5/8	116	CIS 3W	60	CETUX RT	56	70	66	1.04 (0.55–1.98)	NR	NR	NR	NR	Government
Merlano et al. (2020)^88^	INTERCEPTOR (NCT00999700)	Italy	III	Definitive	5/8	242	CIS 3W	121	TPF CETUX RT	121	70	NR	1.05 (0.71–1.54)	0.800	80	20	NR	None (academic)
Borel et al. (2024)^89^	CisFRad (GORTEC 2015–02)	France	II	Mixed	5/8	122	CIS 3W	64	CIS FRAC 3W	58	70	48	0.86 (0.50–1.47)	0.600	NR	NR	NR	NR
Mell et al. (2024)^90^	NRG-HN004	USA	II/III	Definitive	5/8	186	CETUX RT	63	DURVA RT	123	70	NR	1.30 (0.74–2.28)	NR	NR	NR	Mixed	Government
Bourhis et al. (2025)^21^	GORTEC 2018–01 NIVOPOST-OP	International (France/Spain/Poland/Belgium/Greece/Switzerland)	III	Adjuvant	5/8	666	CIS 3W	334	NIVO CIS 3W	332	66	30.3	0.84 (0.62–1.15)	NR	NR	NR	5%	Industry
Eriksen et al. (2025)^91^	DAHANCA 19	Denmark/Norway	III	Definitive	7/8	608	NIMO CIS WK	307	ZALU NIMO CIS WK	301	66–68	59	1.21 (0.91–1.61)	NR	82	18	55%	Industry
Zandberg et al. (2025)^92^	HCC 15-132	United States	II	Definitive	4/8	80	IMMUNO CRT	41	SEQ PEMBRO CIS WK	39	70	43	0.51 (0.19–1.37)	0.170	92.7	7.3	53%	Mixed
Grégoire et al. (2025)^93^^a^	NIMRAD-2	International	III	Definitive	5/8	194	ACCEL CIS RT (placebo)	97	NIMORAZ + ACCEL CIS RT	97	70	44	0.92 (0.56–1.51)	0.75	NR	NR	HPV-neg	Public/Academic

NOS, Newcastle-Ottawa Scale (adapted for RCTs); RCT, randomised controlled trial; N (Total), total number of participants; n (Ctrl), number in control arm; n (Exp), number in experimental arm; Male %, proportion of male participants at study level (per Sex and Gender Equity in Research (SAGER) guidance); Female %, proportion of female participants; F/U, follow-up; mo, months; HR, hazard ratio; CI, confidence interval; OS, overall survival; RT, radiotherapy; Gy, Grey; NR, not reported; Ctrl, control arm; Exp, experimental arm; HPV, human papillomavirus; OP, oropharynx. Drug and schedule abbreviations: CIS, cisplatin; WK, weekly; 3W, three-weekly; 5FU, 5-fluorouracil; CARBO, carboplatin; CAPE, capecitabine; CETUX, cetuximab; NIMO, nimotuzumab; NIMORAZ, nimorazole; PANI, panitumumab; LAPAT, lapatinib; ERLO, erlotinib; GEFIT, gefitinib; ZALU, zalutumumab; DURVA, durvalumab; NIVO, nivolumab; NIVOPOST, nivolumab post-operative regimen; PEMBRO, pembrolizumab; SEQ, sequential; IMMUNO, immunotherapy; TPF, docetaxel plus cisplatin plus 5-fluorouracil induction; HBO, hyperbaric oxygen. Fractionation and delivery abbreviations: ACCEL, accelerated fractionation; VERY (VERY ACCEL), very accelerated; AFX, altered fractionation; FRAC, fractionated; CBRT, concomitant boost radiotherapy; CRT, chemoradiotherapy; CCRT, concurrent chemoradiotherapy; ARCON, accelerated radiotherapy with carbogen and nicotinamide. Trial identifiers retained as originally reported (e.g., DAHANCA, GORTEC, EORTC, RTOG, NRG, IAEA, INTERCEPTOR, TREMPLIN, CONCERT, NIMRAD, KEYNOTE, HN004, PGIMER) refer to the sponsoring cooperative group or protocol number. HR < 1 favours experimental arm; HR > 1 favours control arm. These 23 studies were included in sensitivity analyses only (not in the primary 42-study OS-reporting NMA) due to: Phase IIb design with limited sample size, asymmetric induction chemotherapy, accelerated or altered fractionation RT schedules, dead-end nodes without network connectivity to the primary network, or structural disconnection from the main network graph.

a NIMRAD-2 (Grégoire et al. 2025): included in this table for reference completeness only. Its accelerated-fractionation backbone with non-randomised cisplatin scheduling did not map to any standard treatment node, so both arms formed a disconnected sub-network and were excluded from all quantitative analyses; findings are discussed narratively in the Discussion.

Toxicity was analysed descriptively by extracting Grade 3–4 adverse-event rates and treatment-related death rates, summarised by treatment node and visualised using heatmaps and efficacy–toxicity scatter plots.

All analyses were performed in R (version 4·5·1). Key packages: netmeta (version 3·2) for the frequentist NMA; gemtc (version 1·0-2) with rjags (version 4·17) for the Bayesian NMA; meta (version 8·2-0) and metafor (version 4·8-0) for pairwise meta-analyses and meta-regression; and ggplot2 (version 3·5-2) for visualisation. Statistical significance for pairwise treatment comparisons was set at two-sided p < 0.05. All analyses were pre-specified in the study protocol; no post-hoc analyses were undertaken during peer review.

### Ethics

This study is a systematic review and network meta-analysis of aggregate summary data extracted from previously published randomised controlled trials. No new patient-level or individually identifiable data were collected or analysed. In accordance with institutional and national guidance, formal ethics-committee approval was therefore not required for this secondary analysis of publicly available de-identified aggregate trial data, and the requirement for written informed consent was likewise waived on the same grounds. All included primary trials had been conducted with local ethics approval and informed consent as documented in their respective publications and were performed in accordance with the Declaration of Helsinki. This systematic review adhered to the ethical principles of the Declaration of Helsinki throughout.

### Role of the funding source

There was no funding source for this study.

## Results

The search identified 2830 records (PubMed 1391; Embase 923; Cochrane CENTRAL 418; ClinicalTrials.gov 98). After removing 1428 duplicates, 1402 records were screened; 1092 were excluded as irrelevant. Of 310 articles assessed for eligibility, 250 were excluded (not a randomised controlled trial (RCT), n = 63; wrong population/no concurrent radiosensitiser, n = 92; no OS data/duplicate/other criteria, n = 95). Snowballing yielded 7 additional studies. In total, 67 studies were included: 44 in the primary network meta-analysis (42 of which report overall survival) and 23 in sensitivity analyses. One additional trial (Grégoire et al. 2025, NIMRAD-2(43)) could not be integrated into either the primary or sensitivity network because its treatment regimens did not map to existing network nodes (Fig. 1).

**Fig. 1:**
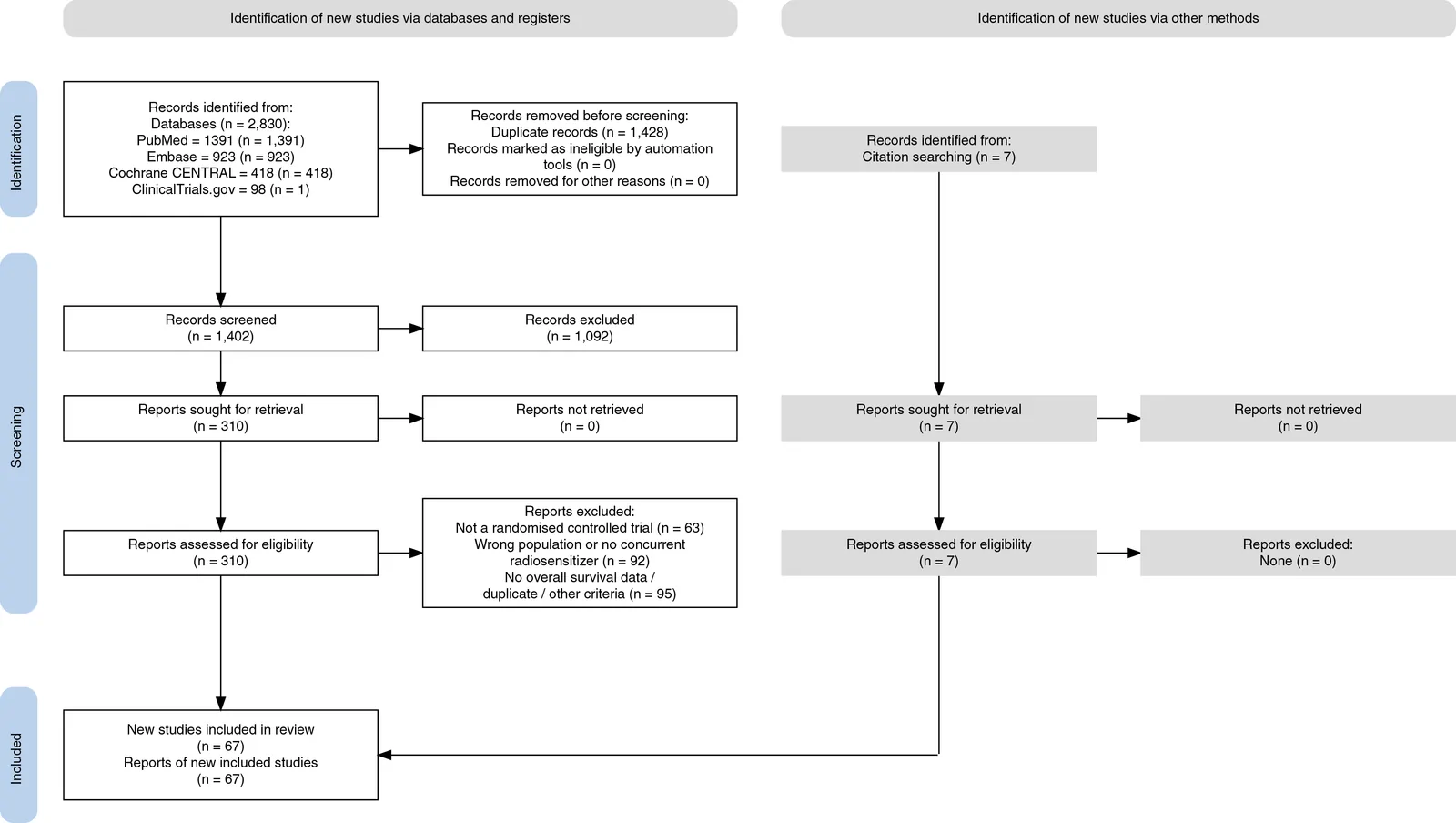
PRISMA 2020 flow diagram of the study selection process. Abbreviations: PRISMA, Preferred Reporting Items for Systematic reviews and Meta-Analyses; NMA, network meta-analysis; RCT, randomised controlled trial; HNSCC, head and neck squamous cell carcinoma; OS, overall survival.

The 42 OS-reporting primary NMA studies (44 main-NMA trials total, of which 2—Overgaard 1998^30^ and Rischin 2021^16^—contribute non-OS endpoints) enrolled 13,768 patients (1977–2025); the sensitivity network adds a further 23 trials contributing 5759 patients, for a combined dataset of 67 randomised controlled trials and 19,527 patients. Most were Phase III (35/42; 83.3% of OS-reporting trials), spanning >32 countries across major cooperative groups (RTOG; the European Organisation for Research and Treatment of Cancer (EORTC); the Groupe d'Oncologie Radiothérapie Tête Et Cou (GORTEC); and the Danish Head and Neck Cancer Group (DAHANCA)). Settings included definitive chemoradiotherapy (CRT) (30 studies), adjuvant postoperative CRT (6 studies), and mixed (3 studies). HPV status was unreported in 30/49 (61%) of the primary-network data rows. Several studies contributed multiple outcome rows (progression-free survival (PFS), locoregional control (LRC), disease-free survival (DFS)). Detailed characteristics are in Table 1.

The primary network comprises 24 treatment nodes with 27 unique direct treatment-pair comparisons from 42 OS-reporting studies (Fig. 2). RT alone was the reference (22 connections), with hub nodes at cisplatin three-weekly (21 connections), cisplatin weekly (Supplementary Figure S2),^12^ and cetuximab plus RT.^9^ The remaining 21 nodes were connected through only one or two comparisons each. Nodes were colour-coded by class: platinum (blue), EGFR-targeted (orange), immunotherapy (green), hypoxic modifiers (red), and other (purple). Four multi-arm trials^27^^,^^28^^,^^54^^,^^75^ retained only relevant pairwise comparisons. With all sensitivity studies, the network expanded to ∼41 nodes.

**Fig. 2:**
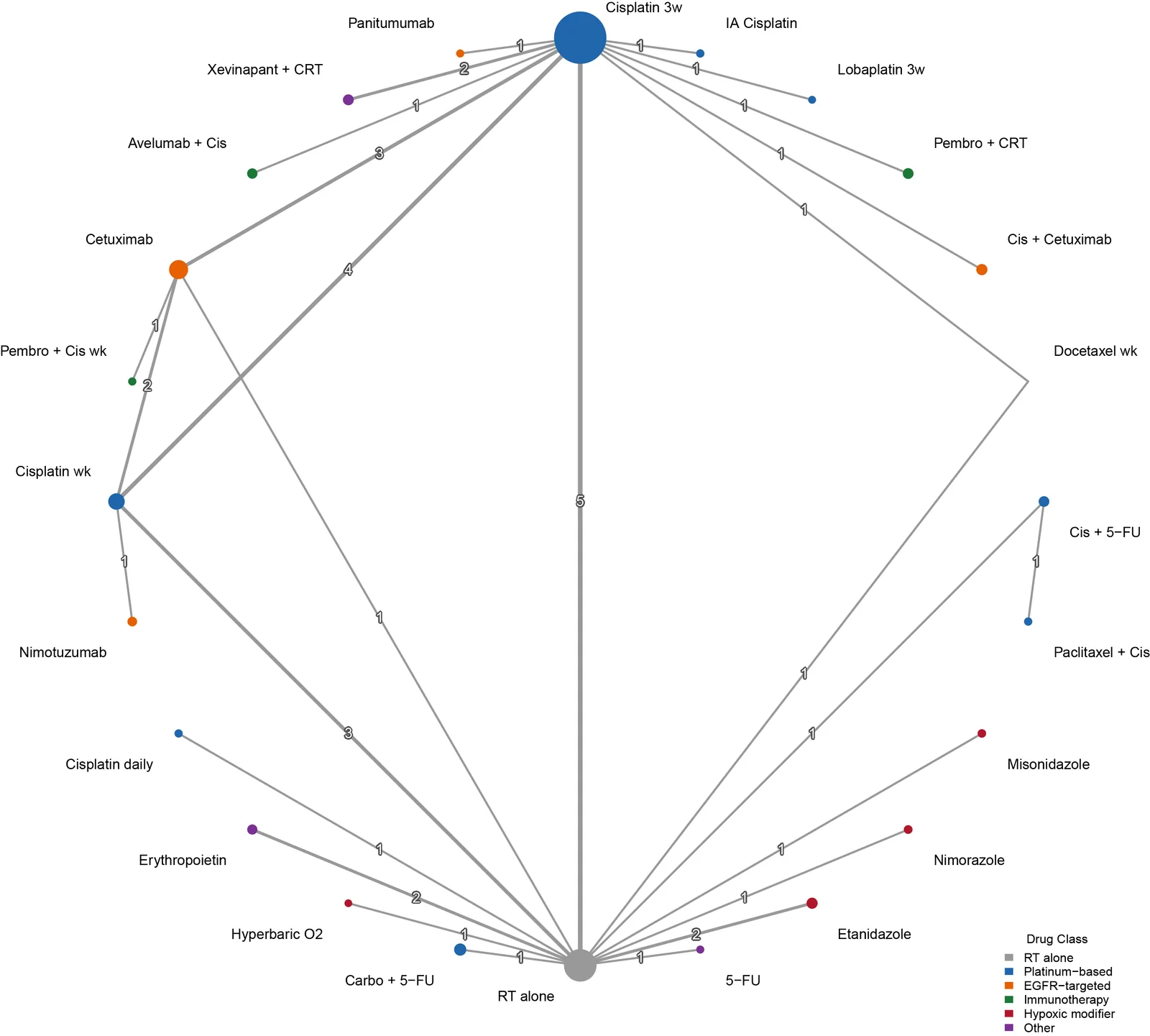
Network plot of the primary network meta-analysis comprising 24 treatment nodes and 27 direct treatment-pair comparisons from 42 OS-reporting randomised controlled trials. Node size is proportional to the number of randomised participants contributing to that node; edge thickness is proportional to the number of direct comparisons between two nodes. Colours indicate drug class (blue, platinum agents; orange, EGFR-targeted; green, immunotherapy; red, hypoxic modifiers; purple, other). Abbreviations: CIS, cisplatin; RT, radiotherapy; WK, weekly; 3W, three-weekly; CETUX, cetuximab; NIMO, nimotuzumab; 5FU, 5-fluorouracil; PAC, paclitaxel; DOC, docetaxel; CARBO, carboplatin; OS, overall survival.

RoB 2 assessment across all 63 assessed studies showed 50/63 (79%) with some concerns, 5/63 (8%) low risk, and 8/63 (13%) high risk (Supplementary Figures S1a and S1b, and Supplementary Table S2). All studies had low risk for outcome measurement (D4), as OS is objective. The most frequent concern was deviations from interventions (RoB 2 domain 2 (D2); 52/63, 82.5%, with some concerns) due to open-label designs. Eight high-risk studies were Rodriguez,^85^ Merlano,^88^ Ghosh-Laskar,^54^ Bourhis NIVOPOSTOP,^23^ Gupta 2013,^82^ Rishi,^80^ Gupta 2015,^77^ and Hassan Metwally.^83^ CINeMA assessment indicated moderate-to-high confidence for hub node comparisons and lower confidence for single-study dead-end nodes. Restricted to the 44 primary-NMA trials, 39 of 44 (89%) had some concerns for risk of bias.

Four treatments showed significant OS benefit versus RT alone (Fig. 3): cisplatin plus 5-fluorouracil (5-FU) (HR 0.35; 95% CI 0.18–0.66),^18^^,^^78^ nimotuzumab plus weekly cisplatin (0.48; 0.29–0.81), cisplatin weekly (0.57; 0.44–0.74), and three-weekly cisplatin (0.79; 0.65–0.95). Paclitaxel plus cisplatin showed a borderline result (0.42; 0.18–1.01), while xevinapant plus CRT (0.76; 0.48–1.20) did not reach significance.

**Fig. 3:**
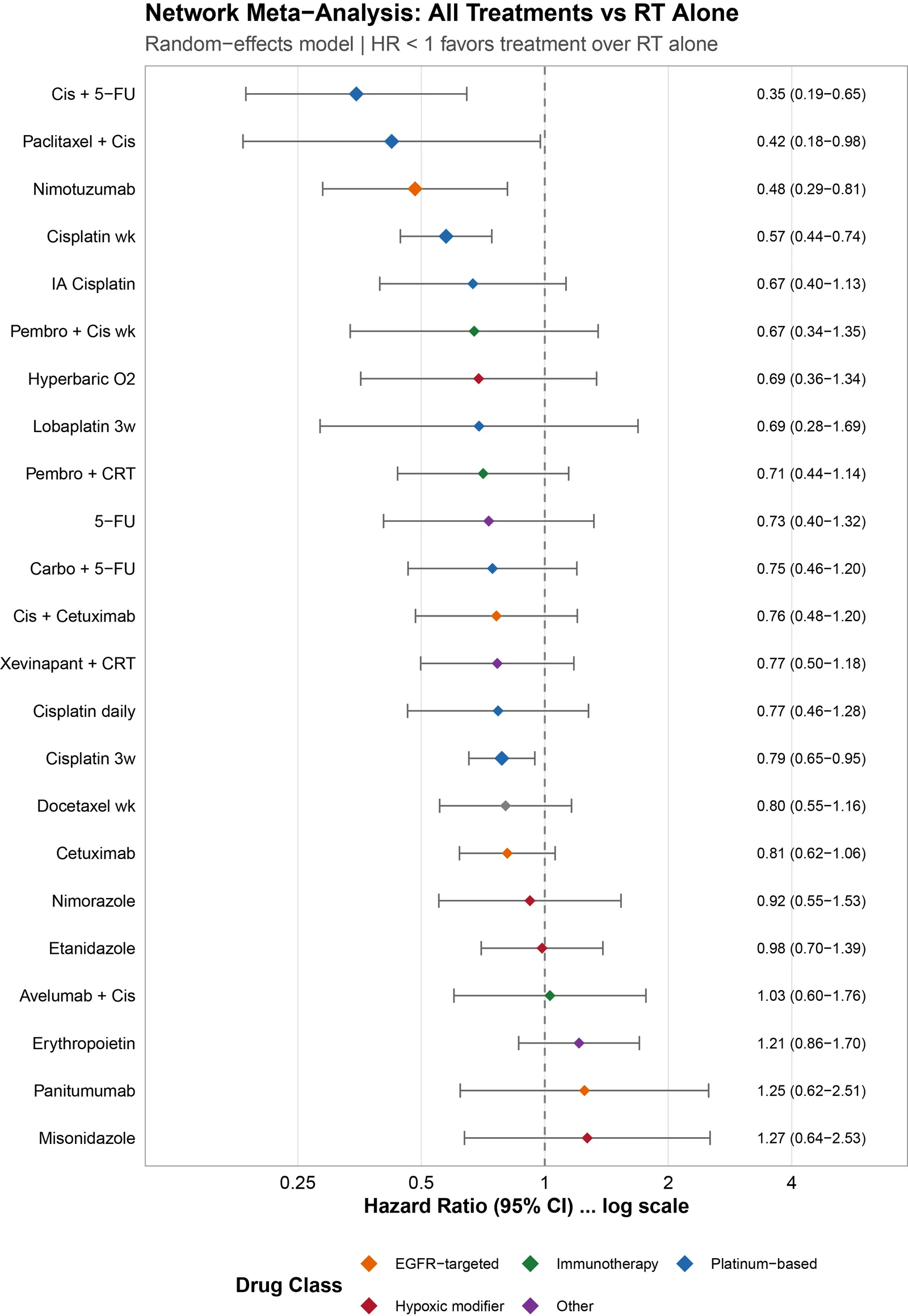
Forest plot comparing all treatments versus radiotherapy alone (RT_ALONE) for overall survival. Points show pooled hazard ratios (HR) from the random-effects network meta-analysis; horizontal bars show 95% confidence intervals. HR < 1 favours the experimental treatment; HR > 1 favours radiotherapy alone. The vertical line at HR = 1 denotes the line of no effect.

Cetuximab plus RT approached but did not reach significance (HR 0.81; 0.64–1.03).^73^ Pembrolizumab plus weekly cisplatin (0.67; 0.35–1.29), intra-arterial cisplatin (0.68; 0.42–1.09), and pembrolizumab plus CRT (0.72; 0.47–1.10) favoured treatment but with CIs crossing unity. Docetaxel weekly (0.75; 0.50–1.12),^94^ carboplatin plus 5-FU (0.75; 0.49–1.13),^67^ and cisplatin plus cetuximab (0.78; 0.52–1.15) were similarly non-significant. Nimorazole (0.92; 0.58–1.46) and etanidazole (0.98; 0.73–1.33) showed no benefit. Erythropoietin showed a non-significant unfavourable point estimate (1.21; 0.90–1.64).

Two treatments were significantly superior to cisplatin three-weekly (Fig. 4): cisplatin plus 5-FU (HR 0.44; 95% CI 0.23–0.83) and cisplatin weekly (0.73; 0.58–0.93). Nimotuzumab plus weekly cisplatin showed a favourable but non-significant point estimate (0.61; 0.35–1.07). Xevinapant plus CRT was not significantly different (0.96; 0.64–1.45). The weekly cisplatin comparison is particularly relevant, showing a 27% reduction in hazard of death relative to three-weekly dosing in the network-pooled estimate (interpretation should account for the underlying trials' non-inferiority designs—see Discussion).

**Fig. 4:**
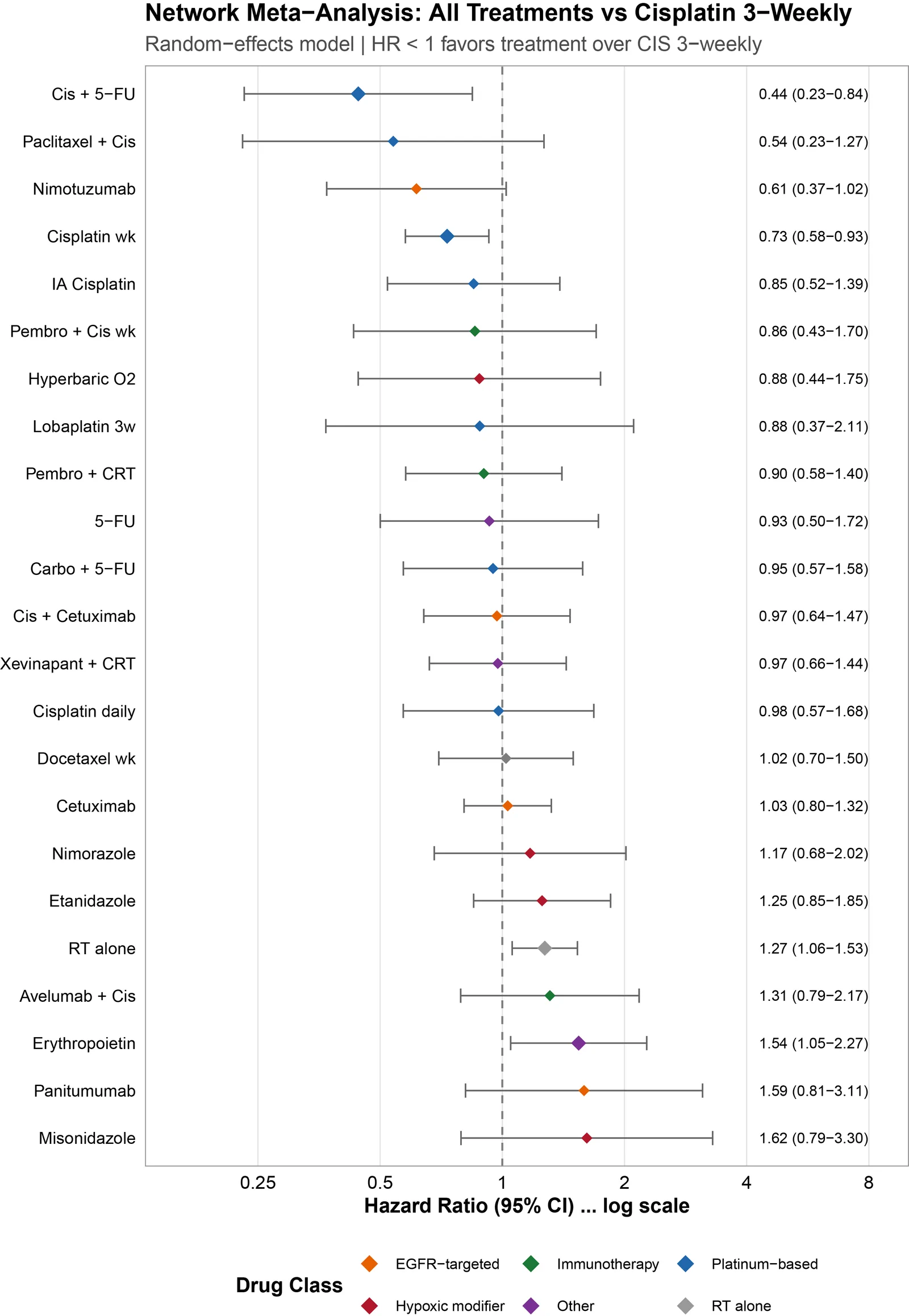
Forest plot comparing all treatments versus cisplatin three-weekly (CIS_3W) for overall survival. Points show pooled hazard ratios (HR) from the random-effects network meta-analysis; horizontal bars show 95% confidence intervals. HR < 1 favours the experimental treatment; HR > 1 favours cisplatin three-weekly. Vertical line at HR = 1 denotes the line of no effect.

Cetuximab plus RT showed no significant difference compared with cisplatin three-weekly in the network estimate (HR 1.01; 0.81–1.27); equivalence cannot be formally claimed because the confidence interval is wider than typical equivalence margins. Individual head-to-head trials consistently favoured cisplatin, including De-ESCALaTE(14), RTOG 1016,^15^ and TROG 12.01 (failure-free survival (FFS) HR 3.0; 1.2–7.7)^16^; the network estimate integrates these direct comparisons with indirect evidence through the Bonner trial.^50^ Avelumab plus cisplatin showed a non-significant unfavourable trend (1.31; 0.83–2.07). RT alone (1.25; 1.05–1.48) and erythropoietin (1.52; 1.07–2.15) were significantly worse than cisplatin three-weekly.

The league table (Fig. 5, Supplementary Table S3) presents all 276 pairwise hazard ratios among the 24 treatments, ordered by P-score. Significant separations were concentrated among top-ranked versus bottom-ranked treatments.

**Fig. 5:**
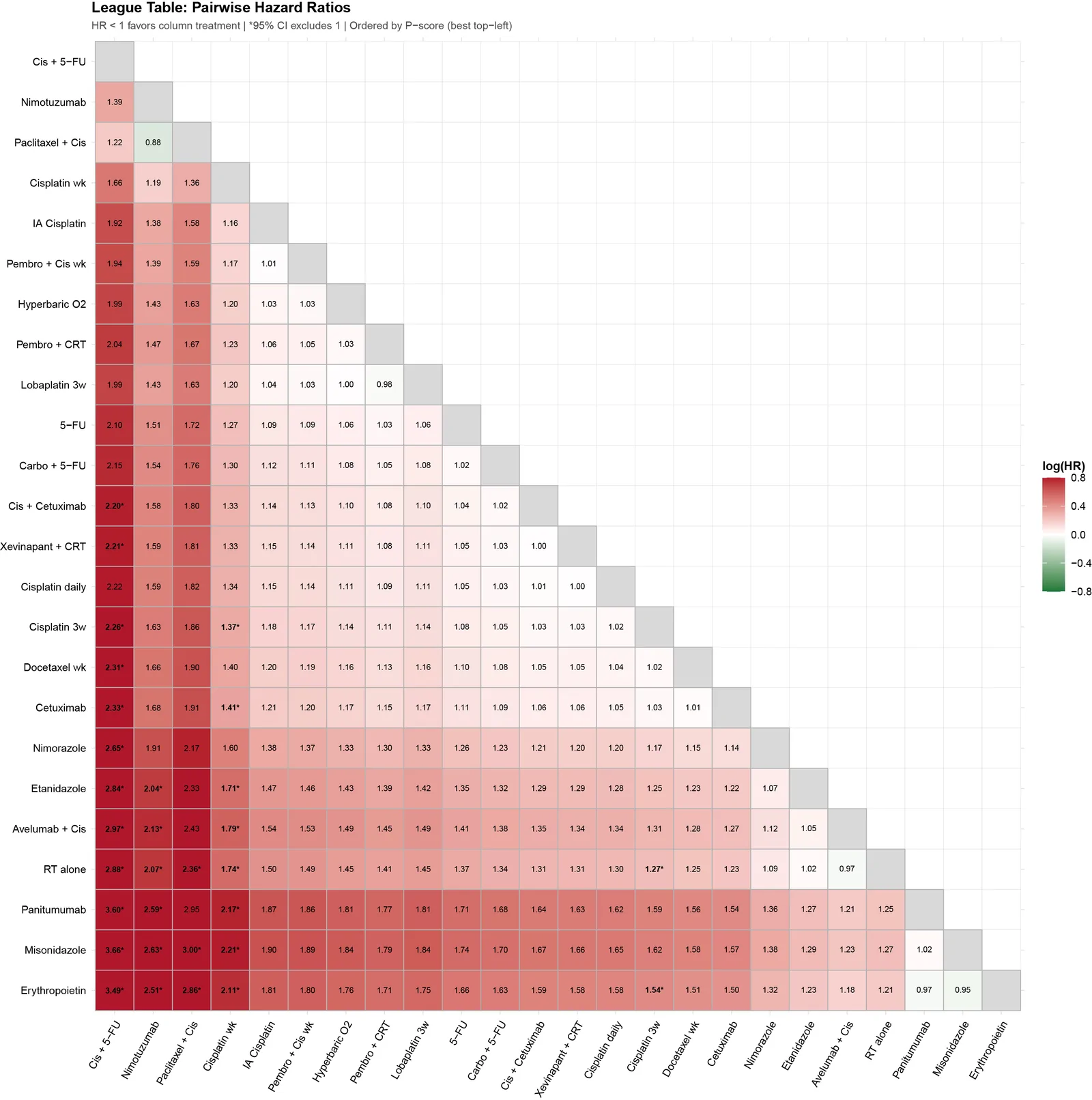
League table heatmap of all pairwise hazard ratios (HR) among the 24 treatments in the primary network. Green cells indicate that the row treatment is superior to the column treatment (HR < 1 with 95% CI excluding 1); red cells indicate the column treatment is superior; grey cells indicate no significant difference. Cell entries show HR (95% CI).

Treatment rankings showed excellent concordance between frequentist P-score and Bayesian SUCRA (mean absolute difference 0.4%; Fig. 6, Supplementary Table S4), providing strong methodological confidence in the treatment ordering.

**Fig. 6:**
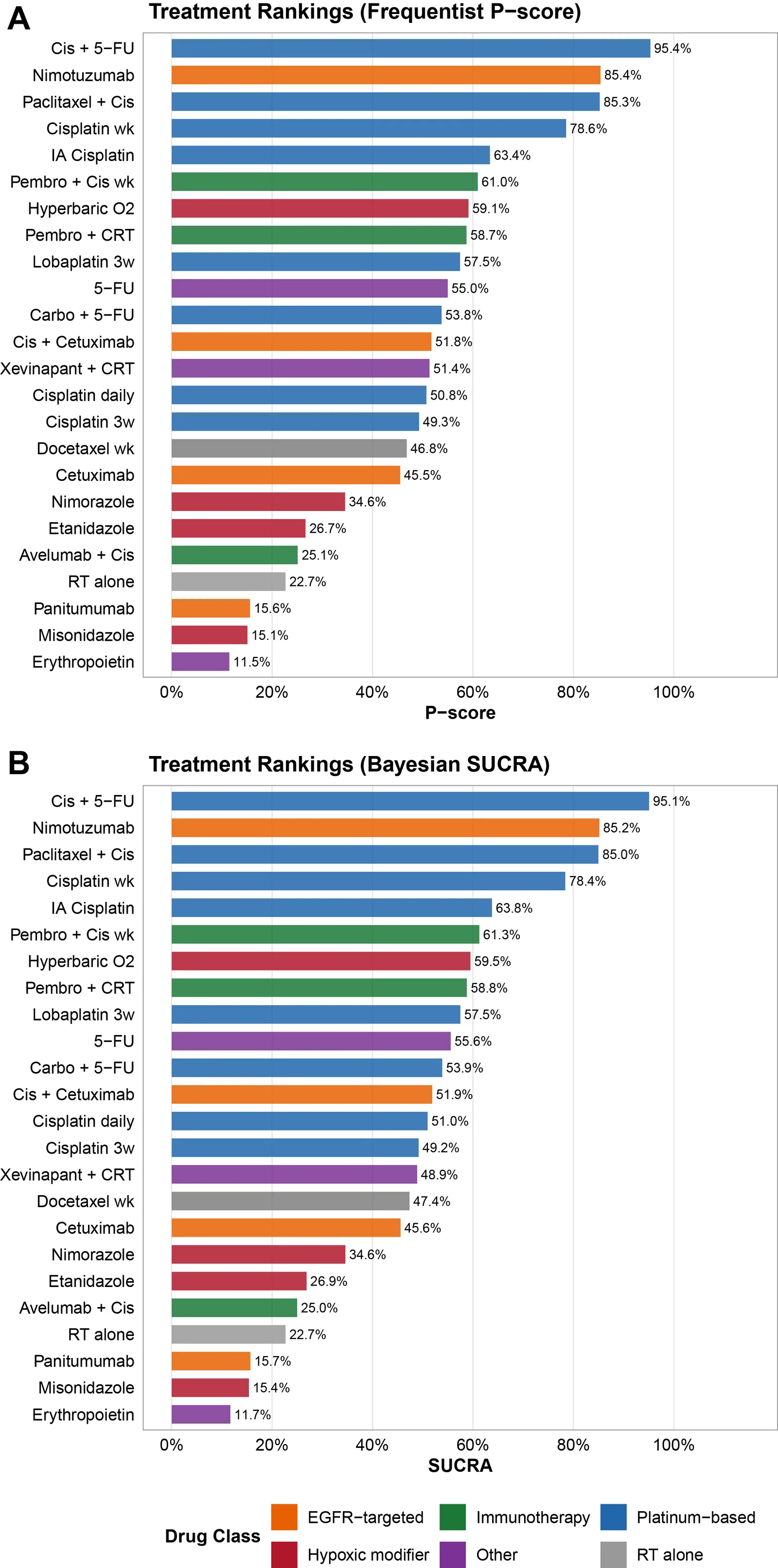
Treatment rankings by P-score (Panel A, frequentist) and SUCRA (Panel B, Bayesian) for all 24 treatments. Values range from 0 to 100, with higher values indicating higher probability of being the best treatment. Mean absolute difference between the two ranking methods was 0.4 percentage points, indicating concordance across estimation frameworks. Abbreviations: SUCRA, Surface Under the Cumulative Ranking curve.

The top five treatments by P-score (SUCRA) were: cisplatin plus 5-FU (94.8%, 94.8%), paclitaxel plus cisplatin (84.4%, 84.5%), nimotuzumab plus weekly cisplatin (84.2%, 84.3%), cisplatin weekly (77.5%, 77.6%), and intra-arterial cisplatin (62.1%, 62.2%). Xevinapant plus CRT ranked 13th (51.9%). Cisplatin three-weekly ranked 15th (47.6%) and cetuximab plus RT 16th (45.8%). The lowest-ranked treatments included misonidazole (14.8%), panitumumab (14.4%), and erythropoietin (11.3%). RT alone ranked 20th (23.5%).

Bayesian models achieved excellent convergence (all Gelman-Rubin estimates below 1.05) in both primary and full sensitivity analyses.

The network shows moderate-to-substantial heterogeneity (I^2^ = 48.9%, τ^2^ = 0.0360, Cochran Q = 37.15, df = 19, p = 0.008), in keeping with typical clinical oncology network meta-analyses spanning four decades of trials with evolving radiotherapy techniques and (full league table of pairwise hazard ratios in Supplementary Table S6, per-edge heterogeneity summary in Supplementary Table S9) patient populations. Multi-study direct comparisons within the network remain internally consistent (I^2^ ≈ 0% within most edges).

The global design-by-treatment interaction test revealed no significant inconsistency (Q = 0.66, df = 3, p = 0.88).

The net heat plot (Supplementary Figure S3) corroborated the non-significant global test.

Node-splitting analysis (Supplementary Figure S11 and Supplementary Table S8) confirmed local consistency for all 6 comparisons with both direct and indirect evidence (all p > 0.45); the corresponding direct-evidence contribution plot is provided in Supplementary Figure S10.

All sensitivity analyses confirmed primary findings (Supplementary Figure S4, Supplementary Table S5). Expansion to the full 66-study network preserved top-five treatment ranking, with maximum P-score shifts below 3 percentage points. Treatment effect estimates remained stable across all network configurations.

Exclusion of the Rodriguez et al.^85^ outlier (HR 5.3, 9 deaths, standard error (SE) = 0.717) had minimal impact. Removal of asymmetric induction studies (Merlano^88^ and Geoffrois^86^) did not alter conclusions. Restriction to definitive-setting studies yielded consistent rankings.

Subgroup analysis evaluated effect modification by treatment setting (Supplementary Figures S5 and S6, Supplementary Table S5); a full pairwise subgroup forest plot is provided in Supplementary Figure S12. Of 42 OS-reporting primary studies, 30 were definitive, 7 adjuvant (Bernier, Cooper, Bachaud, Wozniak, Kiyota, Noronha, Jiang) (Bernier,^48^ Cooper,^51^ Bachaud^43^), Wozniak,^60^ Kiyota,^17^ Noronha^69^)), and 4 mixed-setting (Henke,^47^ Hoskin,^49^ Patil,^64^ Zhang^66^). Meta-regression showed no setting effect modification (β = 0.021, p = 0.861). Restriction to definitive-setting studies preserved all top-five rankings (maximum P-score shifts <2.6 percentage points).

Pooled adjuvant cisplatin three-weekly versus RT alone (Bernier,^48^ Cooper,^51^ Wozniak^60^) showed significant benefit (fixed-effect HR 0.83; 0.70–0.99, p = 0.039).^48^ to 1.03.^60^ Consistency between full and definitive-only networks supports pooling.

Meta-regression examined four covariates: oropharyngeal cancer proportion, laryngeal cancer proportion, treatment setting, and enrolment start year (full covariate table in Supplementary Table S12).

Oropharyngeal cancer proportion was significantly associated with treatment effect (β = 0.006, p = 0.021; 26 studies; R-squared 13.6%). Each 10 percentage-point increase in oropharyngeal proportion shifted log(HR) by 0.06 toward the null (Supplementary Figure S13, subsite bubble plot). This finding is consistent with the HPV confound: HPV-positive oropharyngeal cancers have favourable prognosis regardless of treatment, potentially diluting apparent radiosensitiser benefit in enriched populations.

Neither laryngeal cancer proportion (β = −0.002, p = 0.633) nor treatment setting (definitive versus adjuvant; β = 0.021, p = 0.861) showed significant associations, supporting the pooling of studies across settings enrolment start year was significantly associated with treatment effect (continuous β = 0.009, p = 0.028, R^2^ = 11.7%; post-2005 versus pre-2005 categorical β = 0.23, p = 0.010, R^2^ = 14.7%), with later trials showing smaller incremental benefits (Supplementary Figures S14 [bubble plot] and S15 [era-stratified forest plot])—likely reflecting improved baseline radiotherapy delivery (intensity-modulated techniques) in the modern era. Era-stratified NMAs using a 2005 cutoff showed cisplatin three-weekly versus RT alone HR 0.81 (0.68–0.96) pre-2005 versus HR 1.03 (0.45–2.36) post-2005 (interaction p = 0.011). Because of this significant era effect, we present pre-2005 and post-2005 stratified network estimates at co-equal prominence with the full-period analysis (Supplementary Table S13); present-day treatment recommendations should be informed primarily by the post-2005 evidence. This temporal trend most plausibly reflects the transition from two-dimensional or three-dimensional conformal radiotherapy in earlier trials to intensity-modulated radiotherapy in contemporary trials, with improved baseline radiotherapy delivery narrowing the incremental benefit of adding a radiosensitiser.

The comparison-adjusted funnel plot (Supplementary Figure S7) showed no major asymmetry. A single outlier (Rodriguez et al.^85^) was identified. The inclusion of both positive and negative trials mitigates publication bias risk.

Toxicity data from 26 studies covering 16 treatment nodes are summarised in Supplementary Figure S8 and Supplementary Table S10. Severe mucositis rates (pooled across contributing trials) were highest for carboplatin plus 5-FU (71.0%), nimotuzumab plus weekly cisplatin (66.7%), cetuximab plus RT (48.0%), cisplatin weekly (46.2%), and cisplatin three-weekly (39.6%), versus RT alone (26.6%).

Cetuximab plus RT showed the highest severe dermatitis rate (56.1%), reflecting characteristic anti-EGFR skin toxicity. Grade 3–4 dysphagia was highest for cisplatin weekly (41.0%) and cetuximab plus RT (33.5%). Neutropenia was most frequent with cisplatin three-weekly (24.1%).

Treatment-related death rates (Supplementary Table S11) were 2.2% for platinum-based regimens, 3.7% for EGFR-targeted therapies, and 1.0% for immunotherapy combinations. The lower mortality with immunotherapy is notable, though longer follow-up may reveal delayed toxicities.

The efficacy-toxicity scatter plot (Supplementary Figure S9) visualises the trade-off between OS benefit and acute toxicity for nine treatment nodes, with the lower-left quadrant representing the most favourable risk–benefit profile.

Cisplatin plus 5-FU occupied the most favourable position, combining the lowest HR (0.35) with moderate mucositis (38%). Nimotuzumab plus weekly cisplatin showed high efficacy (HR 0.49) but substantial mucositis (66.7%). Cisplatin weekly (HR 0.58) had high toxicity (71.6%), while cetuximab plus RT (HR 0.81) had a qualitatively different profile dominated by dermatitis rather than nephro/ototoxicity. Immunotherapy combinations showed a promising lower–toxicity profile. The plot demonstrates that the most efficacious treatments are not necessarily the most toxic. Full trial-level details for all included primary and sensitivity studies are presented in Table 1 and 2.^39^, ^40^, ^41^, ^42^^,^^44^, ^45^, ^46^^,^^52^^,^^53^^,^^55^, ^56^, ^57^, ^58^, ^59^^,^^61^, ^62^, ^63^^,^^74^^,^^79^^,^^81^^,^^84^^,^^87^^,^^89^, ^90^, ^91^

## Discussion

This network meta-analysis synthesises evidence from 44 primary-NMA trials (13,768 patients; 42 with OS endpoints; 24 treatments)—spanning 1977–2025. Four treatments demonstrated significant OS benefit versus RT alone, with cisplatin-based regimens consistently dominating rankings. Treatment hierarchy was robust across frequentist and Bayesian frameworks (P-score/SUCRA difference 0.4%), with no global inconsistency (p = 0.88).

Our findings reinforce and extend the MACH-NC, which reported HR 0.81 for concurrent chemoradiotherapy based on 93 trials and 17,346 patients.^7^^,^^8^ Our cisplatin three-weekly estimate (HR 0.80; 0.67–0.95) closely matches, providing external validation. However, by disaggregating into 24 distinct strategies, we reveal substantial efficacy heterogeneity masked in prior pooled analyses. The top-ranked combinations (HR 0.35–0.48) demonstrate considerably greater benefit than the pooled average, indicating that specific regimen selection matters. The MARCH meta-analysis showed modest benefit for altered fractionation.^9^^,^^95^ Our network does not include altered-fractionation-only arms without a concurrent radiosensitiser, so we cannot directly compare cisplatin-based chemoradiotherapy against radiotherapy intensification alone; head-to-head evidence for that specific comparison remains the domain of the MARCH meta-analysis.

The advantage of weekly over three-weekly cisplatin in our network estimate (HR 0.73; 0.58–0.93) should be interpreted with caution because the underlying randomised trials (JCOG1008^17^; Noronha long-term update^69^; Nair 2017^70^; Nanda 2019^72^) were all designed as non-inferiority or exploratory comparisons; none was powered to demonstrate weekly superiority and none individually achieved it. Two recent aggregate-data meta-analyses (Szturz et al. 2017^96^; Kloska et al. 2025^97^; De Felice et al. 2021^98^) reached similar conclusions of clinical equivalence rather than weekly superiority. The two recently added Indian Phase IIb trials (Nair 2017^70^; Nanda 2019^72^) each enrolled fewer than 100 patients at a single institution using older cobalt-based radiotherapy, and contribute low-precision direct evidence with wide confidence intervals; their effect estimates favour weekly cisplatin in direction but do not individually reach statistical significance (Supplementary Figure S16 shows the four-trial direct-evidence forest plot). The network estimate gains apparent statistical significance principally from integration of direct and indirect evidence across the broader treatment graph; this is a methodological feature of NMA rather than a head-to-head superiority demonstration. The Noronha long-term update (6.9 years) demonstrated superior locoregional control with three-weekly cisplatin (LRC HR 1.44; 1.03–2.03 favouring three-weekly),^69^^,^^99^ while JCOG1008 reported a non-significant OS trend favouring weekly dosing (HR 0.75; 0.50–1.13).^17^ Our network integrates both direct and indirect evidence across multiple comparisons, providing greater power. The cumulative cisplatin dose hypothesis may partly reconcile these findings: weekly regimens achieve higher completion rates and cumulative doses exceeding 200 mg/m^2^, associated with improved OS.^96^^,^^100^ While these data support consideration of weekly cisplatin as an effective option, three-weekly cisplatin remains a valid standard given its demonstrated locoregional control advantages.

This NMA confirms cetuximab plus RT is not interchangeable with cisplatin-based CRT (HR 1.01; 0.81–1.27 versus cisplatin three-weekly), consistent with De-ESCALaTE 111^14^ and RTOG 1016.^15^ While the Bonner trial^50^ demonstrated improved survival, the network estimate did not reach significance (HR 0.81; 0.64–1.03). ARTSCAN III reinforced cisplatin superiority (HR 1.66; 1.08–2.56).^68^ However, biomarker subgroup data showed marked benefit of cetuximab in p16-positive tumours (HR 0.38) versus none in p16-negative disease (HR 0.93), suggesting a potential role in patients who are HPV-positive and cisplatin-ineligible, requiring prospective validation.

Historical agents provide important context. Nimorazole showed non-significant OS benefit (HR 0.92; 0.58–1.46), consistent with DAHANCA 5 which showed locoregional benefit in pharyngeal tumours.^30^ Misonidazole was limited by neuropathy; etanidazole and hyperbaric oxygen showed no benefit. Erythropoietin showed a non-significant unfavourable point estimate (HR 1.21), consistent with the potential for erythropoietin (EPO) receptor stimulation on tumour cells.^47^ The critical lesson is biomarker-guided selection: the accelerated radiotherapy with carbogen and nicotinamide (ARCON) trial showed carbogen/nicotinamide benefit exclusively in hypoxic tumours (pimonidazole uptake),^76^ and NIMRAD tested nimorazole in tumours identified by a 15-gene hypoxia classifier.^67^ This paradigm, where negative drug classes may benefit molecularly selected populations, has broad implications for personalised radiosensitization. The recently reported NIMRAD-2 trial (Grégoire et al.^93^) provides additional, though non-integrable, evidence supporting nimorazole as a hypoxic radiosensitiser: in 194 patients with HPV-negative locally advanced HNSCC, adjusted locoregional control was significantly improved (HR 0.51; 0.27–0.95) without significant overall survival benefit (adjusted HR 0.92; 0.56–1.51); the trial's accelerated-RT backbone with non-randomised cisplatin scheduling precluded its inclusion in our quantitative network.

Neither immunotherapy combination achieved significant OS benefit. KEYNOTE-412 showed non-significant event-free survival (EFS) (HR 0.83; 0.68–1.03).^16^ JAVELIN Head and Neck 100 was terminated for futility.^19^ NIVOPOSTOP reported positive DFS (HR 0.76; 0.60–0.98) but immature OS.^23^^,^^101^ Treatment-related mortality was lower with immunotherapy (1.0%) than platinum (2.2%) or EGFR agents (3.7%). Radiation-induced lymphodepletion may partly explain these results, and novel sequencing strategies (concurrent versus sequential pembrolizumab)^92^ may overcome this. Biomarker-selected populations may yet benefit.

Xevinapant plus CRT ranked 13th (P-score 51.9%; HR 0.96 versus cisplatin three-weekly; 0.64–1.45). This inhibitor of apoptosis protein (IAP) antagonist restores apoptotic signalling. The Phase II GORTEC 2015-03 showed notable OS benefit (HR 0.47; 0.27–0.84),^65^^,^^102^ but the Phase III TrilynX did not meet its primary EFS endpoint (HR 0.81; 0.61–1.09).^21^ With Phase II and Phase III data now pooled into a single xevinapant node, the combined HR versus RT alone is 0.76 (0.48–1.20), no longer statistically significant. This outcome confirms that early-phase signals require Phase III validation before being considered practice-changing.

Weekly cisplatin achieves high efficacy (HR 0.58) but with substantial Grade 3–4 toxicity (71.6%), predominantly mucositis and dysphagia. Cetuximab plus RT exhibits a different profile dominated by dermatitis (56.1%) rather than renal and haematological complications, making it potentially attractive for patients with pre-existing renal impairment. Cisplatin plus 5-FU showed the best efficacy-toxicity balance. Treatment-related mortality ranged from 1.0% (immunotherapy) to 3.7% (EGFR agents). Treatment selection must integrate toxicity profiles, comorbidities, and patient preferences alongside efficacy rankings. New direct evidence from the Jiang 2024 phase IIb trial^71^ in 224 high-risk postoperative patients with oral cavity squamous cell carcinoma (SCC) showed weekly docetaxel (20 mg/m^2^ × 6) achieved comparable overall survival to three-weekly cisplatin (HR 0.87; 0.55–1.38) with substantially lower haematologic toxicity, broadening the radiosensitiser options for patients eligible for cisplatin who have concerns about toxicity.

Several clinical implications emerge. First, weekly cisplatin appears at least non-inferior to three-weekly cisplatin (network HR 0.73; 0.58–0.93) and may be preferred where toxicity reduction is prioritised, recognising that the underlying randomised trials were designed for non-inferiority rather than superiority. Second, cetuximab should not replace cisplatin, as multiple Phase III trials consistently demonstrate cisplatin superiority. Third, pooled xevinapant data show no significant OS benefit (HR 0.76; 0.48–1.20), confirming the Phase II signal was not sustained. Fourth, the immunotherapy–CRT space remains undefined; NIVOPOSTOP follow-up^23^ and sequencing approaches^92^ are needed. Fifth, biomarker-guided selection should be adopted broadly, with future updates incorporating individual patient data for HPV stratification and de-escalation evidence.^47^^,^^71^^,^^101^^,^^102^

The novelty and added value of this network meta-analysis lie in its scope and integration. Whereas the MACH-NC individual patient-data meta-analysis^7^^,^^8^ established the overall survival benefit of concurrent chemoradiotherapy across broad chemotherapy categories, and prior network meta-analyses in this setting have focused predominantly on chemotherapy and cetuximab (Iocca 2018^11^) or restricted the population to patients who are cisplatin-ineligible (Petrelli 2025^12^), no prior study has simultaneously compared all major radiosensitiser drug classes–platinum agents, fluoropyrimidines, EGFR-targeted antibodies, hypoxic sensitisers, immune-checkpoint inhibitors, and novel agents such as xevinapant and nanoparticle radio-enhancers–within a single coherent analytical framework. The comparison against three recent, largely overlapping meta-analyses of weekly versus three-weekly cisplatin (Szturz et al. 2017^96^; De Felice et al. 2021^98^; Kloska et al. 2025^97^) illustrates the additional resolution the network framework provides: our estimate of HR 0.73 (0.58–0.93) integrates direct and indirect evidence across the broader treatment graph, whereas the aggregate-data pairwise meta-analyses draw only on the small number of head-to-head trials and therefore rest on the same underlying non-inferiority evidence base. Our analysis also incorporates trials that had not yet been published or were not reflected in earlier meta-analyses, including KEYNOTE-689,^22^ NIVOPOSTOP,^23^ TrilynX,^21^ ARTSCAN III,^68^ the Noronha long-term update,^69^ and the recently identified Nair 2017,^70^ Jiang 2024,^71^ and Nanda 2019^72^ trials.

Beyond the current evidence, several future directions deserve emphasis. The absence of overall survival benefit from checkpoint–inhibitor combinations in KEYNOTE-412(18), JAVELIN Head and Neck 100,^19^ and COMPARE^20^ contrasts with the strong biological rationale for combining PD-(L)1 blockade with radiotherapy through immunogenic cell death, and points to radiation-induced lymphodepletion as a plausible mechanism of failure. Low-dose immunotherapy strategies–for example reduced-dose or attenuated-frequency programmed cell death protein 1 (PD-1) blockade delivered concurrently with radiotherapy to preserve circulating lymphocytes, and sequencing optimisations comparing concurrent versus sequential PD-1 blockade (Zandberg 2025, HCC 15–132^92^)–warrant focused prospective investigation, as does the exploration of predictive biomarkers to identify the subgroup most likely to benefit. Emerging radiosensitisation strategies to be evaluated in the next generation of trials include nanoparticle-based platforms (NBTXR3, Phase III NANORAY-312), DNA damage response inhibitors (poly(ADP-ribose) polymerase (PARP), ataxia telangiectasia and Rad3-related (ATR), and DNA-dependent protein kinase (DNA-PK)), and immunoradiotherapy combinations exploiting radiation-induced immunogenic cell death; integration of genomic profiling and hypoxia imaging may enable individualised radiosensitiser–patient matching.

This study has several strengths. First, it is the largest NMA of radiosensitising strategies in HNSCC (24 treatments, 44 primary-analysis trials, and 67 trials in the full sensitivity network (19,527 patients)), providing greater granularity than the MACH-NC.^7^^,^^8^ Second, no inconsistency was detected (I^2^ 40.9%), and rankings were robust across all sensitivity analyses. Third, frequentist and Bayesian frameworks converged (P-score/SUCRA difference 0.4%). Fourth, treatment nodes defined by mechanism and schedule enabled clinically meaningful distinctions. Fifth, integrated toxicity analysis enables efficacy-toxicity trade-off assessment.

Several limitations warrant acknowledgement. First, study-level aggregate data preclude HPV-stratified analyses. Second, trials span 1977–2025; meta-regression confirmed enrolment era modifies effects (continuous β = 0.009, p = 0.028; post-2005 versus pre-2005 β = 0.23, p = 0.010), with the cisplatin three-weekly benefit attenuating substantially in the modern era, likely reflecting improved RT quality.^100^ Third, top-ranked treatments are informed by limited evidence with wide confidence intervals. Fourth, oropharyngeal proportion modified effects (p = 0.021), suggesting HPV as an unmeasured confounder. Fifth, pooling definitive and adjuvant settings showed no effect modification (p = 0.861). Sixth, open-label designs may modestly affect confidence, though sensitivity analyses were stable. Seventh, radiotherapy technique evolved markedly across the included trial era—from two-dimensional or conformal RT in pre-2010 trials to intensity-modulated RT in most contemporary trials—and we could not formally adjust for technique as a confounder. Eighth, follow-up duration ranged from 8 months to >90 months across included trials; pooling hazard ratios assumes proportional hazards and between-trial exchangeability of the time-averaged treatment effect, which we cannot fully verify at study level. Ninth, we did not undertake an individual patient data (IPD) meta-analysis. IPD-NMA would enable HPV-stratified comparisons but requires data-sharing agreements with each trial sponsor that fell outside the scope and resources of this project; our aggregate-data approach is methodologically valid per PRISMA-NMA guidance. Tenth, our network compared radiosensitiser but did not include altered-fractionation-only arms (without a radiosensitiser); we therefore cannot formally claim cisplatin-based strategies outperform radiation-intensification alone—for that comparison, the MARCH meta-analysis remains the primary evidence base. Eleventh, heterogeneity in the primary network was moderate (I^2^ = 48.9%), reflecting the clinical and methodological diversity of trials conducted across four decades of practice with evolving radiotherapy techniques and patient populations; this is consistent with heterogeneity typically observed in clinical network meta-analyses of this scope. Twelfth, meta-regression analyses in this network are underpowered given the modest number of trials contributing to each covariate, and non-significant p-values for effect-modifier tests (for example laryngeal-cancer proportion and treatment setting) should not be interpreted as evidence that these covariates are unrelated to treatment effect; failure to detect effect modification here may reflect limited statistical power rather than true absence of heterogeneity across subgroups. Thirteenth, most treatment-pair edges in the network contained fewer than ten contributing trials, so the comparison-adjusted funnel plot and Egger's regression test for small-study effects are labelled and interpreted as exploratory only, and cannot be regarded as robust tests for or against publication bias. Fourteenth, although the global design-by-treatment interaction test and local node-splitting analyses did not detect statistically significant inconsistency, these tests are themselves often underpowered in networks of this size and structure; the absence of detected inconsistency should not be equated with formal proof that the assumptions of transitivity and consistency hold uniformly across every edge of the network. Fifteenth, race and ethnicity data were not consistently reported in the included trials, which limits our ability to comment on the representativeness of the study population and on differential radiosensitiser effects across racial or ethnic groups; the included trials span four decades and multiple continents, but predominantly North American, European, and Asian centres, and specific under-represented racial and ethnic groups may be inadequately reflected. Race and ethnicity are sociocultural constructs rather than biological traits, and any signals interpreted through this lens must therefore consider the roles of socioeconomic status, healthcare-access disparities, insurance coverage, geographic differences in radiotherapy technology availability, and other structural drivers as plausible unmeasured confounders. Sixteenth, disability status was not systematically reported in the included trials—most trials used performance-status measures (Karnofsky Performance Status (KPS) or Eastern Cooperative Oncology Group (ECOG) score) as broad eligibility filters rather than as detailed disability-disaggregated data, which means our findings may under-represent people with disabilities and we cannot assess whether treatment effects differ within this population; future trials should adopt validated measures of functional status and disability at baseline and follow-up, and meta-analyses should encourage disability-disaggregated reporting.

This network meta-analysis provides a comprehensive assessment of the radiosensitiser landscape in HNSCC, establishing cisplatin-based regimens as standard while supporting weekly scheduling as an at-least-non-inferior, often preferable alternative to three-weekly dosing. These findings offer a foundation for personalised treatment selection and future trial design.

## Contributors

SMP (Sreeram MP) and PD (Prajwal Dange) contributed equally as co-first authors. SMP conceptualised the study, curated data, performed formal analysis, conducted the investigation, developed the methodology, and drafted, reviewed and edited the manuscript. PD conceptualised the study, curated data, performed formal analysis, conducted the investigation, developed the methodology, contributed to the software implementation, and drafted, reviewed and edited the manuscript. KNR (Karthik N Rao) contributed to data curation, investigation, and reviewed and edited the manuscript. KJH (Kevin J Harrington) provided supervision and validation, and reviewed and edited the manuscript. JBV (Jan B Vermorken) provided supervision and validation, and reviewed and edited the manuscript. AAF (Arlene A Forastiere) provided supervision and validation, and reviewed and edited the manuscript. WMM (William M Mendenhall) provided supervision and validation, and reviewed and edited the manuscript. AF (Alfio Ferlito) conceptualised the study, provided overall supervision and project administration, and reviewed and edited the manuscript. SMP and PD have directly accessed and verified the underlying data, and both had full access to all data in the study and shared final responsibility for the decision to submit the manuscript for publication. All authors read and approved the final version of the manuscript. The International Head and Neck Scientific Group (IHNSG), listed as a collective author on the byline, provided expert peer review of the study protocol, treatment-node taxonomy, and interpretation of the network estimates, and its member roster was drawn on for topic expertise; all IHNSG members named in the accompanying supplementary consortium list have reviewed and approved the final version of the manuscript. AF (Alfio Ferlito), co-founder of the IHNSG, coordinated the consortium's input on behalf of its members.

## Data sharing statement

All data used in this network meta-analysis were extracted from previously published randomised controlled trials and are presented in the Supplementary Material, including study-level hazard ratios, standard errors, treatment-arm definitions, and study characteristics. The complete extracted dataset (CSV files: nma_data.csv, studies.csv, arms.csv, outcomes.csv, toxicity.csv) and the full R analysis pipeline (twelve scripts covering data preparation, frequentist and Bayesian network meta-analysis, figure and table generation, and sensitivity, subgroup and meta-regression analyses) are openly available in a public GitHub repository at https://github.com/dangeprajwal/RADIANCE-NMA, under an MIT licence, with no access restrictions and without the need for a data-access agreement. No individual patient-level data were collected or analysed in this study; all data are aggregate summary data from published reports.

## Declaration of interests

SMP declares no competing interests. PD declares no competing interests. KNR declares no competing interests. KJH declares no competing interests. JBV declares no competing interests. AAF declares no competing interests. WMM declares no competing interests. AF declares no competing interests.
