# Supplementary material for: Radiosensitising strategies in locally advanced head and neck cancer: a systematic review and network meta-analysis of randomised controlled trials (RADIANCE-NMA)
Source: eClinicalMedicine. 2026 Oct 26;101:104263. doi: 10.1016/j.eclinm.2026.104263 (PMC13734942; doi:10.1016/j.eclinm.2026.104263)
Supplement: Supplementary file 1 [file mmc1.pdf]

Supplementary Appendix

Radiosensitising strategies in locally advanced head and neck cancer: a systematic review and network meta-analysis of randomised controlled trials (RADIANCE-NMA)

Manuscript reference: eclinm-D-26-01545R4 (R5 revision, September 2026)

eClinicalMedicine — R5 submission

Supplementary Appendix

Radiosensitising Strategies in Locally Advanced Head and Neck Squamous Cell Carcinoma: A Systematic Review and Network Meta-Analysis (RADIANCE-NMA)

eClinicalMedicine

Contents

Search Strategy	5
Search Results Summary	9
Supplementary Figure S1a: Risk of bias traffic light plot	10
Supplementary Figure S1b: Risk of bias — weighted summary bar plot	11
Supplementary Figure S2: Full sensitivity network plot	13
Supplementary Figure S3: Net heat plot for inconsistency assessment	14
Supplementary Figure S4: Sensitivity analysis comparison	15
Supplementary Figure S5: Setting subgroup comparison forest plot	16
Supplementary Figure S6: Adjuvant subgroup forest plot	18
Supplementary Figure S7: Comparison-adjusted funnel plot	20
Supplementary Figure S8: Toxicity heatmap	21
Supplementary Figure S9: Efficacy-toxicity scatter plot	22
Supplementary Figure S10: Direct evidence plot	23
Supplementary Figure S11: Node-splitting results	25
Supplementary Figure S12: Subgroup forest plot	26
Supplementary Figure S13: Subsite bubble plots	29
Supplementary Figure S14: Era bubble plot	30
Supplementary Figure S15: Era-stratified forest plot	31
Supplementary Figure S16: Weekly vs three-weekly cisplatin — direct-evidence forest plot	32
Supplementary Table S1: Exclusion criteria codes and excluded studies with reasons for exclusion	34
Supplementary Table S2: Risk of bias assessment using the Cochrane RoB 2 tool for all included studies	36
Supplementary Table S3: League table of all pairwise comparisons	39
Supplementary Table S4: Comparison of frequentist P-scores and Bayesian SUCRA rankings for all treatment nodes	41
Supplementary Table S5: Treatment setting subgroup analysis	42
Supplementary Table S6: Sensitivity analysis studies	45

Supplementary Table S7: Excluded studies with reasons for exclusion	47
Supplementary Table S8: Node-splitting results for assessment of local inconsistency	48
Supplementary Table S9: Heterogeneity assessment	49
Supplementary Table S10: Toxicity profile by treatment node	50
Supplementary Table S11: Treatment-related deaths	53
Supplementary Table S12: Meta-regression results	54
Supplementary Table S13: Era subgroup analysis	57

Search Strategy

Supplementary Material: Full Search Strategy

RADIANCE-NMA: Radiosensitising Strategies in Locally Advanced Head and Neck Cancer

1. Search Strategy for PubMed/MEDLINE

Database: PubMed/MEDLINE (National Library of Medicine)

Date of search: 26 June 2026

Date range: Inception to 26 June 2026

Language restrictions: None

The search combined three concept domains using Boolean AND:

#1 Head and neck cancer (Population)

("Head and Neck Neoplasms"[MeSH] OR "Carcinoma, Squamous Cell"[MeSH] OR "Mouth Neoplasms"[MeSH] OR "Pharyngeal Neoplasms"[MeSH] OR "Laryngeal Neoplasms"[MeSH] OR "Oropharyngeal Neoplasms"[MeSH] OR "Hypopharyngeal Neoplasms"[MeSH] OR "Nasopharyngeal Neoplasms"[MeSH] OR "head and neck cancer"[tiab] OR "head and neck squamous cell carcinoma"[tiab] OR "HNSCC"[tiab] OR "SCCHN"[tiab] OR "oropharyngeal cancer"[tiab] OR "oropharyngeal carcinoma"[tiab] OR "oral cavity cancer"[tiab] OR "laryngeal cancer"[tiab] OR "laryngeal carcinoma"[tiab] OR "hypopharyngeal cancer"[tiab] OR "pharyngeal cancer"[tiab] OR "nasopharyngeal cancer"[tiab] OR "nasopharyngeal carcinoma"[tiab] OR "tongue cancer"[tiab] OR "tonsil cancer"[tiab] OR "oral cancer"[tiab])

#2 Radiosensitising agents and radiotherapy (Intervention)

("Radiation-Sensitizing Agents"[MeSH] OR "Radiotherapy"[MeSH] OR "Chemoradiotherapy"[MeSH] OR "radiosensitiz*"[tiab] OR "radiosensitis*"[tiab] OR "chemoradiation"[tiab] OR "chemoradiotherapy"[tiab] OR "concurrent chemo*"[tiab] OR "cisplatin"[tiab] OR "carboplatin"[tiab] OR "cetuximab"[tiab] OR "nimotuzumab"[tiab] OR "nimorazole"[tiab] OR "pembrolizumab"[tiab] OR "nivolumab"[tiab] OR "durvalumab"[tiab] OR "avelumab"[tiab] OR "xevinapant"[tiab] OR "5-fluorouracil"[tiab] OR "5-FU"[tiab] OR "paclitaxel"[tiab] OR "docetaxel"[tiab] OR "mitomycin"[tiab] OR "EGFR inhibitor*"[tiab] OR "immune-checkpoint inhibitor*"[tiab] OR "PD-1"[tiab] OR "PD-L1"[tiab] OR "hypoxic cell sensitiz*"[tiab] OR "NBTXR3"[tiab] OR "etanidazole"[tiab] OR "tirapazamine"[tiab] OR

"nanoparticle*"[tiab] OR "lapatinib"[tiab] OR "erlotinib"[tiab] OR "panitumumab"[tiab] OR "zalutumumab"[tiab])

#3 Randomised trial design (Study design)

("Randomised Controlled Trial"[pt] OR "Controlled Clinical Trial"[pt] OR "randomised controlled trial*"[tiab] OR "randomised controlled trial*"[tiab] OR "RCT"[tiab] OR "random allocation"[tiab] OR "clinical trial*"[tiab] OR "phase II*"[tiab] OR "phase III"[tiab] OR "phase 2"[tiab] OR "phase 3"[tiab])

Final search: #1 AND #2 AND #3

Records retrieved 1,391

2. Search Strategy for Embase

Database: Embase (Elsevier)

Date of search: 26 June 2026

Date range: Inception to 26 June 2026

Language restrictions: None

The PubMed strategy was adapted for Embase using Emtree controlled vocabulary:

#1 Head and neck cancer (Population)

'head and neck cancer'/exp OR 'squamous cell carcinoma'/exp OR 'mouth cancer'/exp OR 'pharynx cancer'/exp OR 'larynx cancer'/exp OR 'oropharynx cancer'/exp OR 'hypopharynx cancer'/exp OR 'nasopharynx cancer'/exp OR 'head and neck cancer':ti,ab OR 'head and neck squamous cell carcinoma':ti,ab OR 'HNSCC':ti,ab OR 'SCCHN':ti,ab OR 'oropharyngeal cancer':ti,ab OR 'oropharyngeal carcinoma':ti,ab OR 'oral cavity cancer':ti,ab OR 'laryngeal cancer':ti,ab OR 'laryngeal carcinoma':ti,ab OR 'hypopharyngeal cancer':ti,ab OR 'pharyngeal cancer':ti,ab OR 'nasopharyngeal cancer':ti,ab OR 'nasopharyngeal carcinoma':ti,ab OR 'tongue cancer':ti,ab OR 'tonsil cancer':ti,ab OR 'oral cancer':ti,ab

#2 Radiosensitising agents and radiotherapy (Intervention)

'radiation sensitizing agent'/exp OR 'radiotherapy'/exp OR 'chemoradiation'/exp OR 'radiosensitiz*':ti,ab OR 'radiosensitis*':ti,ab OR 'chemoradiation':ti,ab OR

'chemoradiotherapy':ti,ab OR 'concurrent chemo*':ti,ab OR 'cisplatin':ti,ab OR 'carboplatin':ti,ab OR 'cetuximab':ti,ab OR 'nimotuzumab':ti,ab OR 'nimorazole':ti,ab OR 'pembrolizumab':ti,ab OR 'nivolumab':ti,ab OR 'durvalumab':ti,ab OR 'avelumab':ti,ab OR 'xevinapant':ti,ab OR '5-fluorouracil':ti,ab OR '5-FU':ti,ab OR 'paclitaxel':ti,ab OR 'docetaxel':ti,ab OR 'mitomycin':ti,ab OR 'EGFR inhibitor*':ti,ab OR 'immune-checkpoint inhibitor*':ti,ab OR 'PD-1':ti,ab OR 'PD-L1':ti,ab OR 'hypoxic cell sensitiz*':ti,ab OR 'NBTXR3':ti,ab OR 'etanidazole':ti,ab OR 'tirapazamine':ti,ab OR 'nanoparticle*':ti,ab OR 'lapatinib':ti,ab OR 'erlotinib':ti,ab OR 'panitumumab':ti,ab OR 'zalutumumab':ti,ab

#3 Randomised trial design (Study design)

'randomised controlled trial'/exp OR 'phase 3 clinical trial'/exp OR 'randomised controlled trial*':ti,ab OR 'randomised controlled trial*':ti,ab OR 'RCT':ti,ab OR 'random allocation':ti,ab OR 'clinical trial*':ti,ab OR 'phase II*':ti,ab OR 'phase III':ti,ab OR 'phase 2':ti,ab OR 'phase 3':ti,ab

Final search: #1 AND #2 AND #3

Records retrieved 923

3. Search Strategy for Cochrane CENTRAL

Database: Cochrane Central Register of Controlled Trials (CENTRAL)

Date of search: 26 June 2026

Date range: Inception to 26 June 2026

Language restrictions: None

Limited to: Trials

#1 Population

("head and neck cancer" OR "head and neck squamous cell carcinoma" OR "HNSCC" OR "SCCHN" OR "oropharyngeal" OR "laryngeal" OR "hypopharyngeal" OR "oral cavity" OR "nasopharyngeal" OR "pharyngeal" OR "tongue cancer" OR "tonsil cancer"):ti,ab,kw

#2 Intervention

("radiosensitiz*" OR "chemoradiation" OR "chemoradiotherapy" OR "concurrent chemo*" OR "cisplatin" OR "carboplatin" OR "cetuximab" OR "nimotuzumab" OR "nimorazole" OR "pembrolizumab" OR "nivolumab" OR "immunotherapy" OR "5-fluorouracil" OR "paclitaxel" OR "EGFR" OR "nanoparticle*" OR "etanidazole" OR "tirapazamine" OR "xevinapant"):ti,ab,kw

Final search: #1 AND #2

Records retrieved 418

4. Search Strategy for ClinicalTrials.gov

Date of search: 26 June 2026

Search parameters:

Condition or disease: head and neck cancer OR HNSCC OR squamous cell carcinoma of the head and neck

Intervention/treatment: radiosensitiser OR cisplatin OR cetuximab OR immunotherapy OR nimorazole OR chemoradiotherapy OR pembrolizumab OR nivolumab

Study type: Interventional (Clinical Trial)

Phase: Phase 2, Phase 3

Status: Completed, Active not recruiting, Terminated

Results: With results

Records retrieved: 98

5. Search Results Summary

After removing 1,428 duplicates, 1,402 records underwent title and abstract screening. Of 316 full-text articles assessed for eligibility, 250 were excluded. Snowball searching (backward reference checking) of included studies identified 6 additional studies not captured by the database search. In total, 67 studies met inclusion criteria: 44 in the primary network meta-analysis and 23 in prespecified sensitivity analyses.

Database	Records identified

PubMed/MEDLINE	1,391
Embase	923
Cochrane CENTRAL	418
ClinicalTrials.gov	98
Total	2,830
Backward reference checking	6 additional studies

Supplementary Figure S1a. Risk of bias traffic light plot. Individual risk of bias judgements for each included study across the five Cochrane RoB 2 domains (D1 randomization; D2 deviations from intended interventions; D3 missing outcome data; D4 outcome measurement; D5 selection of reported result). Sensitivity-analysis studies not in the primary NMA are marked with †.

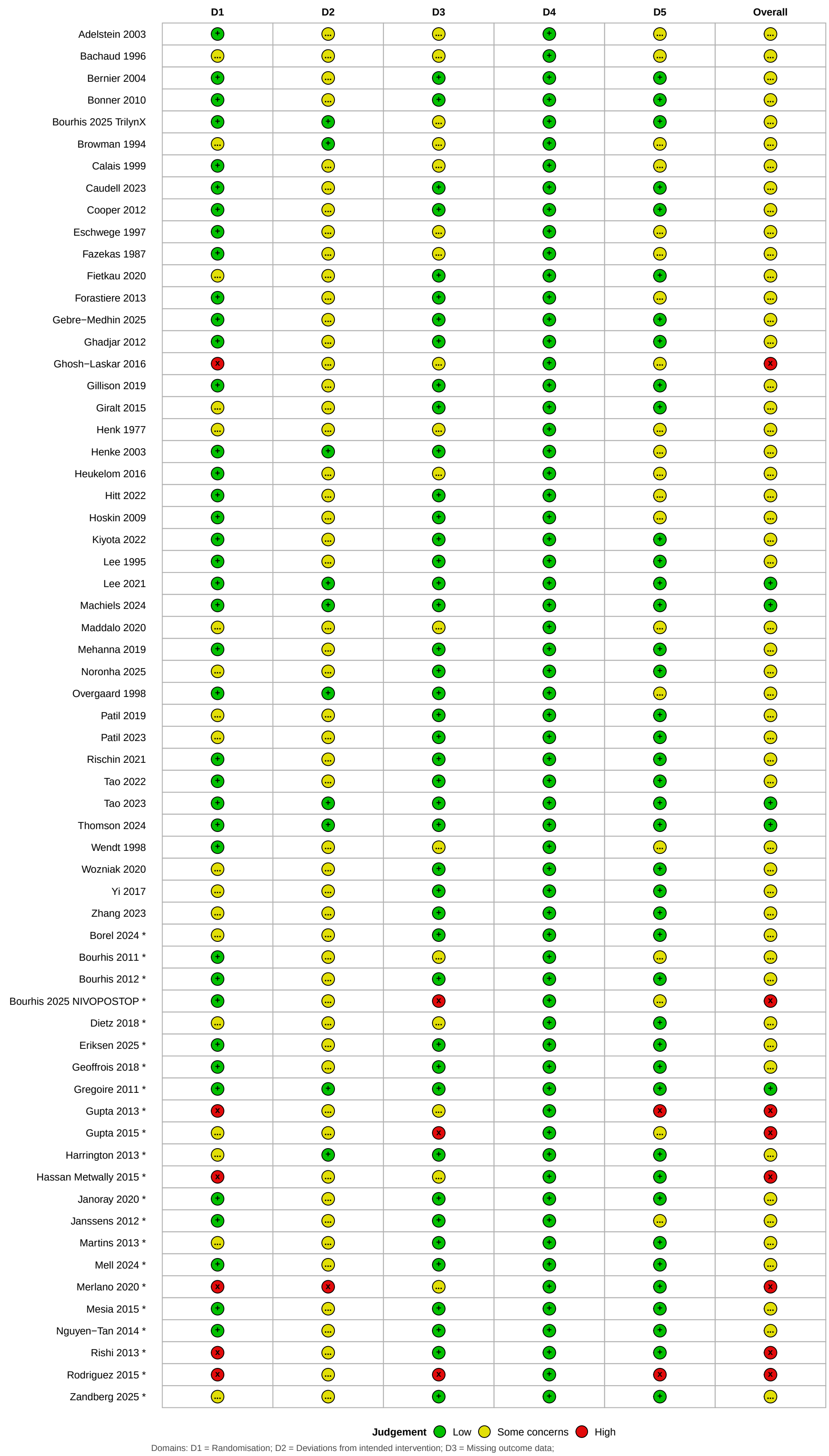

Supplementary Figure S1b. Risk of bias – weighted summary bar plot. Proportion of studies at low, some-concerns, or high risk of bias across the five Cochrane RoB 2 domains, weighted by study sample size.

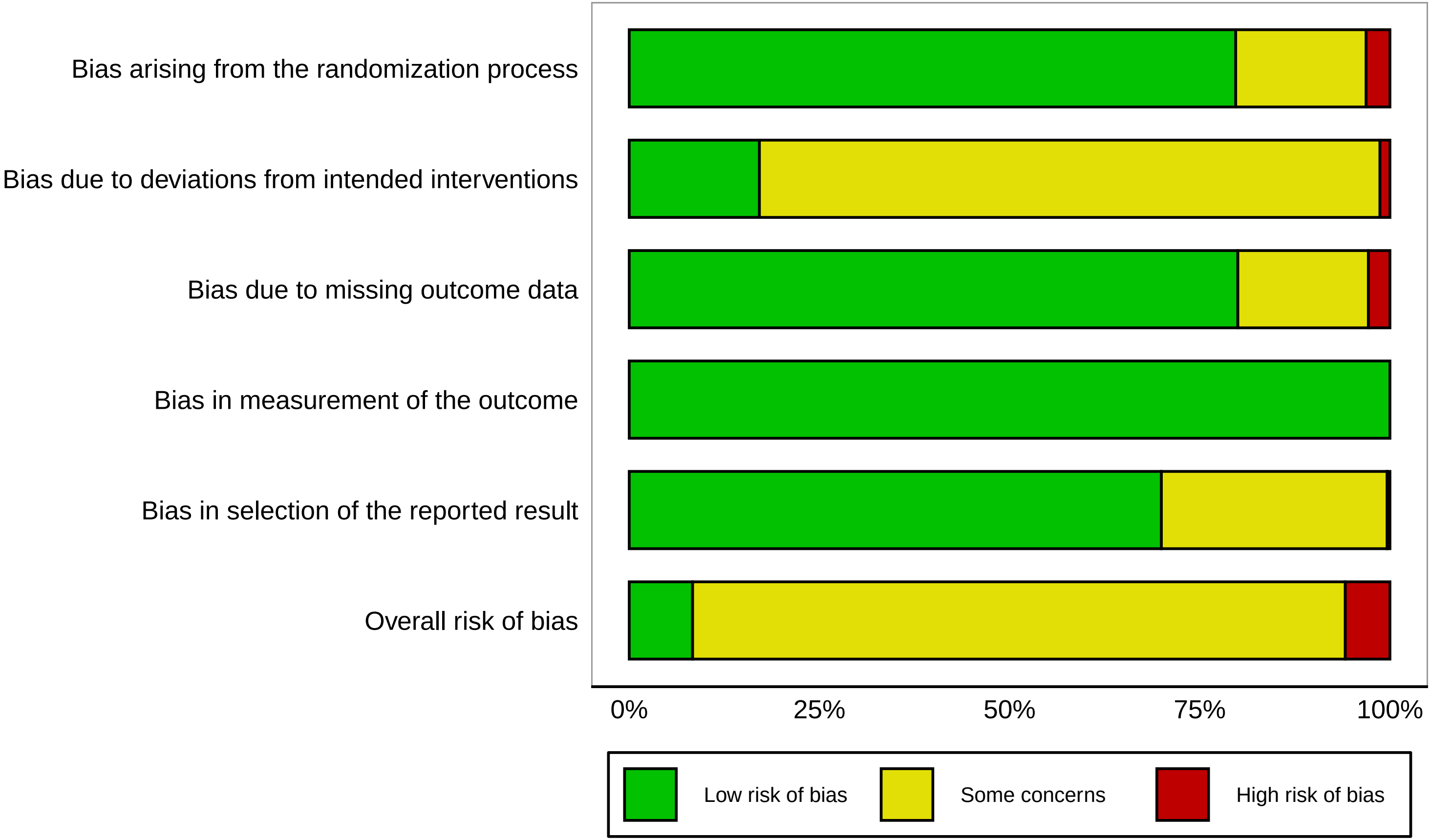
Bias arising from the randomization process
Bias due to deviations from intended interventions
Bias due to missing outcome data
Bias in measurement of the outcome
Bias in selection of the reported result
Overall risk of bias
0%
25%
50%
75%
100%
Low risk of bias
Some concerns
High risk of bias

Supplementary Figure S2. Full sensitivity network plot. Network geometry including all 63 studies (40 main + 23 sensitivity-only) with 45 treatment nodes and 49 direct comparisons. Node size proportional to number of participants; edge width proportional to number of trials.

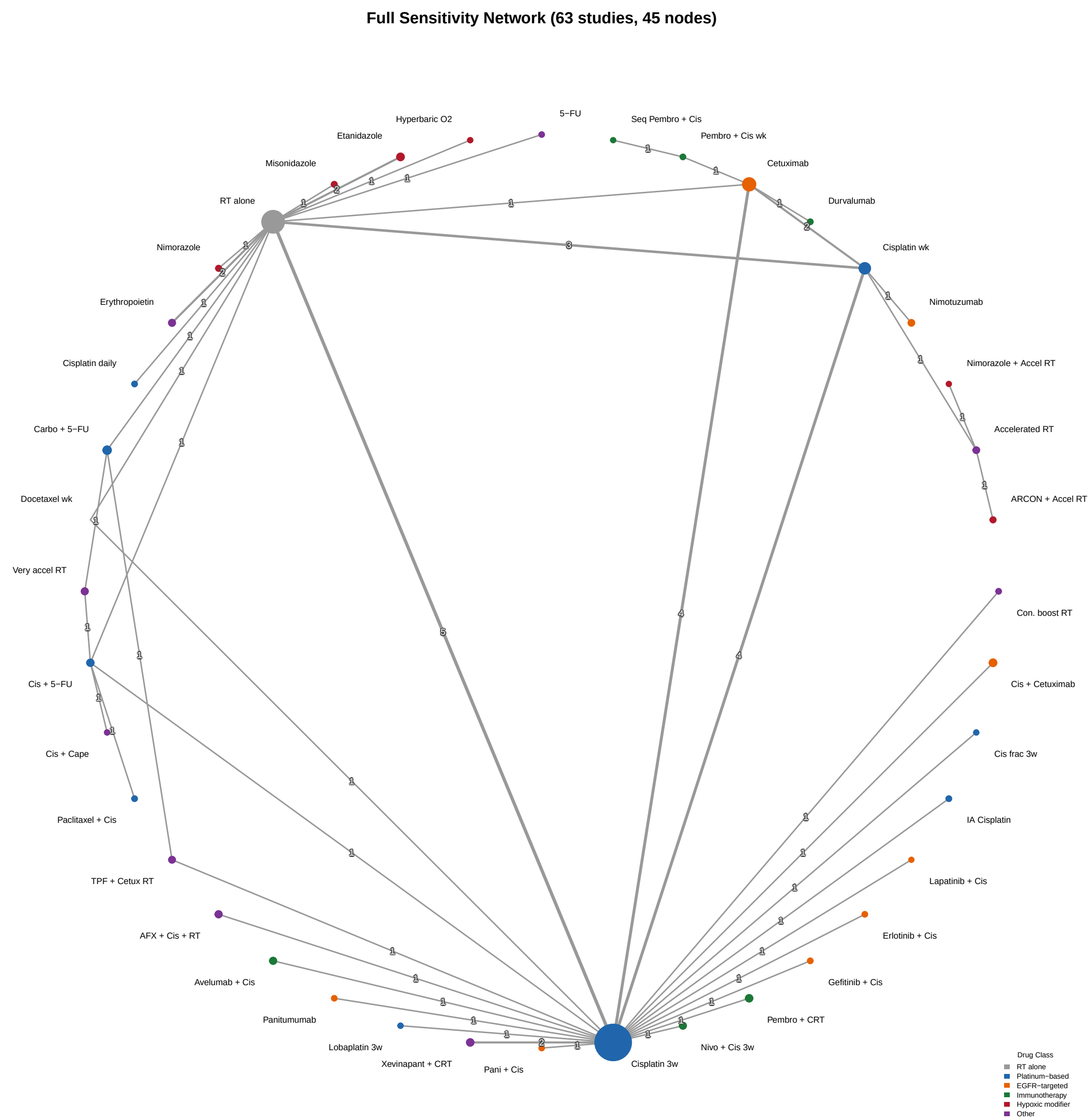

Supplementary Figure S3. Net heat plot for inconsistency assessment. Grey shading indicates areas of low inconsistency; yellow shading indicates potential inconsistency between direct and indirect evidence.

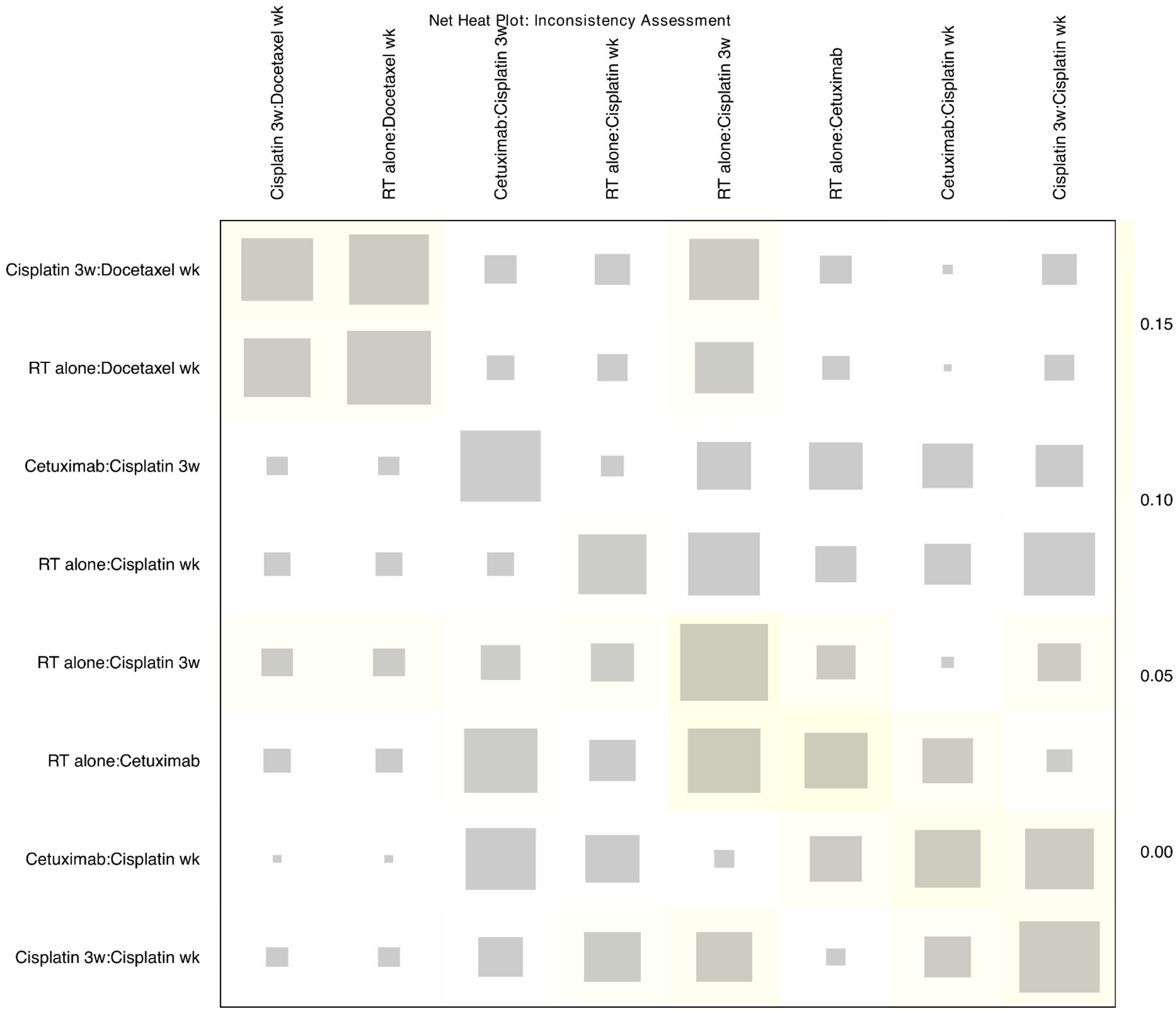

Supplementary Figure S4. Sensitivity analysis comparison. Forest plot comparing hazard ratios for overall survival from the primary NMA (39 studies) with the expanded sensitivity NMA (63 studies), for every treatment node present in both networks. Point estimates and 95% confidence intervals are shown; the vertical dashed line at HR=1 represents no difference vs radiotherapy alone.

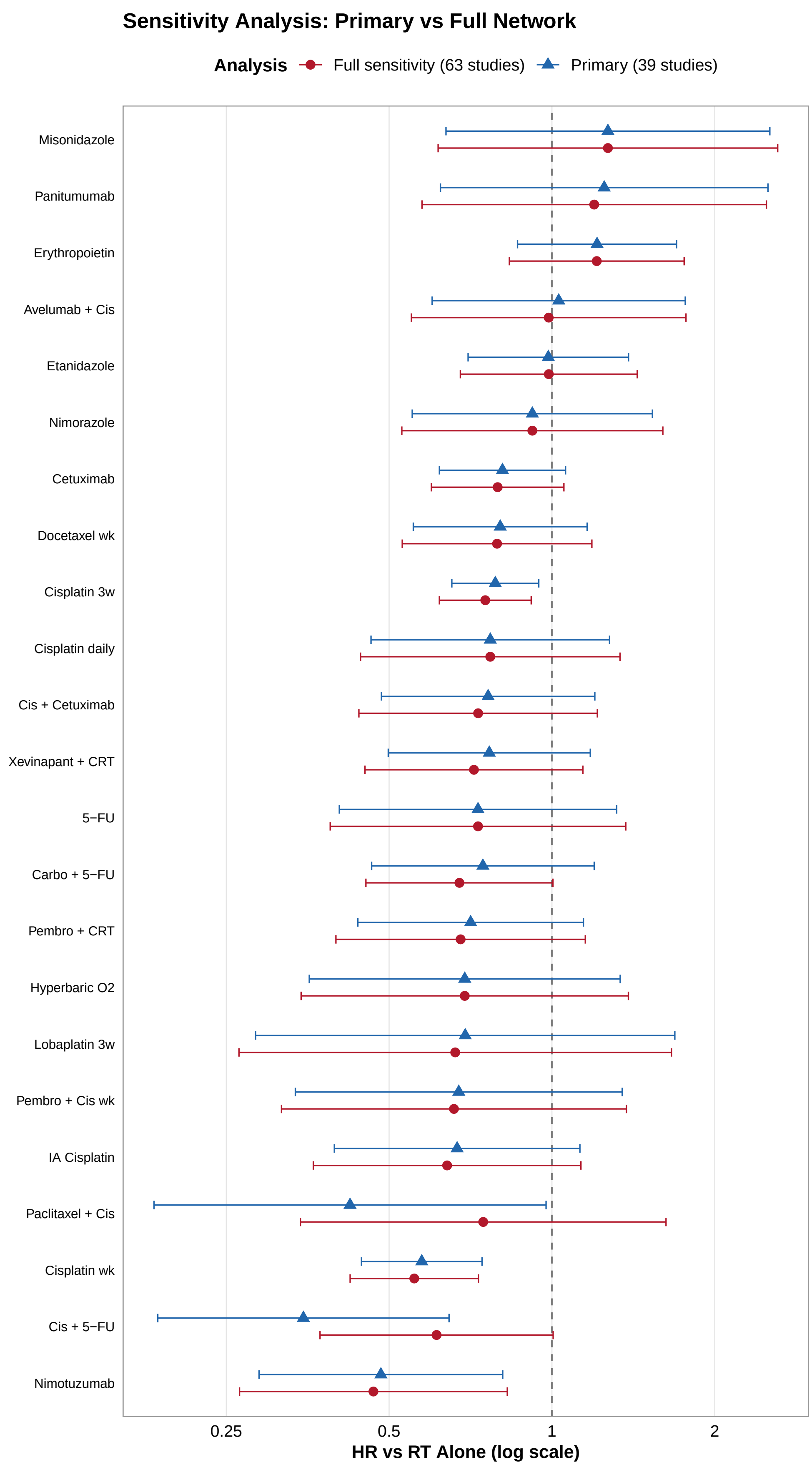

Supplementary Figure S5. Setting subgroup comparison. Forest plot comparing hazard ratios from the all-setting primary NMA versus the definitive-setting-only subgroup analysis. Concordance between the two supports pooling across treatment settings.

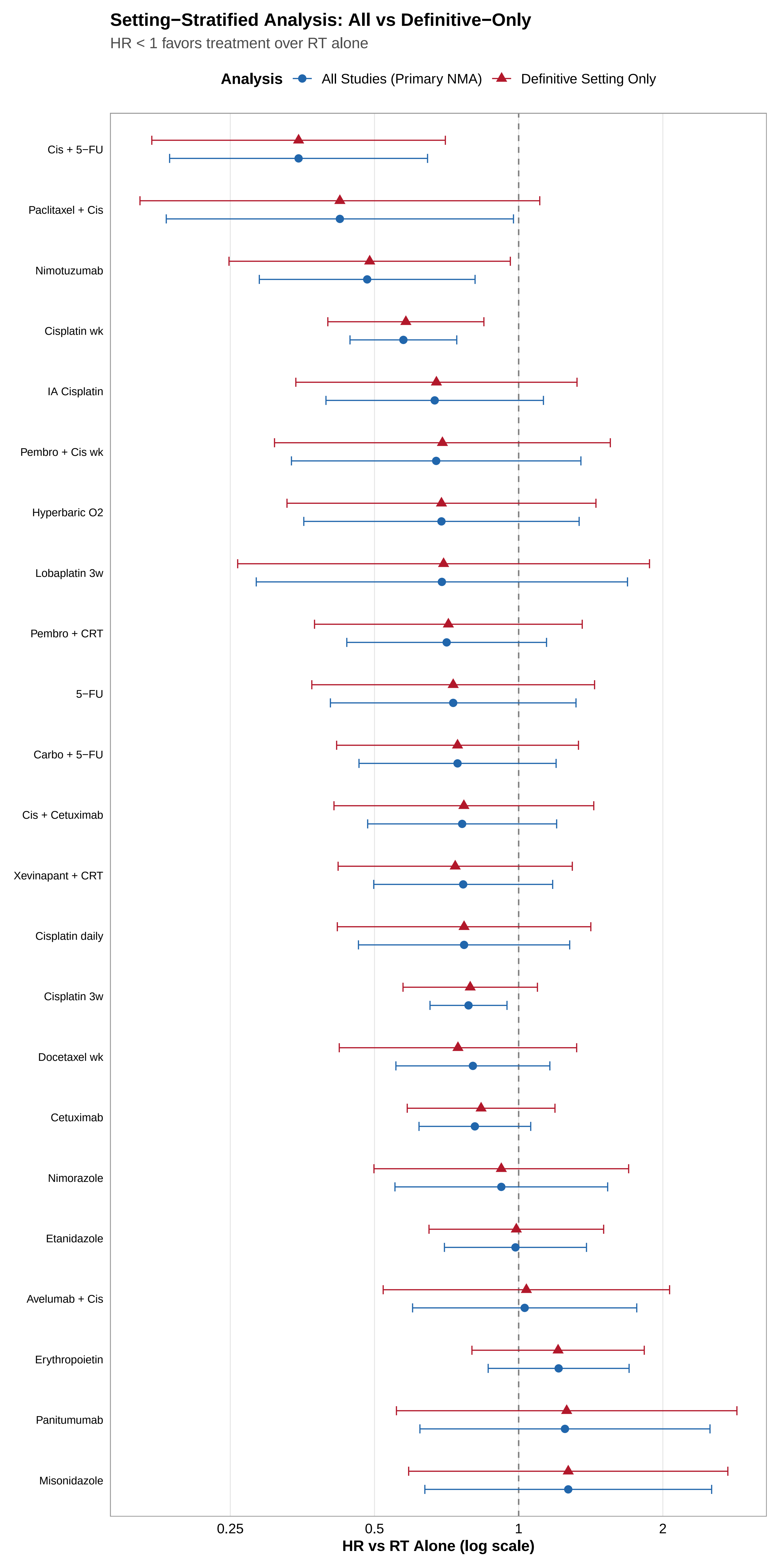
Setting−Stratified Analysis: All vs Definitive−Only
HR < 1 favors treatment over RT alone
Analysis
All Studies (Primary NMA)
Definitive Setting Only
Cis + 5−FU
Paclitaxel + Cis
Nimotuzumab
Cisplatin wk
IA Cisplatin
Pembro + Cis wk
Hyperbaric O2
Lobaplatin 3w
Pembro + CRT
5−FU
Carbo + 5−FU
Cis + Cetuximab
Xevinapant + CRT
Cisplatin daily
Cisplatin 3w
Docetaxel wk
Cetuximab
Nimorazole
Etanidazole
Avelumab + Cis
Erythropoietin
Panitumumab
Misonidazole
0.25
0.5
1
2
HR vs RT Alone (log scale)

Supplementary Figure S6. Adjuvant subgroup forest plot. Forest plot of treatment effects restricted to studies conducted in the adjuvant (postoperative) setting.

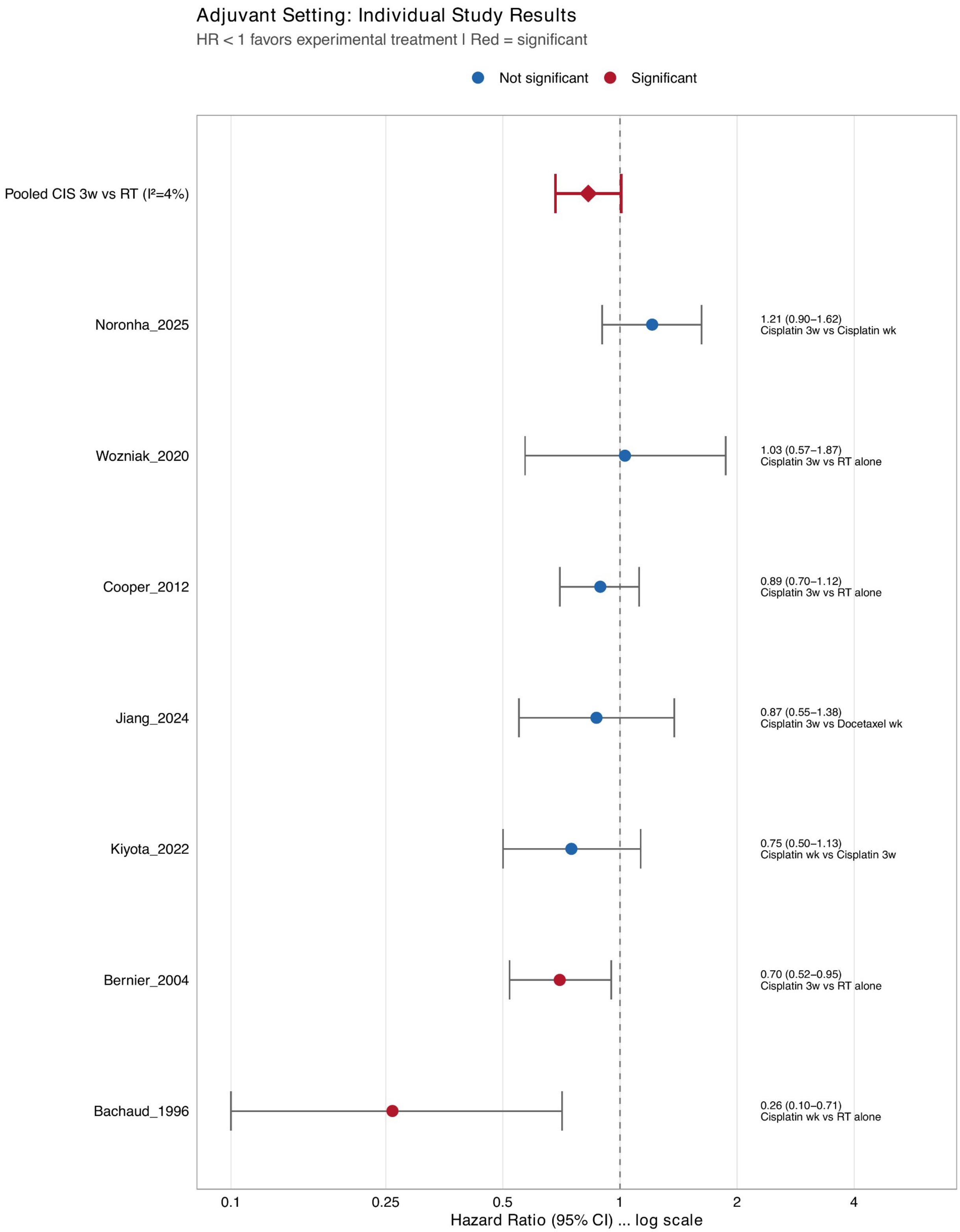

Supplementary Figure S7. Comparison-adjusted funnel plot. Assessment of small-study effects and publication bias across all direct comparisons in the network.

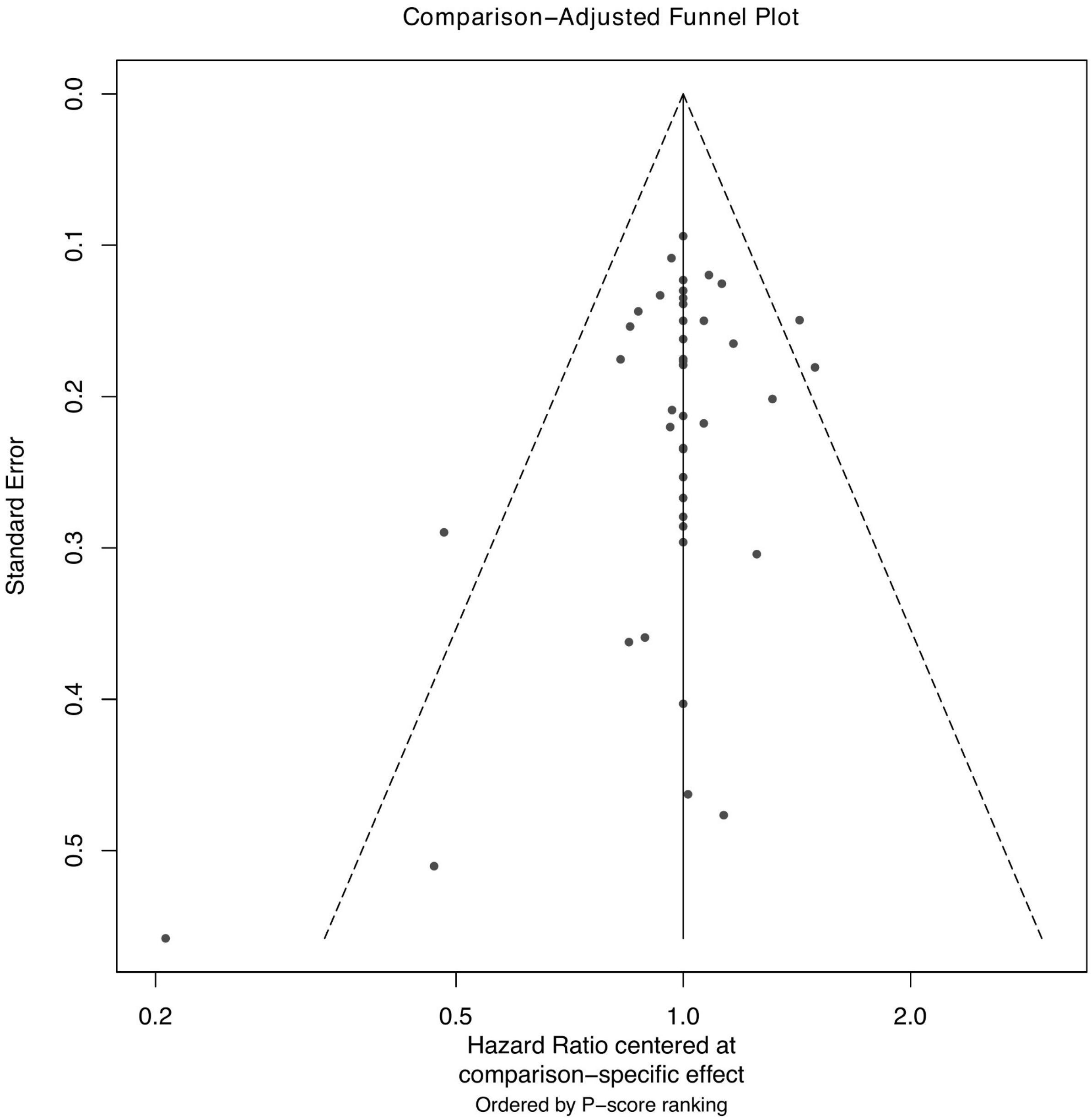

Supplementary Figure S8. Toxicity heatmap. Rates of grade 3+ adverse events by treatment node across major toxicity categories (mucositis, dysphagia, dermatitis, neutropenia, nephrotoxicity, nausea/vomiting).

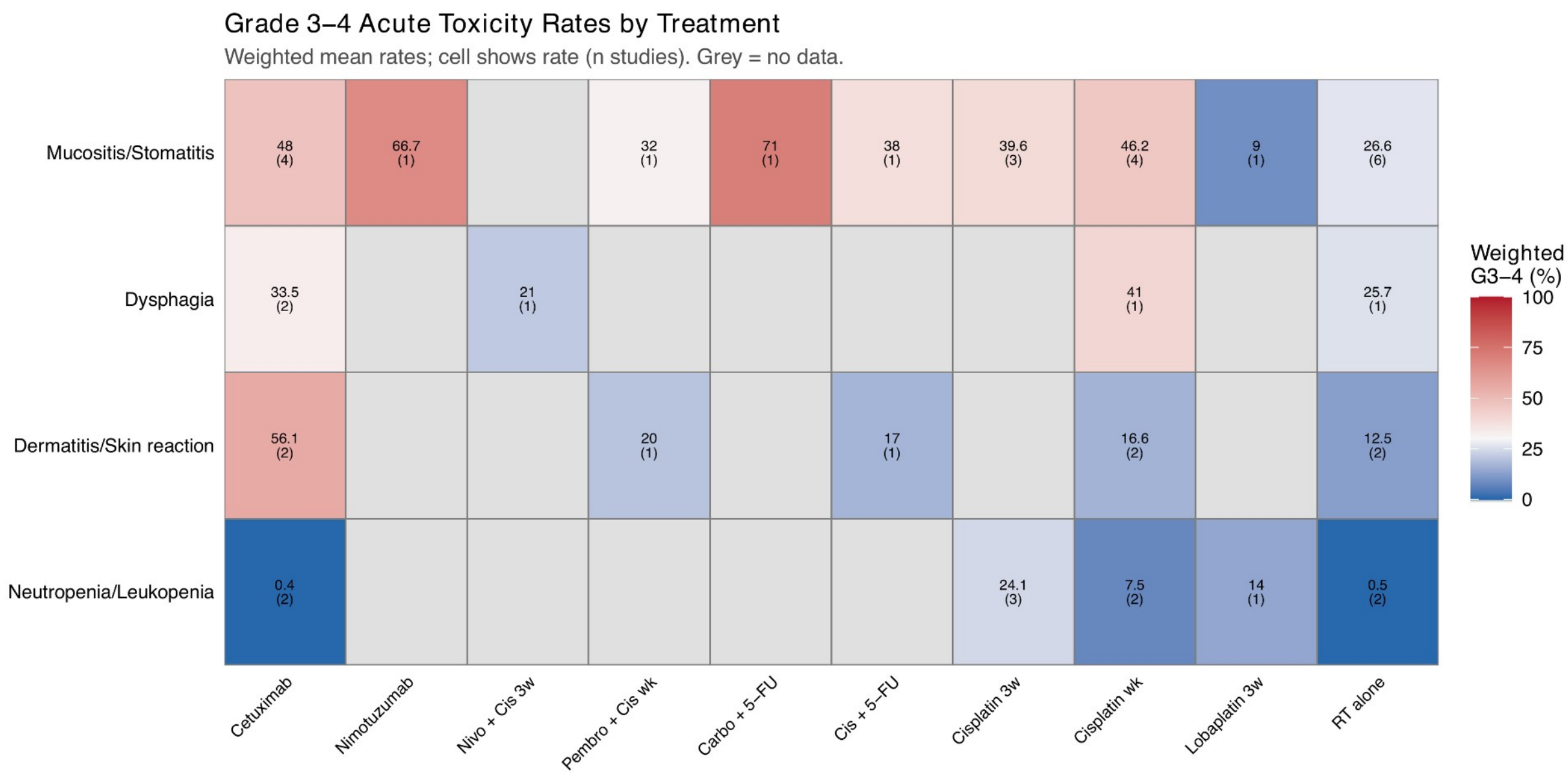

Supplementary Figure S9. Efficacy-toxicity scatter plot. Relationship between overall survival P-score (efficacy ranking) and weighted mean grade 3+ toxicity rate for each treatment node.

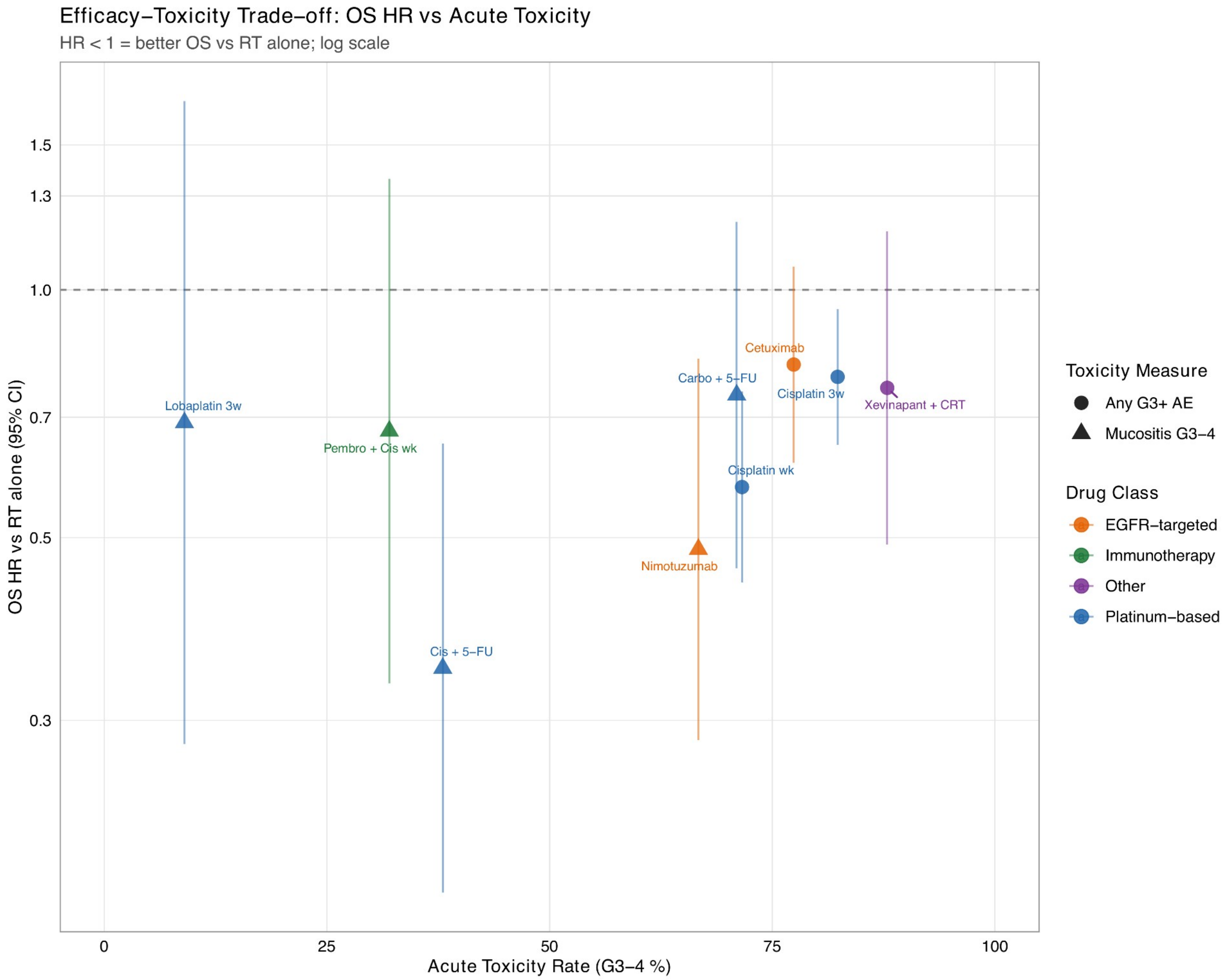

Supplementary Figure S10. Direct evidence plot. Contribution of direct evidence to each network estimate, showing the proportion of information derived from direct comparisons versus indirect evidence.

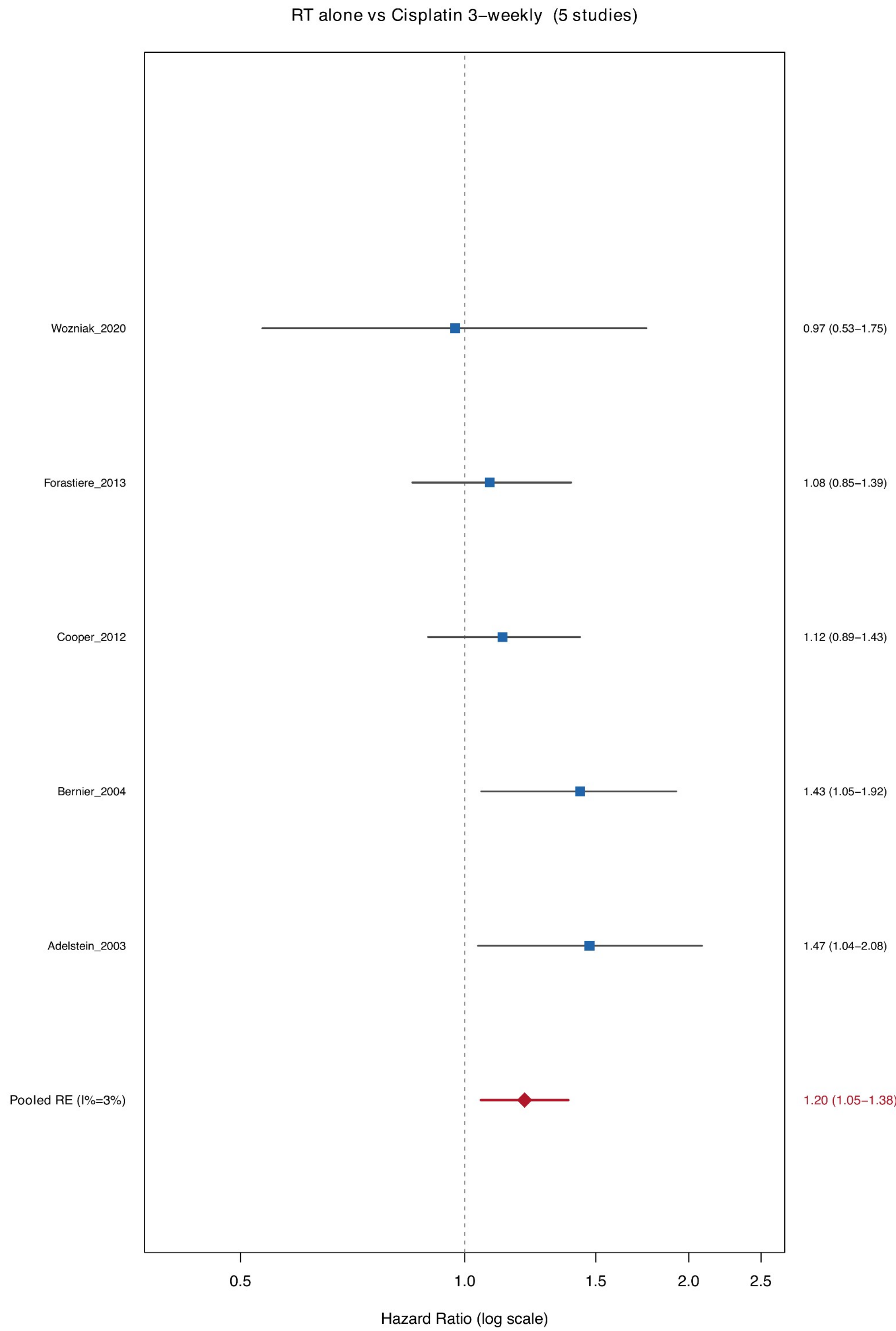

Supplementary Figure S11. Node-splitting results. Comparison of direct and indirect estimates for each contrast where both types of evidence are available, with test for inconsistency.

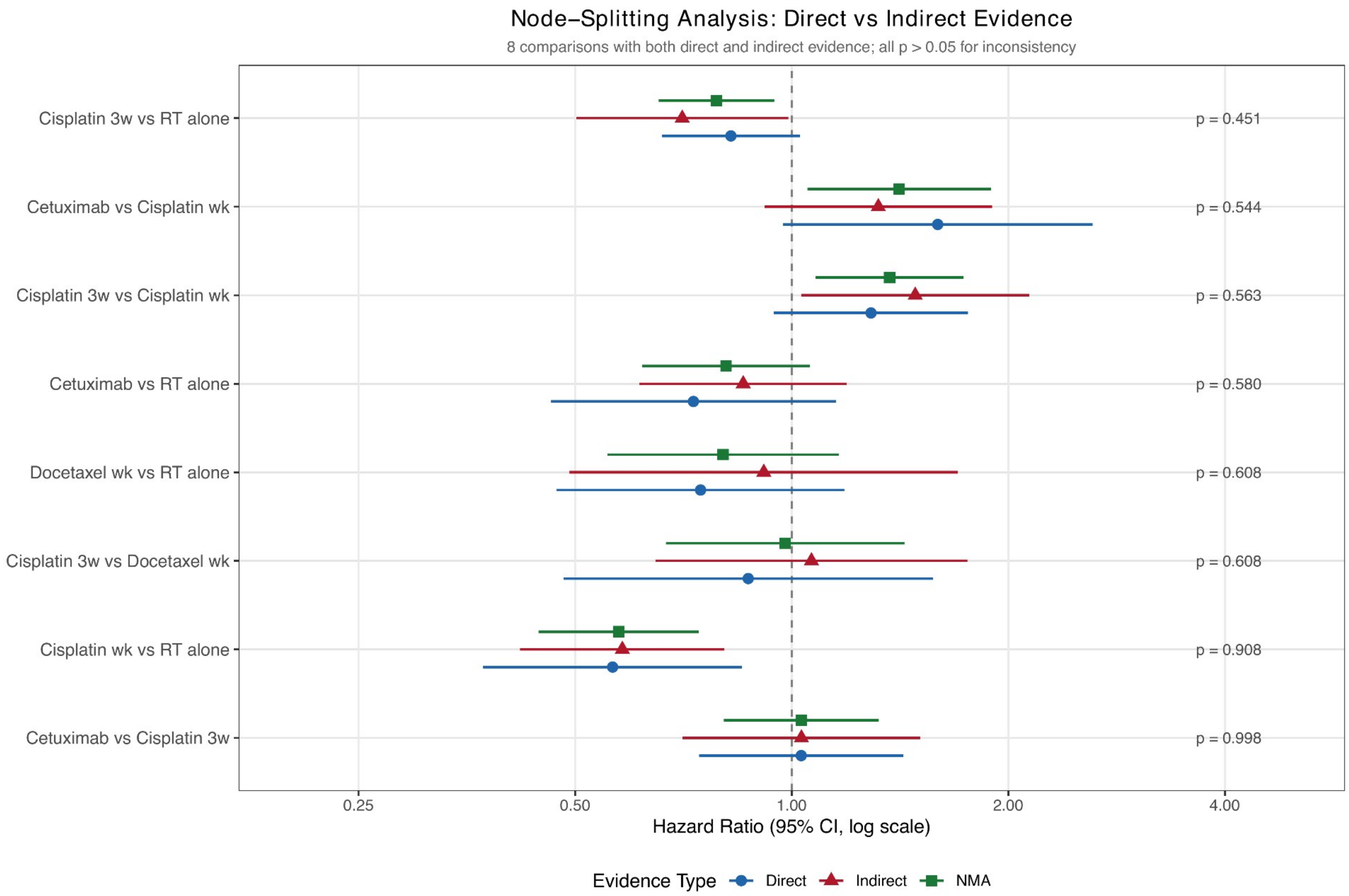

Supplementary Figure S12. Subgroup forest plot. Treatment effects stratified by predefined subgroups including treatment setting, HPV status, and primary tumour subsite.

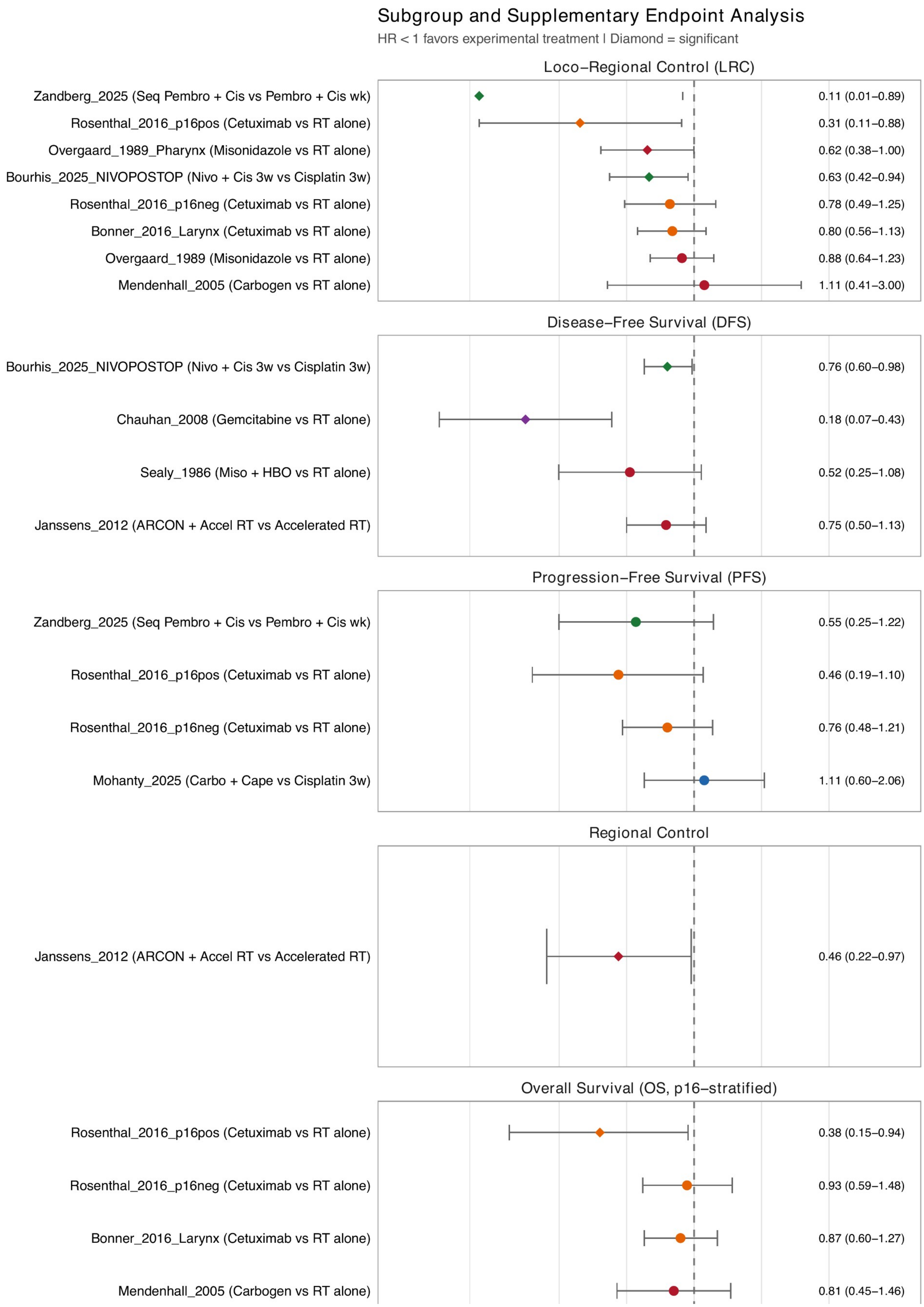

Subgroup and Supplementary Endpoint Analysis
HR < 1 favors experimental treatment | Diamond = significant
Loco–Regional Control (LRC)
Zandberg_2025 (Seq Pembro + Cis vs Pembro + Cis wk)
0.11 (0.01–0.89)
Rosenthal_2016_p16pos (Cetuximab vs RT alone)
0.31 (0.11–0.88)
Overgaard_1989_Pharynx (Misonidazole vs RT alone)
0.62 (0.38–1.00)
Bourhis_2025_NIVOPOSTOP (Nivo + Cis 3w vs Cisplatin 3w)
0.63 (0.42–0.94)
Rosenthal_2016_p16neg (Cetuximab vs RT alone)
0.78 (0.49–1.25)
Bonner_2016_Larynx (Cetuximab vs RT alone)
0.80 (0.56–1.13)
Overgaard_1989 (Misonidazole vs RT alone)
0.88 (0.64–1.23)
Mendenhall_2005 (Carbogen vs RT alone)
1.11 (0.41–3.00)
Disease–Free Survival (DFS)
Bourhis_2025_NIVOPOSTOP (Nivo + Cis 3w vs Cisplatin 3w)
0.76 (0.60–0.98)
Chauhan_2008 (Gemcitabine vs RT alone)
0.18 (0.07–0.43)
Sealy_1986 (Miso + HBO vs RT alone)
0.52 (0.25–1.08)
Janssens_2012 (ARCON + Accel RT vs Accelerated RT)
0.75 (0.50–1.13)
Progression–Free Survival (PFS)
Zandberg_2025 (Seq Pembro + Cis vs Pembro + Cis wk)
0.55 (0.25–1.22)
Rosenthal_2016_p16pos (Cetuximab vs RT alone)
0.46 (0.19–1.10)
Rosenthal_2016_p16neg (Cetuximab vs RT alone)
0.76 (0.48–1.21)
Mohanty_2025 (Carbo + Cape vs Cisplatin 3w)
1.11 (0.60–2.06)
Regional Control
Janssens_2012 (ARCON + Accel RT vs Accelerated RT)
0.46 (0.22–0.97)
Overall Survival (OS, p16–stratified)
Rosenthal_2016_p16pos (Cetuximab vs RT alone)
0.38 (0.15–0.94)
Rosenthal_2016_p16neg (Cetuximab vs RT alone)
0.93 (0.59–1.48)
Bonner_2016_Larynx (Cetuximab vs RT alone)
0.87 (0.60–1.27)
Mendenhall_2005 (Carbogen vs RT alone)
0.81 (0.45–1.46)

Supplementary Figure S13. Subsite bubble plots. Bubble plots showing the relationship between (A) oropharyngeal, (B) HPV-positive, and (C) laryngeal proportions and treatment effect (log hazard ratio) across studies. Bubble size proportional to study weight; colour indicates drug class; shape indicates treatment setting. Slope, R^2 and p from meta-regression are inset on each panel.

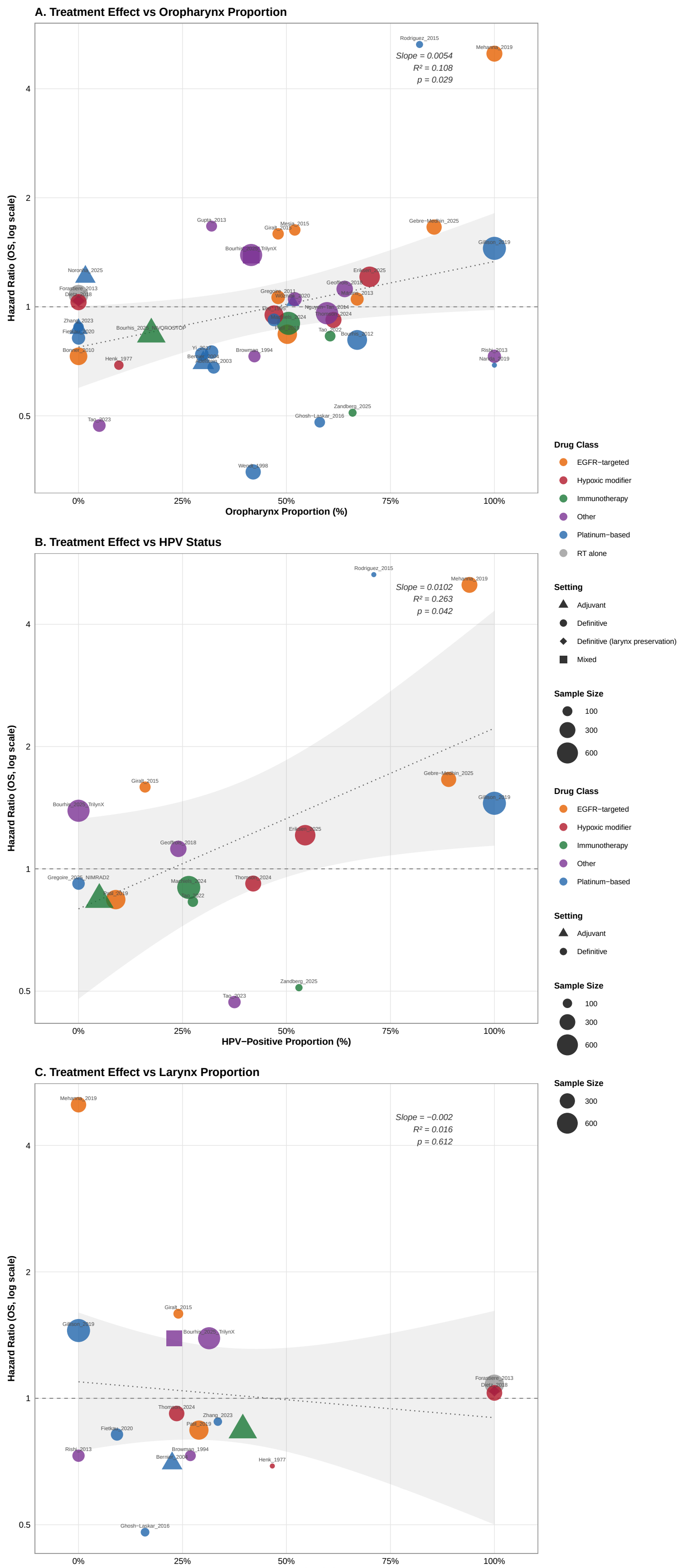

Supplementary Figure S14. Era bubble plot. Bubble plot showing the relationship between median enrolment year and treatment effect (log hazard ratio), with bubble size proportional to study weight.

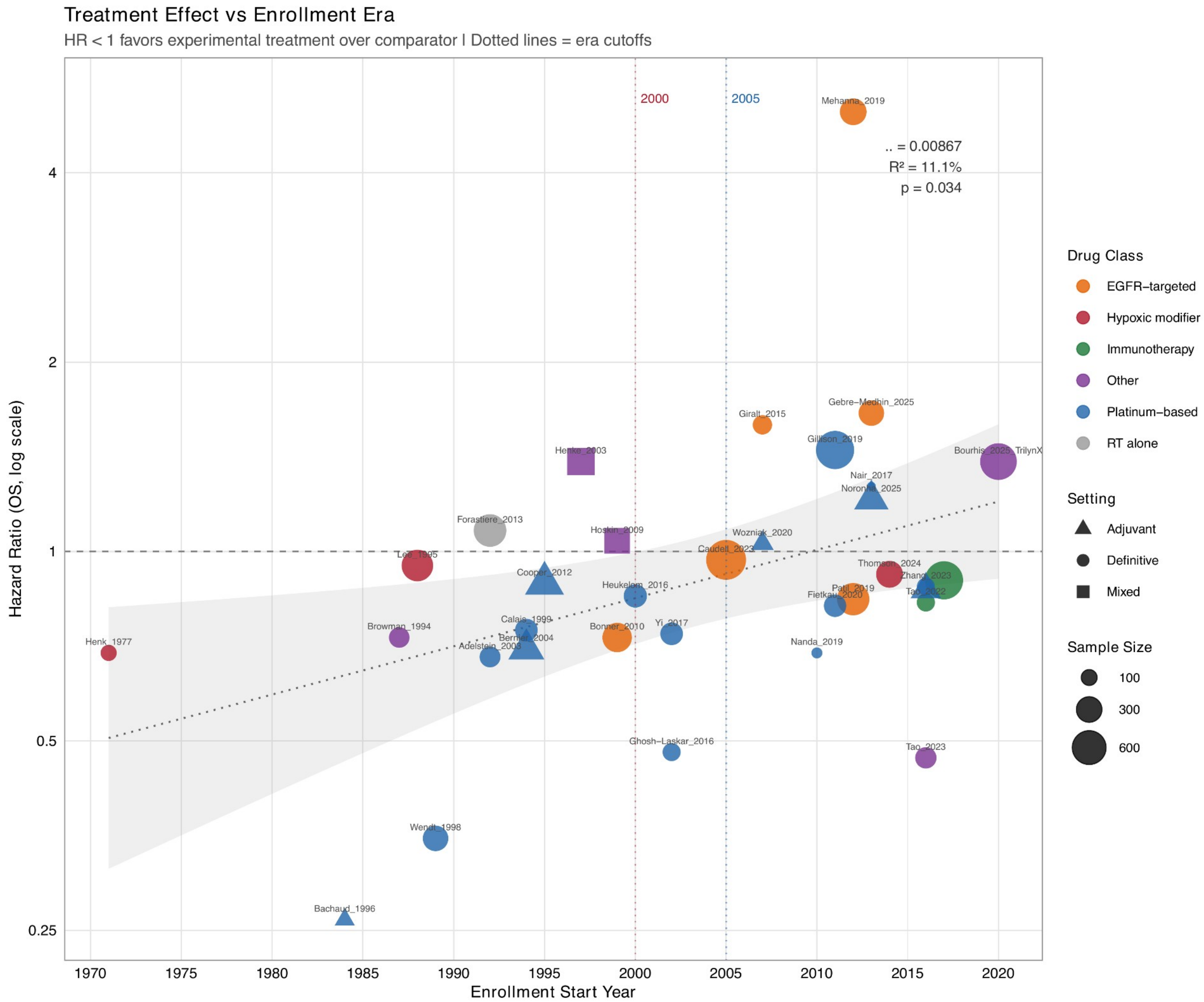

Supplementary Figure S15. Era-stratified forest plot. Treatment effects vs radiotherapy alone stratified by enrolment era, using two cut-points (2000 and 2005). Analysis is restricted to treatment nodes contributing in both eras, showing attenuation of chemotherapy benefit in the modern intensity-modulated radiotherapy (IMRT) era.

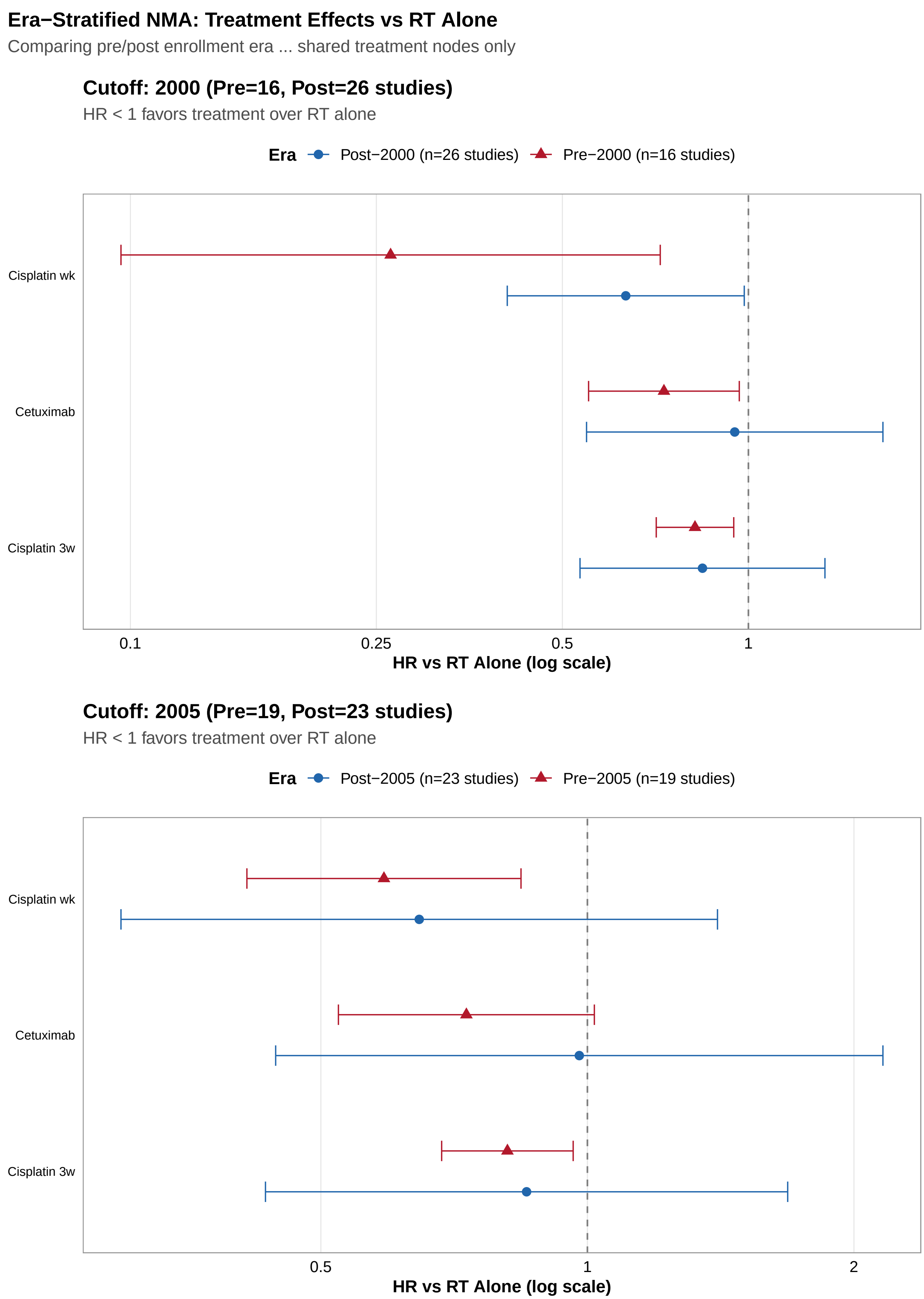

Supplementary Figure S16. Weekly versus three-weekly cisplatin — direct-evidence forest plot. Head-to-head randomised trials comparing weekly and three-weekly cisplatin schedules with concurrent radiotherapy: Kiyota 2022 (JCOG1008), Noronha 2025 (long-term update), Nair 2017, and Nanda 2019. Study-level HRs with 95% CIs and sample sizes are shown; the diamond at the bottom is the random-effects pooled estimate. HR < 1 favours weekly cisplatin; HR > 1 favours three-weekly cisplatin.

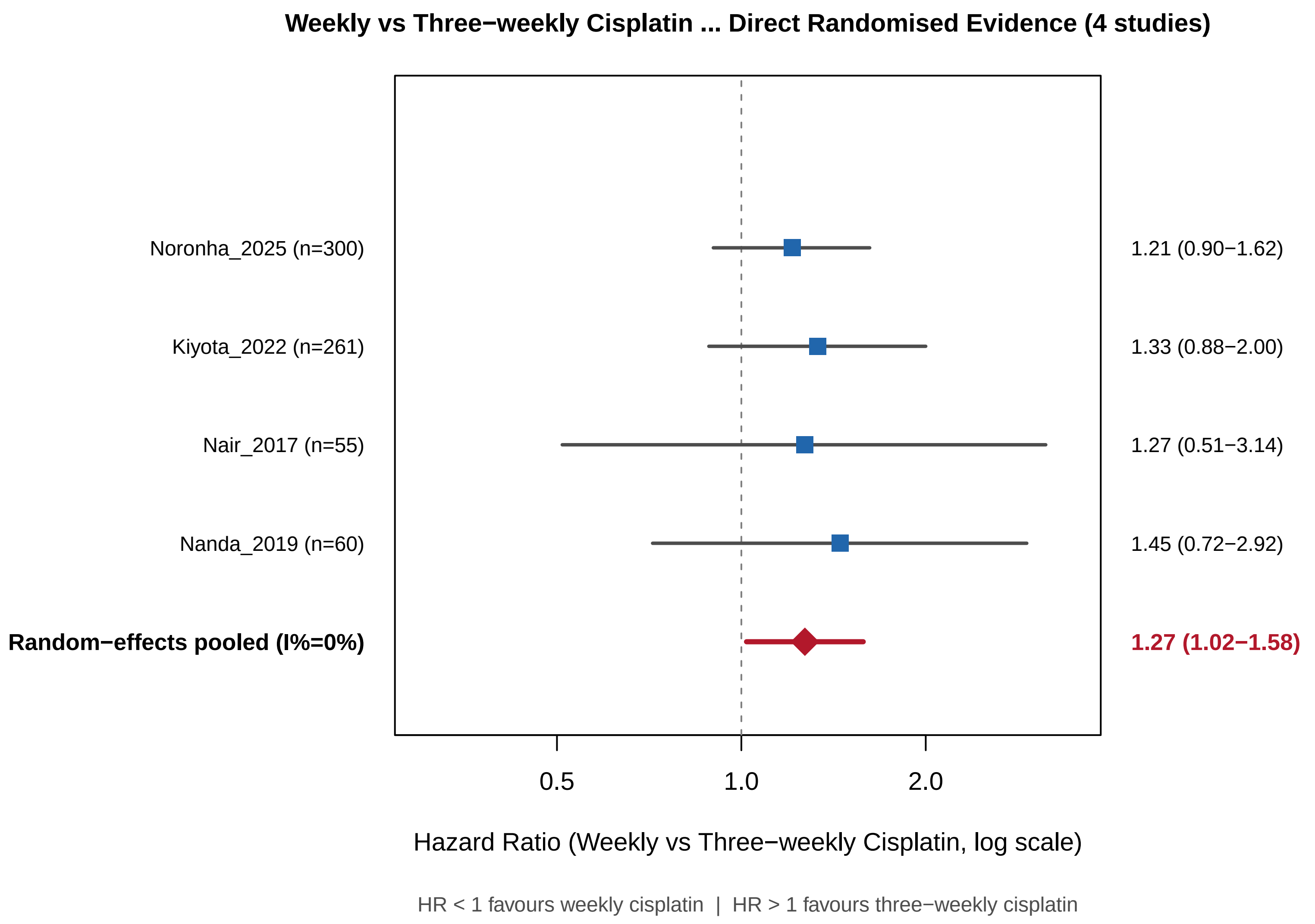
Weekly vs Three−weekly Cisplatin ... Direct Randomised Evidence (4 studies)
Noronha_2025 (n=300)
1.21 (0.90−1.62)
Kiyota_2022 (n=261)
1.33 (0.88−2.00)
Nair_2017 (n=55)
1.27 (0.51−3.14)
Nanda_2019 (n=60)
1.45 (0.72−2.92)
Random−effects pooled (I%=0%)
1.27 (1.02−1.58)
0.5
1.0
2.0
Hazard Ratio (Weekly vs Three−weekly Cisplatin, log scale)
HR < 1 favours weekly cisplatin | HR > 1 favours three−weekly cisplatin

Supplementary Table S1. Exclusion criteria codes and excluded studies with reasons for exclusion.

Sheet: Exclusion Codes

Code	Reason
E14	No OS data
E15	No HR reported/estimable
E20	Induction comparison only
E22	Both arms have systemic therapy (unless both map to existing NMA nodes)
E26	Treatment modality comparison (RT vs. surgery)
E27	Protocol/safety only (no efficacy data)
E28	Phase IIa study (single-arm; Phase IIb randomized comparative may be included)
E29	HR not estimable from available data
E31	Secondary analysis of included trial
E33	De-escalation trial with non-standard RT doses
E24	Asymmetric induction design

Sheet: Excluded Studies

Study	Year	Exclusion Code(s)	Brief Reason
Ameri 2022	2022	E14	Toxicity-only report
Bakowski 1978	1978	E14	Razoxane, no OS data
Bartelink 2002	2002	E22, E28	Both arms cisplatin-based
Brizel 1998	1998	E22	Hyperfractionated RT control; neither arm maps to existing NMA node
Budach 2015	2015	E22	RT doses differ between arms
Chassagne 1992	1992	E14	Interim report only
Chauhan 2008	2008	E15	DFS only, no OS
de Leeuw 2024	2024	E22	Both arms cisplatin-based
Halim 2012	2012	E22, E15	Gemcitabine vs paclitaxel; orphan nodes, no HR, no connectivity
Harari 2014	2014	E22, E28, E15	Both arms cetuximab; Phase II non-comparative
Huilgol 1996	1996	E28, E14, E15	Sanazole Phase I/II; n=18, dose-finding only
Lefebvre 2013	2013	E31	TREMPLIN duplicate report; superseded by Janoray 2020
Ma 2025	2025	E22, E33	De-escalation trial; 30Gy vs 60Gy RT, HPV+ only
Mendenhall 2005	2005	E22	Carbogen; hyperfractionated RT control not mappable
Mohanty 2025	2025	E22, E28, E15	CARCAP-HN; no OS HR, PFS in subgroup analysis
Overgaard 1989	1989	E15	Misonidazole; LRC only, no OS data
Overgaard 2003	2003	E22	RT fractionation comparison; nimorazole symmetric in both arms
Palma 2025	2025	E26	ORATOR2; RT vs surgery, no drug contrast
Panis 1984	1984	E15, E28	Misonidazole; n=52, no HR
Quon 2011	2011	E15	Low-dose cisplatin (20mg/m2), no HR
Rosenthal 2016	2016	E31	HPV subgroup analysis of Bonner 2010

Study	Year	Exclusion Code(s)	Brief Reason
Sealy 1986	1986	E15	Misonidazole + HBO; no HR, confounded RT doses
Tao 2020	2020	E27	GORTEC 2017-01 REACH; safety phase only
Ullal 2006	2006	E28, E14	Sanazole Phase II; n=46, no OS
Van den Bogaert 1995	1995	E22, E15	RT fractionation + misonidazole; no HR
Wasserman 1991	1991	E31, E14	Interim report; final = Lee 1995 (already included)

Supplementary Table S2. Risk of bias assessment using the Cochrane RoB 2 tool for all included studies.

**Study**	**Analysis**	**Treatment 1**	**Treatment 2**	**Setting**	D1: Randomisation	**D2: Deviations**	**D3: Missing data**	**D4: Measurement**	**D5: Reporting**	**Overall**	**Weight (1/SE<c2><b2>)**
Adelstein_2003	Main NMA	RT_ALONE	CIS_3W	Definitive	Low	Some concerns	Some concerns	Low	Some concerns	Some concerns	32.5
Bachaud_1996	Main NMA	RT_ALONE	CIS_WK	Adjuvant	Some concerns	Some concerns	Some concerns	Low	Some concerns	Some concerns	3.84
Bernier_2004	Main NMA	RT_ALONE	CIS_3W	Adjuvant	Low	Some concerns	Low	Low	Low	Some concerns	42.33
Bonner_2010	Main NMA	RT_ALONE	CETUX_RT	Definitive	Low	Some concerns	Low	Low	Low	Some concerns	54.95
Bourhis_2025_TrilynX	Main NMA	CIS_3W	XEVINAPANT_CRT	Definitive	Low	Low	Some concerns	Low	Low	Some concerns	44.74
Browman_1994	Main NMA	RT_ALONE	5FU_RT	Definitive	Some concerns	Low	Some concerns	Low	Some concerns	Some concerns	18.26
Calais_1999	Main NMA	RT_ALONE	CARBO_5FU_RT	Definitive	Low	Some concerns	Some concerns	Low	Some concerns	Some concerns	44.5
Caudell_2023	Main NMA	CIS_3W	CIS_3W_CETUX	Definitive	Low	Some concerns	Low	Low	Low	Some concerns	113.17
Cooper_2012	Main NMA	RT_ALONE	CIS_3W	Adjuvant	Low	Some concerns	Low	Low	Low	Some concerns	69.79
Eschwege_1997	Main NMA	RT_ALONE	ETANIDAZOLE_RT	Definitive	Low	Some concerns	Some concerns	Low	Some concerns	Some concerns	21.1
Fazekas_1987	Main NMA	RT_ALONE	MISO_RT	Definitive	Low	Some concerns	Some concerns	Low	Some concerns	Some concerns	11.4
Fietkau_2020	Main NMA	PAC_CIS_RT	CIS_5FU_RT	Definitive	Some concerns	Some concerns	Low	Low	Low	Some concerns	22.08
Forastiere_2013	Main NMA	CIS_3W	RT_ALONE	Definitive	Low	Some concerns	Low	Low	Some concerns	Some concerns	63.59
Gebre-Medhin_2025	Main NMA	CIS_WK	CETUX_RT	Definitive	Low	Some concerns	Low	Low	Low	Some concerns	20.64
Ghadjar_2012	Main NMA	RT_ALONE	CIS_DAILY	Definitive	Low	Some concerns	Low	Low	Low	Some concerns	32.1
Ghosh-Laskar_2016	Main NMA	RT_ALONE	CIS_WK	Definitive	High	Some concerns	Some concerns	Low	Some concerns	High	7.62
Gillison_2019	Main NMA	CETUX_RT	CIS_3W	Definitive	Low	Some concerns	Low	Low	Low	Some concerns	30.63
Giralt_2015	Main NMA	CIS_3W	PANI_RT	Definitive	Some concerns	Some concerns	Low	Low	Low	Some concerns	12.24
Henk_1977	Main NMA	RT_ALONE	HBO_RT	Definitive	Some concerns	Some concerns	Some concerns	Low	Some concerns	Some concerns	12.81
Henke_2003	Main NMA	RT_ALONE	EPO_RT	Mixed	Low	Low	Low	Low	Some concerns	Some concerns	48.43
Heukelom_2016	Main NMA	CIS_3W	IA_CIS_RT	Definitive	Low	Some concerns	Some concerns	Low	Some concerns	Some concerns	38.1
Hitt_2022	Main NMA	CIS_3W	CETUX_RT	Definitive	Low	Some concerns	Low	Low	Some concerns	Some concerns	56.45
Hoskin_2009	Main NMA	RT_ALONE	EPO_RT	Mixed	Low	Some concerns	Low	Low	Some concerns	Some concerns	36.73
Kiyota_2022	Main NMA	CIS_3W	CIS_WK	Adjuvant	Low	Some concerns	Low	Low	Low	Some concerns	22.92
Lee_1995	Main NMA	RT_ALONE	ETANIDAZOLE_RT	Definitive	Low	Some concerns	Low	Low	Low	Some concerns	84.95
Lee_2021	Main NMA	CIS_3W	AVELUMAB_CIS_3W	Definitive	Low	Low	Low	Low	Low	Low	32.58
Machiels_2024	Main NMA	CIS_3W	PEMBRO_CRT	Definitive	Low	Low	Low	Low	Low	Low	66.1
Maddalo_2020	Main NMA	CIS_WK	CETUX_RT	Definitive	Some concerns	Some concerns	Some concerns	Low	Some concerns	Some concerns	4.4

Mehanna_2019	Main NMA	CIS_3W	CETUX_RT	Definitive	Low	Some concerns	Low	Low	Low	Some concerns	3.21
Noronha_2025	Main NMA	CIS_WK	CIS_3W	Adjuvant	Some concerns	Some concerns	Low	Low	Low	Some concerns	44.5
Overgaard_1998	Main NMA	RT_ALONE	NIMORAZOLE_RT	Definitive	Low	Low	Low	Low	Some concerns	Some concerns	51.09
Patil_2019	Main NMA	CIS_WK	NIMO_CIS_WK	Definitive	Some concerns	Some concerns	Low	Low	Low	Some concerns	59.17
Patil_2023	Main NMA	RT_ALONE	DOC_WK_RT	Mixed	Some concerns	Some concerns	Low	Low	Low	Some concerns	51.91
Rischin_2021	Main NMA	CIS_WK	CETUX_RT	Definitive	Low	Some concerns	Low	Low	Low	Some concerns	4.45
Tao_2022	Main NMA	CETUX_RT	IMMUNO_CRT	Definitive	Low	Some concerns	Low	Low	Low	Some concerns	14.03
Tao_2023	Main NMA	CIS_3W	XEVINAPANT_CRT	Definitive	Low	Low	Low	Low	Low	Low	11.92
Thomson_2024	Main NMA	RT_ALONE	NIMORAZ	Definitive	Low	Low	Low	Low	Low	Low	31.18
Wendt_1998	Main NMA	RT_ALONE	CIS_5FU_RT	Definitive	Low	Some concerns	Some concerns	Low	Some concerns	Some concerns	15.6
Wozniak_2020	Main NMA	RT_ALONE	CIS_3W	Adjuvant	Some concerns	Some concerns	Low	Low	Low	Some concerns	10.81
Yi_2017	Main NMA	RT_ALONE	CIS_WK	Definitive	Some concerns	Some concerns	Low	Low	Low	Some concerns	24.6
Zhang_2023	Main NMA	CIS_3W	LOBAPLATIN_3W	Definitive	Some concerns	Some concerns	Low	Low	Low	Some concerns	6.16
Borel_2024	Sensitivity	CIS_3W	CIS_FRAC_3W	Mixed	Some concerns	Some concerns	Low	Low	Low	Some concerns	13.21
Bourhis_2011	Sensitivity	VERY_ACCEL_RT	CIS_5FU_RT	Definitive	Low	Some concerns	Some concerns	Low	Some concerns	Some concerns	22.89
Bourhis_2012	Sensitivity	VERY_ACCEL_RT	CARBO_5FU_RT	Definitive	Low	Some concerns	Low	Low	Low	Some concerns	100.8
Bourhis_2025_NIVOPOSTOP	Sensitivity	CIS_3W	NIVO_CIS_3W	Adjuvant	Low	Some concerns	High	Low	Some concerns	High	39.51
Dietz_2018	Sensitivity	INDUCTION_RT	INDUCTION_CETUX_RT	Definitive (larynx preservation)	Some concerns	Some concerns	Some concerns	Low	Low	Some concerns	13.13
Eriksen_2025	Sensitivity	NIMO_CIS_WK	ZALU_NIMO_CIS_WK	Definitive	Low	Some concerns	Low	Low	Low	Some concerns	47.24
Geoffrois_2018	Sensitivity	CARBO_5FU_RT	TPF_CETUX_RT	Definitive	Low	Some concerns	Low	Low	Low	Some concerns	54.87
Gregoire_2011	Sensitivity	CIS_3W	GEFIT_CIS_RT	Definitive	Low	Low	Low	Low	Low	Low	19.17
Gupta_2013	Sensitivity	CIS_5FU_RT	CIS_CAPE_RT	Definitive	High	Some concerns	Some concerns	Low	High	High	5.4
Gupta_2015	Sensitivity	ACCEL_RT	CIS_WK	Definitive	Some concerns	Some concerns	High	Low	Some concerns	High	13.52
Harrington_2013	Sensitivity	CIS_3W	LAPAT_CIS_RT	Definitive	Some concerns	Low	Low	Low	Low	Some concerns	7.51
Hassan_Metwally_2015	Sensitivity	ACCEL_RT	NIMO_ACCEL_RT	Definitive	High	Some concerns	Some concerns	Low	Low	High	10.26
Janoray_2020	Sensitivity	CIS_3W	CETUX_RT	Definitive	Low	Some concerns	Low	Low	Low	Some concerns	9.36
Janssens_2012	Sensitivity	ACCEL_RT	ARCON_ACCEL_RT	Definitive	Low	Some concerns	Low	Low	Some concerns	Some concerns	31.96
Martins_2013	Sensitivity	CIS_3W	ERLO_CIS_RT	Definitive	Some concerns	Some concerns	Low	Low	Low	Some concerns	9.57
Mell_2024	Sensitivity	CETUX_RT	DURVA_RT	Definitive	Low	Some concerns	Low	Low	Low	Some concerns	12.13
Merlano_2020	Sensitivity	CIS_3W	TPF_CETUX_RT	Definitive	High	High	Some concerns	Low	Low	High	25.64
Mesia_2015	Sensitivity	CIS_3W	PANI_CIS_RT	Definitive	Low	Some concerns	Low	Low	Low	Some concerns	10.1
Nguyen-Tan_2014	Sensitivity	CIS_3W	AFX_CIS_RT	Definitive	Low	Some concerns	Low	Low	Low	Some concerns	95.55
Rishi_2013	Sensitivity	CIS_3W	CBRT_RT	Definitive	High	Some	Low	Low	Low	High	9.93

						concerns					
Rodriguez_ 2015	Sensitivity	CIS_3W	CIS_5FU_ RT	Definitive	High	Some concerns	High	Low	High	High	1.95
Zandberg_2 025	Sensitivity	IMMUNO_ CRT	SEQ_PEM BRO_CIS_ WK	Definitive	Some concerns	Some concerns	Low	Low	Low	Some concerns	3.94

Supplementary Table S3. League table of all pairwise comparisons. Hazard ratios (95% CI) for overall survival; row treatment vs column treatment. Values below 1 favour the row treatment.

Cis + 5-FU	Paclitaxel + Cis	Nimotuzumab	Cisplatin wk	IA Cisplatin	Pembro + Cis wk	Hyperbaric O2	Pembro + CRT	Lobaplatin 3w	5-FU	Carbo + 5-FU	Docetaxel wk	Xevinapant + CRT	Cis + Cetuximab	Cisplatin daily	Cisplatin 3w	Cetuximab	Nimorazole	Etanidazole	Avelumab + Cis	RT alone	Panitumumab	Misonidazole	Erythropoietin
Cis + 5-FU	0.82 (0.46 - 1.47)	0.72 (0.30 - 1.69)	0.60 (0.30 - 1.22)	0.51 (0.22 - 1.21)	0.51 (0.19 - 1.35)	0.50 (0.20 - 1.29)	0.49 (0.21 - 1.11)	0.50 (0.16 - 1.52)	0.48 (0.19 - 1.16)	0.47 (0.21 - 1.06)	0.46 (0.21 - 1.05)	0.46 (0.21 - 1.01)	0.45 (0.20 - 1.02)	0.45 (0.19 - 1.05)	0.44 (0.22 - 0.86)	0.42 (0.21 - 0.86)	0.38 (0.16 - 0.88)	0.35 (0.17 - 0.74)	0.33 (0.14 - 0.79)	0.35 (0.18 - 0.66)	0.28 (0.10 - 0.73)	0.27 (0.10 - 0.72)	0.29 (0.14 - 0.60)
1.22 (0.68 - 2.19)	Paclitaxel + Cis	0.87 (0.31 - 2.47)	0.73 (0.29 - 1.84)	0.63 (0.22 - 1.77)	0.62 (0.20 - 1.93)	0.61 (0.20 - 1.86)	0.59 (0.21 - 1.64)	0.61 (0.17 - 2.15)	0.58 (0.20 - 1.69)	0.57 (0.21 - 1.56)	0.57 (0.21 - 1.54)	0.56 (0.21 - 1.49)	0.55 (0.20 - 1.50)	0.55 (0.20 - 1.53)	0.53 (0.22 - 1.31)	0.52 (0.21 - 1.29)	0.46 (0.16 - 1.28)	0.43 (0.17 - 1.11)	0.41 (0.14 - 1.16)	0.42 (0.18 - 1.01)	0.34 (0.11 - 1.04)	0.33 (0.11 - 1.03)	0.35 (0.14 - 0.90)
1.39 (0.59 - 3.28)	1.14 (0.41 - 3.23)	Nimotuzumab	0.84 (0.52 - 1.36)	0.72 (0.34 - 1.54)	0.71 (0.29 - 1.72)	0.70 (0.29 - 1.70)	0.68 (0.33 - 1.41)	0.69 (0.24 - 1.98)	0.66 (0.29 - 1.53)	0.65 (0.30 - 1.38)	0.65 (0.31 - 1.37)	0.64 (0.32 - 1.27)	0.63 (0.31 - 1.29)	0.63 (0.29 - 1.37)	0.61 (0.35 - 1.06)	0.59 (0.33 - 1.05)	0.53 (0.24 - 1.15)	0.49 (0.25 - 0.96)	0.47 (0.22 - 1.01)	0.48 (0.28 - 0.85)	0.38 (0.16 - 0.93)	0.38 (0.15 - 0.94)	0.40 (0.21 - 0.78)
1.66 (0.82 - 3.36)	1.36 (0.54 - 3.40)	1.19 (0.73 - 1.93)	Cisplatin wk	0.85 (0.48 - 1.54)	0.85 (0.40 - 1.77)	0.83 (0.40 - 1.75)	0.81 (0.47 - 1.40)	0.83 (0.33 - 2.09)	0.79 (0.40 - 1.56)	0.77 (0.43 - 1.38)	0.77 (0.44 - 1.36)	0.76 (0.46 - 1.24)	0.75 (0.44 - 1.27)	0.75 (0.41 - 1.38)	0.73 (0.55 - 0.95)	0.70 (0.51 - 0.96)	0.63 (0.34 - 1.15)	0.58 (0.37 - 0.93)	0.55 (0.30 - 1.01)	0.58 (0.43 - 0.76)	0.46 (0.22 - 0.96)	0.45 (0.21 - 0.98)	0.48 (0.30 - 0.75)
1.94 (0.83 - 4.56)	1.59 (0.57 - 4.49)	1.39 (0.65 - 2.98)	1.17 (0.65 - 2.10)	IA Cisplatin	0.99 (0.41 - 2.40)	0.98 (0.40 - 2.37)	0.94 (0.47 - 1.91)	0.97 (0.34 - 2.71)	0.92 (0.40 - 2.12)	0.90 (0.43 - 1.92)	0.90 (0.43 - 1.90)	0.89 (0.46 - 1.72)	0.88 (0.44 - 1.75)	0.88 (0.40 - 1.91)	0.85 (0.51 - 1.43)	0.82 (0.46 - 1.47)	0.73 (0.34 - 1.60)	0.68 (0.35 - 1.33)	0.65 (0.31 - 1.37)	0.67 (0.39 - 1.18)	0.53 (0.22 - 1.27)	0.53 (0.21 - 1.31)	0.56 (0.29 - 1.08)
1.96 (0.74 - 5.18)	1.61 (0.52 - 5.00)	1.41 (0.58 - 3.40)	1.18 (0.57 - 2.47)	1.01 (0.42 - 2.45)	Pembro + Cis wk	0.99 (0.36 - 2.68)	0.95 (0.40 - 2.26)	0.98 (0.31 - 3.06)	0.93 (0.36 - 2.42)	0.91 (0.38 - 2.21)	0.91 (0.38 - 2.19)	0.89 (0.39 - 2.04)	0.89 (0.38 - 2.07)	0.89 (0.36 - 2.19)	0.86 (0.42 - 1.76)	0.83 (0.43 - 1.62)	0.74 (0.30 - 1.83)	0.69 (0.31 - 1.56)	0.66 (0.27 - 1.61)	0.68 (0.33 - 1.41)	0.54 (0.20 - 1.47)	0.54 (0.19 - 1.48)	0.56 (0.25 - 1.27)
1.99 (0.78 - 5.10)	1.63 (0.54 - 4.94)	1.43 (0.59 - 3.46)	1.20 (0.57 - 2.51)	1.02 (0.42 - 2.48)	1.01 (0.37 - 2.75)	Hyperbaric O2	0.97 (0.41 - 2.28)	0.99 (0.32 - 3.10)	0.95 (0.38 - 2.38)	0.93 (0.39 - 2.17)	0.92 (0.40 - 2.15)	0.91 (0.40 - 2.07)	0.90 (0.38 - 2.09)	0.90 (0.38 - 2.14)	0.87 (0.43 - 1.78)	0.84 (0.40 - 1.77)	0.75 (0.31 - 1.80)	0.70 (0.32 - 1.52)	0.66 (0.27 - 1.62)	0.69 (0.35 - 1.37)	0.55 (0.20 - 1.48)	0.54 (0.20 - 1.46)	0.57 (0.26 - 1.24)
2.06 (0.90 - 4.71)	1.69 (0.61 - 4.66)	1.48 (0.71 - 3.07)	1.24 (0.72 - 2.15)	1.06 (0.52 - 2.15)	1.05 (0.44 - 2.48)	1.04 (0.44 - 2.45)	Pembro + CRT	1.02 (0.37 - 2.81)	0.98 (0.44 - 2.19)	0.96 (0.46 - 1.98)	0.96 (0.47 - 1.96)	0.94 (0.50 - 1.76)	0.93 (0.48 - 1.79)	0.93 (0.44 - 1.96)	0.90 (0.56 - 1.45)	0.87 (0.50 - 1.50)	0.78 (0.37 - 1.64)	0.72 (0.38 - 1.37)	0.69 (0.34 - 1.41)	0.71 (0.42 - 1.20)	0.57 (0.24 - 1.32)	0.56 (0.23 - 1.36)	0.59 (0.31 - 1.11)
2.01 (0.66 - 6.16)	1.65 (0.47 - 5.83)	1.44 (0.50 - 4.12)	1.21 (0.48 - 3.07)	1.04 (0.37 - 2.91)	1.02 (0.33 - 3.22)	1.01 (0.32 - 3.17)	0.98 (0.36 - 2.69)	Lobaplatin 3w	0.96 (0.32 - 2.88)	0.94 (0.33 - 2.66)	0.93 (0.33 - 2.64)	0.92 (0.34 - 2.45)	0.91 (0.33 - 2.46)	0.91 (0.31 - 2.62)	0.88 (0.36 - 2.14)	0.85 (0.34 - 2.16)	0.76 (0.26 - 2.20)	0.71 (0.26 - 1.89)	0.67 (0.24 - 1.90)	0.70 (0.28 - 1.74)	0.55 (0.18 - 1.71)	0.55 (0.17 - 1.75)	0.58 (0.22 - 1.54)
2.10 (0.86 - 5.13)	1.72 (0.59 - 5.02)	1.51 (0.65 - 3.47)	1.27 (0.64 - 2.50)	1.08 (0.47 - 2.49)	1.07 (0.41 - 2.78)	1.06 (0.42 - 2.66)	1.02 (0.46 - 2.29)	1.05 (0.35 - 3.15)	5-FU	0.98 (0.44 - 2.18)	0.98 (0.44 - 2.15)	0.96 (0.44 - 2.07)	0.95 (0.43 - 2.09)	0.95 (0.42 - 2.15)	0.92 (0.48 - 1.76)	0.89 (0.45 - 1.76)	0.79 (0.35 - 1.80)	0.74 (0.36 - 1.52)	0.70 (0.30 - 1.63)	0.73 (0.39 - 1.35)	0.58 (0.22 - 1.50)	0.58 (0.22 - 1.48)	0.60 (0.29 - 1.23)
2.15 (0.95 - 4.88)	1.76 (0.64 - 4.83)	1.54 (0.72 - 3.28)	1.29 (0.72 - 2.31)	1.11 (0.52 - 2.35)	1.09 (0.45 - 2.65)	1.08 (0.46 - 2.53)	1.04 (0.51 - 2.16)	1.07 (0.38 - 3.04)	1.02 (0.46 - 2.27)	Carbo + 5-FU	1.00 (0.49 - 2.02)	0.98 (0.49 - 1.94)	0.97 (0.48 - 1.97)	0.97 (0.46 - 2.03)	0.94 (0.54 - 1.62)	0.91 (0.51 - 1.63)	0.81 (0.39 - 1.70)	0.76 (0.40 - 1.41)	0.72 (0.33 - 1.54)	0.75 (0.45 - 1.24)	0.59 (0.24 - 1.43)	0.59 (0.25 - 1.41)	0.62 (0.33 - 1.15)
2.15 (0.95 - 4.85)	1.76 (0.65 - 4.81)	1.54 (0.73 - 3.26)	1.30 (0.73 - 2.29)	1.11 (0.53 - 2.34)	1.10 (0.46 - 2.64)	1.08 (0.46 - 2.52)	1.05 (0.51 - 2.14)	1.07 (0.38 - 3.03)	1.02 (0.46 - 2.26)	1.00 (0.49 - 2.03)	Docetaxel wk	0.98 (0.50 - 1.93)	0.97 (0.48 - 1.96)	0.97 (0.47 - 2.02)	0.94 (0.55 - 1.61)	0.91 (0.51 - 1.61)	0.81 (0.39 - 1.69)	0.76 (0.41 - 1.40)	0.72 (0.34 - 1.53)	0.75 (0.46 - 1.22)	0.59 (0.25 - 1.42)	0.59 (0.25 - 1.40)	0.62 (0.33 - 1.14)
2.19 (0.99 - 4.84)	1.80 (0.67 - 4.82)	1.57 (0.79 - 3.14)	1.32 (0.81 - 2.16)	1.13 (0.58 - 2.19)	1.12 (0.49 - 2.55)	1.10 (0.48 - 2.52)	1.07 (0.57 - 2.00)	1.09 (0.41 - 2.91)	1.04 (0.48 - 2.25)	1.02 (0.52 - 2.02)	1.02 (0.52 - 2.00)	Xevinapant + CRT	0.99 (0.54 - 1.82)	0.99 (0.49 - 2.01)	0.96 (0.64 - 1.45)	0.93 (0.57 - 1.51)	0.83 (0.41 - 1.68)	0.77 (0.43 - 1.39)	0.73 (0.37 - 1.44)	0.76 (0.48 - 1.20)	0.60 (0.27 - 1.35)	0.60 (0.26 - 1.40)	0.63 (0.35 - 1.13)
2.22 (0.98 - 5.00)	1.82 (0.67 - 4.96)	1.59 (0.78 - 3.25)	1.34 (0.79 - 2.26)	1.14 (0.57 - 2.27)	1.13 (0.48 - 2.64)	1.12 (0.48 - 2.60)	1.08 (0.56 - 2.08)	1.10 (0.41 - 2.99)	1.05 (0.48 - 2.33)	1.03 (0.51 - 2.10)	1.03 (0.51 - 2.07)	1.01 (0.55 - 1.86)	Cis + Cetuximab	1.00 (0.48 - 2.08)	0.97 (0.62 - 1.52)	0.94 (0.56 - 1.58)	0.84 (0.40 - 1.74)	0.78 (0.42 - 1.44)	0.74 (0.37 - 1.49)	0.77 (0.47 - 1.26)	0.61 (0.27 - 1.40)	0.61 (0.25 - 1.44)	0.64 (0.34 - 1.17)
2.22 (0.96 - 5.13)	1.82 (0.65 - 5.06)	1.59 (0.73 - 3.46)	1.34 (0.73 - 2.45)	1.14 (0.52 - 2.48)	1.13 (0.46 - 2.79)	1.11 (0.47 - 2.67)	1.08 (0.51 - 2.28)	1.10 (0.38 - 3.18)	1.05 (0.46 - 2.39)	1.03 (0.49 - 2.16)	1.03 (0.50 - 2.14)	1.01 (0.50 - 2.05)	1.00 (0.48 - 2.08)	Cisplatin daily	0.97 (0.55 - 1.72)	0.94 (0.51 - 1.73)	0.84 (0.39 - 1.79)	0.78 (0.41 - 1.50)	0.74 (0.34 - 1.63)	0.77 (0.45 - 1.32)	0.61 (0.25 - 1.50)	0.61 (0.25 - 1.48)	0.64 (0.33 - 1.22)
2.29 (1.16 - 4.50)	1.87 (0.76 - 4.59)	1.64 (0.94 - 2.86)	1.38 (1.05 - 1.81)	1.18 (0.70 - 1.98)	1.16 (0.57 - 2.39)	1.15 (0.56 - 2.35)	1.11 (0.69 - 1.79)	1.14 (0.47 - 2.77)	1.09 (0.57 - 2.08)	1.06 (0.62 - 1.84)	1.06 (0.62 - 1.81)	1.04 (0.69 - 1.57)	1.03 (0.66 - 1.62)	1.03 (0.58 - 1.83)	Cisplatin 3w	0.97 (0.74 - 1.26)	0.86 (0.48 - 1.54)	0.80 (0.53 - 1.22)	0.76 (0.45 - 1.31)	0.79 (0.65 - 0.97)	0.63 (0.31 - 1.26)	0.63 (0.30 - 1.31)	0.66 (0.43 - 0.99)

2.36 (1.17-4.79)	1.94 (0.77-4.85)	1.69 (0.95-3.02)	1.42 (1.04-1.96)	1.22 (0.68-2.18)	1.20 (0.62-2.35)	1.19 (0.57-2.50)	1.15 (0.67-1.98)	1.18 (0.46-2.98)	1.12 (0.57-2.22)	1.10 (0.61-1.97)	1.10 (0.62-1.95)	1.08 (0.66-1.76)	1.07 (0.63-1.80)	1.07 (0.58-1.96)	1.03 (0.79-1.35)	Cetuximab	0.89 (0.48-1.65)	0.83 (0.52-1.32)	0.79 (0.43-1.44)	0.82 (0.62-1.09)	0.65 (0.31-1.37)	0.65 (0.30-1.39)	0.68 (0.43-1.08)
2.65 (1.14-6.15)	2.17 (0.78-6.07)	1.90 (0.87-4.15)	1.60 (0.87-2.94)	1.36 (0.63-2.97)	1.35 (0.55-3.34)	1.33 (0.56-3.19)	1.29 (0.61-2.73)	1.32 (0.46-3.81)	1.26 (0.55-2.86)	1.23 (0.59-2.59)	1.23 (0.59-2.56)	1.21 (0.59-2.46)	1.20 (0.57-2.49)	1.20 (0.56-2.57)	1.16 (0.65-2.07)	1.12 (0.61-2.07)	Nimorazole	0.93 (0.49-1.79)	0.89 (0.40-1.95)	0.92 (0.54-1.58)	0.73 (0.30-1.80)	0.72 (0.30-1.77)	0.76 (0.40-1.46)
2.84 (1.35-5.96)	2.33 (0.90-6.00)	2.04 (1.04-3.98)	1.71 (1.08-2.72)	1.46 (0.75-2.85)	1.45 (0.64-3.26)	1.43 (0.66-3.11)	1.38 (0.73-2.61)	1.41 (0.53-3.78)	1.35 (0.66-2.77)	1.32 (0.71-2.47)	1.32 (0.71-2.44)	1.30 (0.72-2.33)	1.28 (0.69-2.37)	1.28 (0.67-2.46)	1.24 (0.82-1.89)	1.20 (0.76-1.91)	1.07 (0.56-2.06)	Etanidazole	0.95 (0.48-1.88)	0.99 (0.68-1.42)	0.78 (0.35-1.76)	0.78 (0.35-1.73)	0.81 (0.49-1.36)
2.99 (1.26-7.10)	2.45 (0.86-6.97)	2.15 (0.99-4.65)	1.80 (0.99-3.29)	1.54 (0.73-3.25)	1.53 (0.62-3.74)	1.51 (0.62-3.68)	1.45 (0.71-2.98)	1.49 (0.53-4.21)	1.42 (0.61-3.31)	1.39 (0.65-3.00)	1.39 (0.65-2.97)	1.37 (0.69-2.68)	1.35 (0.67-2.72)	1.35 (0.61-2.97)	1.31 (0.77-2.24)	1.27 (0.70-2.31)	1.13 (0.51-2.49)	1.05 (0.53-2.08)	Avelumab + Cis	1.04 (0.59-1.85)	0.82 (0.34-1.98)	0.82 (0.33-2.04)	0.86 (0.44-1.69)
2.88 (1.51-5.49)	2.36 (0.99-5.65)	2.07 (1.18-3.62)	1.74 (1.31-2.30)	1.48 (0.85-2.59)	1.47 (0.71-3.03)	1.45 (0.73-2.88)	1.40 (0.83-2.35)	1.43 (0.57-3.57)	1.37 (0.74-2.54)	1.34 (0.81-2.23)	1.34 (0.82-2.19)	1.31 (0.83-2.08)	1.30 (0.79-2.13)	1.30 (0.76-2.23)	1.26 (1.03-1.55)	1.22 (0.91-1.63)	1.09 (0.63-1.87)	1.01 (0.70-1.46)	0.96 (0.54-1.71)	RT alone	0.79 (0.38-1.64)	0.79 (0.39-1.61)	0.83 (0.58-1.19)
3.63 (1.38-9.59)	2.98 (0.96-9.26)	2.61 (1.07-6.35)	2.19 (1.04-4.62)	1.87 (0.78-4.46)	1.85 (0.68-5.03)	1.83 (0.67-4.96)	1.77 (0.76-4.11)	1.81 (0.58-5.60)	1.73 (0.67-4.48)	1.69 (0.70-4.10)	1.69 (0.70-4.06)	1.66 (0.74-3.72)	1.64 (0.72-3.76)	1.64 (0.66-4.05)	1.59 (0.79-3.19)	1.54 (0.73-3.24)	1.37 (0.55-3.39)	1.28 (0.57-2.88)	1.21 (0.50-2.92)	1.26 (0.61-2.60)	Panitumumab	0.99 (0.36-2.75)	1.04 (0.46-2.34)
3.66 (1.40-9.56)	3.00 (0.97-9.24)	2.62 (1.06-6.49)	2.20 (1.02-4.74)	1.88 (0.76-4.66)	1.86 (0.67-5.15)	1.84 (0.68-4.94)	1.78 (0.74-4.29)	1.82 (0.57-5.79)	1.74 (0.68-4.46)	1.70 (0.71-4.08)	1.70 (0.71-4.04)	1.67 (0.71-3.89)	1.65 (0.69-3.93)	1.65 (0.68-4.03)	1.60 (0.76-3.36)	1.55 (0.72-3.33)	1.38 (0.56-3.38)	1.29 (0.58-2.87)	1.22 (0.49-3.05)	1.27 (0.62-2.59)	1.01 (0.36-2.78)	Misonidazole	1.05 (0.47-2.33)
3.49 (1.66-7.31)	2.86 (1.11-7.35)	2.50 (1.28-4.88)	2.10 (1.33-3.33)	1.80 (0.92-3.50)	1.78 (0.79-4.00)	1.75 (0.81-3.81)	1.70 (0.90-3.19)	1.73 (0.65-4.64)	1.66 (0.81-3.39)	1.62 (0.87-3.03)	1.62 (0.88-2.99)	1.59 (0.89-2.85)	1.57 (0.85-2.91)	1.57 (0.82-3.01)	1.53 (1.01-2.31)	1.48 (0.93-2.34)	1.32 (0.69-2.52)	1.23 (0.73-2.05)	1.17 (0.59-2.30)	1.21 (0.84-1.74)	0.96 (0.43-2.16)	0.95 (0.43-2.12)	Erythropoietin

Supplementary Table S4. Comparison of frequentist P-scores and Bayesian SUCRA rankings for all treatment nodes.

Treatment	P-score (%)	SUCRA (%)	Freq Mean Rank	|P-score - SUCRA|	Agreement
Cis + 5-FU	94.8	94.4	0.9	0.3999999999999915	Strong
Paclitaxel + Cis	84.4	84.4	0.8	0	Strong
Nimotuzumab	84.2	83.9	0.8	0.2999999999999972	Strong
Cisplatin wk	77.5	77.1	0.8	0.4000000000000057	Strong
IA Cisplatin	62.1	62.4	0.6	0.2999999999999972	Strong
Pembro + Cis wk	59.7	59.8	0.6	0.09999999999999432	Strong
Hyperbaric O2	58.9	59.3	0.6	0.3999999999999986	Strong
Pembro + CRT	57.5	57.7	0.6	0.2000000000000028	Strong
Lobaplatin 3w	56.7	56.9	0.6	0.1999999999999957	Strong
5-FU	54.8	55.3	0.5	0.5	Strong
Carbo + 5-FU	53.6	54	0.5	0.3999999999999986	Strong
Docetaxel wk	53.5	53.8	0.5	0.2999999999999972	Strong
Xevinapant + CRT	51.9	49.9	0.5	2	Strong
Cis + Cetuximab	50.8	50.6	0.5	0.1999999999999957	Strong
Cisplatin daily	50.7	51	0.5	0.2999999999999972	Strong
Cisplatin 3w	48.3	48.1	0.5	0.1999999999999957	Strong
Cetuximab	44.4	44.5	0.4	0.1000000000000014	Strong
Nimorazole	35.2	35.3	0.4	0.09999999999999432	Strong
Etanidazole	27.5	27.6	0.3	0.1000000000000014	Strong
Avelumab + Cis	25.4	25.3	0.3	0.09999999999999787	Strong
RT alone	23.6	23.8	0.2	0.1999999999999993	Strong
Misonidazole	16	15.9	0.2	0.09999999999999964	Strong
Panitumumab	16	16.1	0.2	0.1000000000000014	Strong
Erythropoietin	12.6	12.9	0.1	0.3000000000000007	Strong

Supplementary Table S5. Treatment setting subgroup analysis. Studies classified by treatment setting (definitive, adjuvant, mixed) with P-score comparison.

Sheet: Studies by Setting

Study	Setting	T1	T2	HR (95% CI)	Significant	HPV Status	Sensitivity Only
Bachaud_1996	Adjuvant	RT alone	Cisplatin wk	0.26 (0.10-0.71)	True	NR	No
Bernier_2004	Adjuvant	RT alone	Cisplatin 3w	0.70 (0.52-0.95)	True	NR	No
Bourhis_2025_NIVOPOSTOP	Adjuvant	Cisplatin 3w	Nivo + Cis 3w	0.84 (0.62-1.15)	False	Mixed (5% p16+)	Yes
Cooper_2012	Adjuvant	RT alone	Cisplatin 3w	0.89 (0.70-1.12)	False	NR	No
Kiyota_2022	Adjuvant	Cisplatin 3w	Cisplatin wk	0.75 (0.50-1.13)	False	NR	No
Noronha_2025	Adjuvant	Cisplatin wk	Cisplatin 3w	1.21 (0.90-1.62)	False	NR	No
Wozniak_2020	Adjuvant	RT alone	Cisplatin 3w	1.03 (0.57-1.87)	False	NR	No
Adelstein_2003	Definitive	RT alone	Cisplatin 3w	0.68 (0.48-0.96)	True	NR	No
Bonner_2010	Definitive	RT alone	Cetuximab	0.73 (0.56-0.95)	True	NR	No
Bourhis_2011	Definitive	Very accel RT	Cis + 5-FU	0.95 (0.63-1.43)	False	NR	Yes
Bourhis_2012	Definitive	Very accel RT	Carbo + 5-FU	0.81 (0.67-0.99)	True	NR	Yes
Bourhis_2025_TrilynX	Definitive	Cisplatin 3w	Xevinapant + CRT	1.39 (1.04-1.86)	True	HPV_Negative	No
Browman_1994	Definitive	RT alone	5-FU	0.73 (0.46-1.15)	False	NR	No
Calais_1999	Definitive	RT alone	Carbo + 5-FU	0.75 (0.56-1.00)	False	NR	No
Caudell_2023	Definitive	Cisplatin 3w	Cis + Cetuximab	0.97 (0.80-1.16)	False	Mixed (235 p16+ OPC)	No
Eriksen_2025	Definitive	Nimotuzumab	Zalu + Nimo + Cis	1.21 (0.91-1.61)	False	Mixed (55% p16+)	Yes
Eschwege_1997	Definitive	RT alone	Etanidazole	1.05 (0.68-1.61)	False	NR	No
Fazekas_1987	Definitive	RT alone	Misonidazole	1.27 (0.71-2.27)	False	NR	No
Fietkau_2020	Definitive	Paclitaxel + Cis	Cis + 5-FU	0.82 (0.54-1.24)	False	NR	No
Forastiere_2013	Definitive	Cisplatin 3w	RT alone	1.08 (0.85-1.39)	False	NR	No
Gebre-Medhin_2025	Definitive	Cisplatin wk	Cetuximab	1.66 (1.08-2.56)	True	p16+ (89% of OPC)	No
Geoffrois_2018	Definitive	Carbo + 5-FU	TPF + Cetux RT	1.12 (0.86-1.46)	False	Mixed (74% p16-)	Yes
Ghadjar_2012	Definitive	RT alone	Cisplatin daily	0.77 (0.56-1.11)	False	NR	No
Ghosh-Laskar_2016	Definitive	RT alone	Cisplatin wk	0.48 (0.24-0.97)	True	NR	No
Gillison_2019	Definitive	Cetuximab	Cisplatin 3w	1.45 (1.03-2.05)	True	HPV_Positive	No
Giralt_2015	Definitive	Cisplatin 3w	Panitumumab	1.59 (0.91-2.79)	False	p16- (76% of tested)	No
Gregoire_2011	Definitive	Cisplatin 3w	Gefitinib + Cis	1.06 (0.68-1.66)	False	NR	Yes
Gupta_2013	Definitive	Cis + 5-FU	Cis + Cape	1.67 (0.72-3.89)	False	NR	Yes
Gupta_2015	Definitive	Accelerated RT	Cisplatin wk	1.02 (0.60-1.74)	False	NR	Yes
Harrington_2013	Definitive	Cisplatin 3w	Lapatinib + Cis	0.90 (0.44-1.84)	False	NR	Yes
Hassan_Metwally_2015	Definitive	Accelerated RT	Nimo + Accel RT	0.65 (0.35-1.19)	False	NR	Yes
Henk_1977	Definitive	RT alone	Hyperbaric O2	0.69 (0.40-1.20)	False	NR	No
Heukelom_2016	Definitive	Cisplatin 3w	IA Cisplatin	0.85 (0.62-1.17)	False	NR	No
Hitt_2022	Definitive	Cisplatin 3w	Cetuximab	1.11 (0.85-1.44)	False	Mixed (majority p16-)	No
Janoray_2020	Definitive	Cisplatin 3w	Cetuximab	1.04 (0.55-1.98)	False	NR	Yes
Janssens_2012	Definitive	Accelerated RT	ARCON + Accel RT	1.03 (0.73-1.46)	False	NR	Yes
Lee_1995	Definitive	RT alone	Etanidazole	0.95 (0.77-1.17)	False	NR	No
Lee_2021	Definitive	Cisplatin 3w	Avelumab + Cis	1.31 (0.93-1.85)	False	Mixed	No
Machiels_2024	Definitive	Cisplatin 3w	Pembro + CRT	0.90 (0.71-1.15)	False	HPV_Negative	No

Maddalo_2020	Definitive	Cisplatin wk	Cetuximab	1.41 (0.55-3.59)	False	Mixed	No
Martins_2013	Definitive	Cisplatin 3w	Erlotinib + Cis	1.05 (0.56-1.98)	False	Mixed	Yes
Mehanna_2019	Definitive	Cisplatin 3w	Cetuximab	5.00 (1.70-14.70)	True	HPV_Positive	No
Mell_2024	Definitive	Cetuximab	Durvalumab	1.30 (0.74-2.28)	False	Mixed	Yes
Merlano_2020	Definitive	Cisplatin 3w	TPF + Cetux RT	1.05 (0.71-1.54)	False	NR	Yes
Mesia_2015	Definitive	Cisplatin 3w	Pani + Cis	1.63 (0.88-3.02)	False	Mixed	Yes
Nguyen-Tan_2014	Definitive	Cisplatin 3w	AFX + Cis + RT	0.96 (0.79-1.18)	False	Mixed	Yes
Patil_2019	Definitive	Cisplatin wk	Nimotuzumab	0.84 (0.65-1.08)	False	HPV_Negative	No
Rishi_2013	Definitive	Cisplatin 3w	Con. boost RT	0.73 (0.34-1.18)	False	NR	Yes
Rodriguez_2015	Definitive	Cisplatin 3w	Cis + 5-FU	5.30 (1.30-21.60)	True	HPV_Positive (71%)	Yes
Tao_2022	Definitive	Cetuximab	Pembro + Cis wk	0.83 (0.49-1.40)	False	HPV_Negative	No
Tao_2023	Definitive	Cisplatin 3w	Xevinapant + CRT	0.47 (0.27-0.84)	True	Mixed (mostly HPV-negative)	No
Thomson_2024	Definitive	RT alone	Nimorazole	0.92 (0.65-1.31)	False	Mixed	No
Wendt_1998	Definitive	RT alone	Cis + 5-FU	0.35 (0.21-0.57)	True	NR	No
Yi_2017	Definitive	RT alone	Cisplatin wk	0.74 (0.50-1.10)	False	NR	No
Zandberg_2025	Definitive	Pembro + Cis wk	Seq Pembro + Cis	0.51 (0.19-1.37)	False	Mixed (53% HPV+)	Yes
Zhang_2023	Definitive	Cisplatin 3w	Lobaplatin 3w	0.88 (0.40-1.94)	False	NR	No
Dietz_2018	Definitive (larynx preservation)	Post-ind RT	Post-ind Cetux	1.04 (0.60-1.78)	False	NR	Yes
Borel_2024	Mixed	Cisplatin 3w	Cis frac 3w	0.86 (0.50-1.47)	False	NR	Yes
Henke_2003	Mixed	RT alone	Erythropoietin	1.39 (1.05-1.84)	True	NR	No
Hoskin_2009	Mixed	RT alone	Erythropoietin	1.04 (0.75-1.43)	False	NR	No
Patil_2023	Mixed	RT alone	Docetaxel wk	0.75 (0.57-0.98)	True	NR	No

Sheet: P-score Comparison

Treatment	P-score All (%)	Rank All	P-score Definitive (%)	Rank Definitive	|Rank Diff|	|P-score Diff|
Cis + 5-FU	94.8	0.9	92.6	0.9	0	2.200000000000003
Paclitaxel + Cis	84.4	0.8	81.5	0.8	0	2.900000000000006
Nimotuzumab	84.2	0.8	79.7	0.8	0	4.5
Cisplatin wk	77.5	0.8	73.1	0.7	0.1000000000000001	4.400000000000006
IA Cisplatin	62.1	0.6	60.9	0.6	0	1.200000000000003
Pembro + Cis wk	59.7	0.6	57	0.6	0	2.700000000000003
Hyperbaric O2	58.9	0.6	57.8	0.6	0	1.100000000000001
Pembro + CRT	57.5	0.6	56.8	0.6	0	0.7000000000000028
Lobaplatin 3w	56.7	0.6	56.5	0.6	0	0.2000000000000028
5-FU	54.8	0.5	54.2	0.5	0	0.5999999999999943
Carbo + 5-FU	53.6	0.5	53	0.5	0	0.6000000000000014
Docetaxel wk	53.5	0.5	52.9	0.5	0	0.6000000000000014

Xevinapant + CRT	51.9	0.5	55.8	0.6	0.09999999999999998	3.899999999999999
Cis + Cetuximab	50.8	0.5	51	0.5	0	0.2000000000000028
Cisplatin daily	50.7	0.5	50.5	0.5	0	0.2000000000000028
Cisplatin 3w	48.3	0.5	49	0.5	0	0.7000000000000028
Cetuximab	44.4	0.4	42.6	0.4	0	1.799999999999997
Nimorazole	35.2	0.4	36.9	0.4	0	1.699999999999996
Etanidazole	27.5	0.3	29.7	0.3	0	2.199999999999999
Avelumab + Cis	25.4	0.3	28.7	0.3	0	3.300000000000001
RT alone	23.6	0.2	26.2	0.3	0.09999999999999998	2.599999999999998
Misonidazole	16	0.2	18.6	0.2	0	2.600000000000001
Panitumumab	16	0.2	19.2	0.2	0	3.199999999999999
Erythropoietin	12.6	0.1	15.9	0.2	0.1	3.300000000000001

Supplementary Table S6. Sensitivity analysis studies. Characteristics and rationale for the 23 studies included only in the sensitivity analysis.

Study	Arm 1 (T1)	Arm 2 (T2)	Outcome	HR (95% CI)	Setting	HPV Status	Classification Reason	Flag
Bourhis_2011	Very accel RT	Cis + 5-FU	OS	0.95 (0.63-1.43)	Definitive	NR	VERY_ACCEL_RT sensitivity node	
Gregoire_2011	Cisplatin 3w	Gefitinib + Cis	OS	1.06 (0.68-1.66)	Definitive	NR	GEFIT_CIS_RT dead-end node	
Bourhis_2012	Very accel RT	Carbo + 5-FU	OS	0.81 (0.67-0.99)	Definitive	NR	VERY_ACCEL_RT sensitivity node	
Janssens_2012	Accelerated RT	ARCON + Accel RT	OS	1.03 (0.73-1.46)	Definitive	NR	ARCON_ACCEL_RT, larynx only, 4Gy dose reduction	
Harrington_2013	Cisplatin 3w	Lapatinib + Cis	OS	0.90 (0.44-1.84)	Definitive	NR	LAPAT_CIS_RT dead-end node, Phase IIb, n=67	
Martins_2013	Cisplatin 3w	Erlotinib + Cis	OS	1.05 (0.56-1.98)	Definitive	Mixed	ERLO_CIS_RT dead-end node, Phase II	
Rishi_2013	Cisplatin 3w	Con. boost RT	OS	0.73 (0.34-1.18)	Definitive	NR	CBRT_RT dead-end node, single institution	
Gupta_2013	Cis + 5-FU	Cis + Cape	OS	1.67 (0.72-3.89)	Definitive	NR	CIS_CAPE_RT dead-end node	
Nguyen-Tan_2014	Cisplatin 3w	AFX + Cis + RT	OS	0.96 (0.79-1.18)	Definitive	Mixed	AFX_CIS_RT (confounds fractionation + CIS dose)	
Gupta_2015	Accelerated RT	Cisplatin wk	OS	1.02 (0.60-1.74)	Definitive	NR	ACCEL_RT sensitivity node (E22 revised)	
Hassan_Metwally_2015	Accelerated RT	Nimo + Accel RT	OS	0.65 (0.35-1.19)	Definitive	NR	NIMO_ACCEL_RT, 14% accrual	
Mesia_2015	Cisplatin 3w	Pani + Cis	OS	1.63 (0.88-3.02)	Definitive	Mixed	PANI_CIS_RT (confounded CIS doses: 100 vs 75mg/m2)	
Rodriguez_2015	Cisplatin 3w	Cis + 5-FU	OS	5.30 (1.30-21.60)	Definitive	HPV_Positive (71%)	CIS_3W vs CIS_5FU_RT link, extreme outlier	EXTREME OUTLIER (9 deaths total, SE=0.72)
Dietz_2018	Post-ind RT	Post-ind Cetux	OS	1.04 (0.60-1.78)	Definitive (larynx preservation)	NR	INDUCTION_RT/ INDUCTION_CETUX_RT disconnected from main network	
Geoffrois_2018	Carbo + 5-FU	TPF + Cetux RT	OS	1.12 (0.86-1.46)	Definitive	Mixed (74% p16-)	TPF_CETUX_RT (2nd	ASYMMETRIC

							study), asymmetric induction	INDUCTION
Janoray_2020	Cisplatin 3w	Cetuximab	OS	1.04 (0.55-1.98)	Definitive	NR	Post-TPF CIS vs CETUX (E22 revised, like Hitt_2022)	
Merlano_2020	Cisplatin 3w	TPF + Cetux RT	OS	1.05 (0.71-1.54)	Definitive	NR	TPF_CETUX_RT, asymmetric induction, underpowered	ASYMMETRIC INDUCTION
Mell_2024	Cetuximab	Durvalumab	OS	1.30 (0.74-2.28)	Definitive	Mixed	DURVA_RT sensitivity node (E22 revised)	
Borel_2024	Cisplatin 3w	Cis frac 3w	OS	0.86 (0.50-1.47)	Mixed	NR	Same-drug-different-timing (fractionated vs bolus CIS)	
Eriksen_2025	Nimotuzumab	Zalu + Nimo + Cis	OS	1.21 (0.91-1.61)	Definitive	Mixed (55% p16+)	ZALU_NIMO_CIS_WK dead-end, abandoned drug	
Zandberg_2025	Pembro + Cis wk	Seq Pembro + Cis	OS	0.51 (0.19-1.37)	Definitive	Mixed (53% HPV+)	SEQ_PEMBRO_CIS_WK, Phase II pick-the-winner	
Bourhis_2025_NIVOPOSTOP	Cisplatin 3w	Nivo + Cis 3w	OS	0.84 (0.62-1.15)	Adjuvant	Mixed (5% p16+)	NIVO_CIS_3W, OS HR estimated (DFS primary)	OS NOT MATURE (Peto estimate)

Supplementary Table S7. Excluded studies with reasons for exclusion.

Study	Exclusion.Code	Reason
Ameri_2022	E14	Toxicity-only, no efficacy data, mixed population
Bakowski_1978	E14	No OS data reported
Bartelink_2002	E22,E28	Confounded comparison, Phase I/II
Brizel_1998	E22	Hyperfractionated RT arms, not standard fractionation
Budach_2015	E22	No network connectivity
Chassagne_1992	E14	No OS data reported
Chauhan_2008	E15	No HR estimable
de_Leeuw_2024	E22	RT technique comparison, not radiosensitiser
Fu_2000	E22	No concurrent drug difference (RT fractionation comparison)
Halim_2012	E22	No network connectivity
Harari_2014	E22	No network connectivity (postoperative cetuximab)
Huilgol_1996	E14,E28	No OS data, Phase IIa single-arm
Lefebvre_2013	E31	Duplicate report of Janoray_2020 (TREMPLIN)
Ma_2025	E22,E33	De-escalation RT, no network connectivity
Mendenhall_2005	E15,E22	No HR estimable, no network connectivity
Mohanty_2025	E22,E28,E15	No OS HR, Phase IIb, no network connectivity
Overgaard_1989	E15	No HR estimable (KM only, no events for Peto)
Overgaard_2003	E22	RT fractionation comparison, no concurrent drug
Palma_2025	E26	RT vs surgery comparison
Panis_1984	E15	No HR estimable
Quon_2011	E15	No HR estimable (proportions only)
Rosenthal_2016	E31	p16 subanalysis of Bonner_2010 (parent included)
Sealy_1986	E15	No HR estimable
Tao_2020	E27	Safety/protocol report only (REACH, avelumab)
Ullal_2006	E14,E28	No OS data, Phase IIa, sanazole
Van_den_Bogaert_1995	E15	No HR estimable
Wasserman_1991	E31	Secondary analysis of etanidazole (Eschwege_1997 included)

Supplementary Table S8. Node-splitting results for assessment of local inconsistency between direct and indirect evidence.

Comparison	Direct HR (95% CI)	Indirect HR (95% CI)	NMA HR (95% CI)	p (inconsistency)	Significant
CIS_3W:CIS_WK	1.26 (0.86-1.85)	1.50 (1.02-2.21)	1.38 (1.05-1.81)	0.524	
CIS_3W:RT_ALONE	0.82 (0.65-1.04)	0.71 (0.47-1.07)	0.79 (0.65-0.97)	0.535	
CETUX_RT:RT_ALONE	0.73 (0.45-1.19)	0.87 (0.61-1.24)	0.82 (0.62-1.09)	0.564	
CETUX_RT:CIS_WK	1.59 (0.95-2.67)	1.33 (0.89-1.99)	1.42 (1.04-1.96)	0.589	
CIS_WK:RT_ALONE	0.56 (0.36-0.86)	0.59 (0.41-0.86)	0.58 (0.43-0.76)	0.842	
CETUX_RT:CIS_3W	1.04 (0.74-1.47)	1.02 (0.68-1.54)	1.03 (0.79-1.35)	0.946	
5FU_RT:RT_ALONE	0.73 (0.39-1.35)	NA (NA-NA)	0.73 (0.39-1.35)	—	
AVELUMAB_CIS_3W:CIS_3W	1.31 (0.77-2.24)	NA (NA-NA)	1.31 (0.77-2.24)	—	
CARBO_5FU_RT:RT_ALONE	0.75 (0.45-1.24)	NA (NA-NA)	0.75 (0.45-1.24)	—	
CETUX_RT:IMMUNO_CRT	1.20 (0.62-2.35)	NA (NA-NA)	1.20 (0.62-2.35)	—	
CIS_3W:CIS_3W_CETUX	1.03 (0.66-1.62)	NA (NA-NA)	1.03 (0.66-1.62)	—	
CIS_3W:IA_CIS_RT	1.18 (0.70-1.98)	NA (NA-NA)	1.18 (0.70-1.98)	—	
CIS_3W:LOBAPLATIN_3W	1.14 (0.47-2.77)	NA (NA-NA)	1.14 (0.47-2.77)	—	
CIS_3W:PANI_RT	0.63 (0.31-1.26)	NA (NA-NA)	0.63 (0.31-1.26)	—	
CIS_3W:PEMBRO_CRT	1.11 (0.69-1.79)	NA (NA-NA)	1.11 (0.69-1.79)	—	
CIS_3W:XEVINAPANT_CRT	1.04 (0.69-1.57)	NA (NA-NA)	1.04 (0.69-1.57)	—	
CIS_5FU_RT:PAC_CIS_RT	0.82 (0.46-1.47)	NA (NA-NA)	0.82 (0.46-1.47)	—	
CIS_5FU_RT:RT_ALONE	0.35 (0.18-0.66)	NA (NA-NA)	0.35 (0.18-0.66)	—	
CIS_DAILY:RT_ALONE	0.77 (0.45-1.32)	NA (NA-NA)	0.77 (0.45-1.32)	—	
CIS_WK:NIMO_CIS_WK	1.19 (0.73-1.93)	NA (NA-NA)	1.19 (0.73-1.93)	—	
DOC_WK_RT:RT_ALONE	0.75 (0.46-1.22)	NA (NA-NA)	0.75 (0.46-1.22)	—	
EPO_RT:RT_ALONE	1.21 (0.84-1.74)	NA (NA-NA)	1.21 (0.84-1.74)	—	
ETANIDAZOLE_RT:RT_ALONE	0.99 (0.68-1.42)	NA (NA-NA)	0.99 (0.68-1.42)	—	
HBO_RT:RT_ALONE	0.69 (0.35-1.37)	NA (NA-NA)	0.69 (0.35-1.37)	—	
MISO_RT:RT_ALONE	1.27 (0.62-2.59)	NA (NA-NA)	1.27 (0.62-2.59)	—	
NIMORAZ:RT_ALONE	0.92 (0.54-1.58)	NA (NA-NA)	0.92 (0.54-1.58)	—	

Supplementary Table S9. Heterogeneity assessment. Global and per-comparison heterogeneity statistics.

Sheet: Global Heterogeneity

Model	tau2	I-squared (%)	Q	df	p_value	Design-by-treatment Q	Design-by-treatment df	Design-by-treatment p
Primary NMA (main)	0.0443	56.1	36.45	16	0.0025	1.92	3	0.59
Full Sensitivity NMA	0.0565	60.5	50.67	20	0.000177	16.12	6	0.0131

Sheet: Per-Comparison

Comparison	N Studies	I-squared (%)	tau2	Q	p (heterogeneity)	Studies
CIS_3W vs RT_ALONE	5			0	NA	Wozniak_2020; Forastiere_2013; Cooper_2012; Bernier_2004; Adelstein_2003
CETUX_RT vs CIS_3W	3			0	NA	Gillison_2019; Mehanna_2019; Hitt_2022
CIS_WK vs RT_ALONE	3			0	NA	Yi_2017; Ghosh-Laskar_2016; Bachaud_1996
CETUX_RT vs CIS_WK	2			0	NA	Gebre-Medhin_2025; Maddalo_2020
CIS_3W vs CIS_WK	2			0	NA	Noronha_2025; Kiyota_2022
CIS_3W vs XEVINAPANT_CRT	2			0	NA	Tao_2023; Bourhis_2025_TrilynX
EPO_RT vs RT_ALONE	2			0	NA	Henke_2003; Hoskin_2009
ETANIDAZOLE_RT vs RT_ALONE	2			0	NA	Eschwege_1997; Lee_1995

Supplementary Table S10. Toxicity profile by treatment node. Rates of grade 3+ adverse events reported across included studies.

Drug Class	Treatment	NMA Node	Toxicity Category	Toxicity Term	N Studies	Total Patients	Median G3-4 (%)	Weighted G3-4 (%)
EGFR-targeted	Cetuximab	CETUX_RT	Acute	Grade 3+ adverse events	1	65	92	92
EGFR-targeted	Cetuximab	CETUX_RT	Acute	Dermatitis/ Skin reaction	2	155	56.5	56.1
EGFR-targeted	Cetuximab	CETUX_RT	Acute	Mucositis/ Stomatitis	4	639	51.6	48
EGFR-targeted	Cetuximab	CETUX_RT	Acute	Dysphagia	2	180	33.5	33.5
EGFR-targeted	Cetuximab	CETUX_RT	Acute	Weight loss	1	90	26	26
EGFR-targeted	Cetuximab	CETUX_RT	Acute	Rash/ Acneiform	2	155	17.5	17.9
EGFR-targeted	Cetuximab	CETUX_RT	Acute	Fatigue	1	90	3	3
EGFR-targeted	Cetuximab	CETUX_RT	Acute	Neutropenia/ Leukopenia	2	484	0.2	0.4
EGFR-targeted	Cetuximab	CETUX_RT	Acute	Nausea/ Vomiting	1	90	0	0
EGFR-targeted	Cetuximab	CETUX_RT	Late	Hearing impairment	1	375	2.1	2.1
EGFR-targeted	Cetuximab	CETUX_RT	Overall	Treatment-related death	1	165		
EGFR-targeted	Nimotuzumab	NIMO_CIS_WK	Acute	Mucositis/ Stomatitis	1	267	66.7	66.7
EGFR-targeted	Nimotuzumab	NIMO_CIS_WK	Acute	Treatment-related death	1	267		
Hypoxic modifier	Hyperbaric O2	HBO_RT	Late	Perichondritis	1	51	2	2
Immunotherapy	Pembro + Cis wk	IMMUNO_CRT	Acute	Grade 3+ adverse events	1	66	74	74
Immunotherapy	Pembro + Cis wk	IMMUNO_CRT	Acute	Mucositis/ Stomatitis	1	66	32	32
Immunotherapy	Pembro + Cis wk	IMMUNO_CRT	Acute	Dermatitis/ Skin reaction	1	66	20	20
Immunotherapy	Pembro + Cis wk	IMMUNO_CRT	Acute	Rash/ Acneiform	1	66	0	0
Immunotherapy	Nivo + Cis 3w	NIVO_CIS_3W	Acute	Dysphagia	1	618	21	21
Immunotherapy	Nivo + Cis 3w	NIVO_CIS_3W	Acute	Thyroid disorders	1	618	0	0
Immunotherapy	Nivo + Cis 3w	NIVO_CIS_3W	Acute	Treatment-related death	1	618		
Immunotherapy	Pembro + CRT	PEMBRO_CRT	Overall	Treatment-related death	1	398		
Other	Erythropoietin	EPO_RT	Overall	Treatment-related death	2	331		
Other	Xevinapant + CRT	XEVINAPANT_CRT	Hematologic	Anemia	1	364	21.4	21.4
Other	Xevinapant + CRT	XEVINAPANT_CRT	Infection	Pneumonia/ Infection	1	364	8	8
Platinum-based	Carbo + 5-FU	CARBO_5FU_RT	Acute	Mucositis/ Stomatitis	1	109	71	71
Platinum-based	Carbo + 5-FU	CARBO_5FU_RT	Acute	Feeding tube required	1	109	33	33
Platinum-	Carbo + 5-FU	CARBO_5FU	Acute	Weight loss	1	109	13	13

based		_RT						
Platinum-based	Carbo + 5-FU	CARBO_5FU_RT	Acute	Treatment-related death	1	109		
Platinum-based	Carbo + 5-FU	CARBO_5FU_RT	Late	Xerostomia/ Dry mouth	1	109	9	9
Platinum-based	Cisplatin 3w	CIS_3W	Acute	Neutropenia/ Leukopenia	3	555	42	24.1
Platinum-based	Cisplatin 3w	CIS_3W	Acute	Mucositis/ Stomatitis	4	596	41.5	39.6
Platinum-based	Cisplatin 3w	CIS_3W	Acute	Nephrotoxicity	1	62	0	0
Platinum-based	Cisplatin 3w	CIS_3W	Acute	Treatment-related death	1	95		
Platinum-based	Cisplatin 3w	CIS_3W	Hematologic	Anemia	1	356	14.3	14.3
Platinum-based	Cisplatin 3w	CIS_3W	Infection	Pneumonia/ Infection	1	356	3.1	3.1
Platinum-based	Cisplatin 3w	CIS_3W	Late	Hearing impairment	1	383	6.3	6.3
Platinum-based	Cisplatin 3w	CIS_3W	Overall	Treatment-related death	2	560		
Platinum-based	Cis + 5-FU	CIS_5FU_RT	Acute	Mucositis/ Stomatitis	1	130	38	38
Platinum-based	Cis + 5-FU	CIS_5FU_RT	Acute	Dermatitis/ Skin reaction	1	130	17	17
Platinum-based	Cisplatin wk	CIS_WK	Acute	Dysphagia	1	92	41	41
Platinum-based	Cisplatin wk	CIS_WK	Acute	Mucositis/ Stomatitis	4	528	41	46.2
Platinum-based	Cisplatin wk	CIS_WK	Acute	Severe acute toxicity	1	39	41	41
Platinum-based	Cisplatin wk	CIS_WK	Acute	Weight loss	1	92	21	21
Platinum-based	Cisplatin wk	CIS_WK	Acute	Dermatitis/ Skin reaction	2	157	17.5	16.6
Platinum-based	Cisplatin wk	CIS_WK	Acute	Fatigue	1	92	9	9
Platinum-based	Cisplatin wk	CIS_WK	Acute	Neutropenia/ Leukopenia	2	196	7.3	7.5
Platinum-based	Cisplatin wk	CIS_WK	Acute	Nausea/ Vomiting	1	92	4	4
Platinum-based	Cisplatin wk	CIS_WK	Acute	Rash/ Acneiform	1	92	1	1
Platinum-based	Cisplatin wk	CIS_WK	Acute	Treatment-related death	2	332		
Platinum-based	Cisplatin wk	CIS_WK	Late	Xerostomia/ Dry mouth	1	65	42	42
Platinum-based	Lobaplatin 3w	LOBAPLATIN_3W	Acute	Neutropenia/ Leukopenia	1	66	14	14
Platinum-based	Lobaplatin 3w	LOBAPLATIN_3W	Acute	Mucositis/ Stomatitis	1	66	9	9
RT alone	RT alone	RT_ALONE	Acute	Mucositis/ Stomatitis	7	653	26.6	26.6
RT alone	RT alone	RT_ALONE	Acute	Dysphagia	1	78	25.7	25.7
RT alone	RT alone	RT_ALONE	Acute	Severe acute toxicity	1	44	18	18
RT alone	RT alone	RT_ALONE	Acute	Dermatitis/ Skin reaction	2	197	16.5	12.5
RT alone	RT alone	RT_ALONE	Acute	Feeding tube required	1	113	13	13
RT alone	RT alone	RT_ALONE	Acute	Weight loss	1	113	5	5

RT alone	RT alone	RT_ALONE	Acute	Neutropenia/ Leukopenia	2	216	0.5	0.5
RT alone	RT alone	RT_ALONE	Acute	Treatment-related death	2	211		
RT alone	RT alone	RT_ALONE	Late	Xerostomia/ Dry mouth	2	170	14	11
RT alone	RT alone	RT_ALONE	Late	Perichondritis	1	52	0	0
RT alone	RT alone	RT_ALONE	Overall	Treatment-related death	2	320		

Supplementary Table S11. Treatment-related deaths. Per-arm mortality and aggregated rates by drug class.

Sheet: Per-Arm Deaths

Study	Arm	NMA Node	Treatment	Drug Class	N Safety	Death Rate (%)	Approx Deaths (n)
Mehanna_2019	Cetuximab + RT	CETUX_RT	Cetuximab	EGFR-targeted	165	2.4	4
Patil_2019	NCRT	NIMO_CIS_WK	Nimotuzumab	EGFR-targeted	267	4.5	12
Bourhis_2025_NIVOPOSTOP	Cisplatin + RT	NIVO_CIS_3W	Nivo + Cis 3w	Immunotherapy	306	1	3
Bourhis_2025_NIVOPOSTOP	Nivolumab + Cisplatin + RT	NIVO_CIS_3W	Nivo + Cis 3w	Immunotherapy	312	1	3
Machiels_2024	Pembrolizumab + CRT	PEMBRO_CRT	Pembro + CRT	Immunotherapy	398	1	4
Henke_2003	Epoetin <ce><b2> + RT	EPO_RT	Erythropoietin	Other	180	61	110
Hoskin_2009	Epoetin alfa + RT	EPO_RT	Erythropoietin	Other	151	49	74
Calais_1999	RT + Carboplatin/5-FU	CARBO_5FU_RT	Carbo + 5-FU	Platinum-based	109	1	1
Adelstein_2003	RT + Cisplatin q3w (Arm B)	CIS_3W	Cisplatin 3w	Platinum-based	95	4	4
Machiels_2024	Placebo + CRT	CIS_3W	Cisplatin 3w	Platinum-based	398	2	8
Mehanna_2019	Cisplatin 100mg/m2 + RT	CIS_3W	Cisplatin 3w	Platinum-based	162	1.2	2
Ghosh-Laskar_2016	Concurrent chemoradiotherapy (CTRT)	CIS_WK	Cisplatin wk	Platinum-based	65	3.1	2
Patil_2019	CRT	CIS_WK	Cisplatin wk	Platinum-based	267	2.6	7
Adelstein_2003	RT alone (Arm A)	RT_ALONE	RT alone	RT alone	98	2	2
Calais_1999	RT alone	RT_ALONE	RT alone	RT alone	113	0	0
Henke_2003	Placebo + RT	RT_ALONE	RT alone	RT alone	171	52	89
Hoskin_2009	Observation (RT alone)	RT_ALONE	RT alone	RT alone	149	50	74

Sheet: By Drug Class

Drug Class	N Arms	N Studies	Total Patients	Total Deaths	Weighted Death Rate (%)	Median Rate (%)	Range (%)
Other	2	2	331	184	55.6	55	49-61
RT alone	4	4	531	165	31.1	26	0-52
EGFR-targeted	2	2	432	16	3.7	3.5	2.4-4.5
Platinum-based	6	6	1096	24	2.2	2.3	1-4
Immunotherapy	3	2	1016	10	1	1	1-1

Supplementary Table S12. Meta-regression results. Effect of study-level covariates (oropharyngeal proportion, enrolment year) on treatment effects.

Sheet: Meta-Regression

Covariate	Coefficient	SE	95% CI Lower	95% CI Upper	p-value	N Studies	tau2	Interpretation
Oropharynx proportion (%)	0.006	0.0026	0.0009	0.0111	0.0207	26	0.0821	Significant effect modification
Larynx proportion (%)	-0.002	0.0042	-0.0102	0.0062	0.633	14	0.1011	No significant effect modification
Setting (definitive vs adjuvant/mixed)	0.0211	0.1208	-0.2156	0.2579	0.861	39	0.0627	No significant effect modification

Sheet: Subsite Composition

Study	Setting	T1	T2	HR (95% CI)	Oropharynx %	Larynx %	HPV+ %	Total N	Sensitivity Only
Gillison_2019	Definitive	Cetuximab	Cisplatin 3w	1.45 (1.03-2.05)	100	0	100	805	No
Mehanna_2019	Definitive	Cisplatin 3w	Cetuximab	5.00 (1.70-14.70)	100	0	94	334	No
Rishi_2013	Definitive	Cisplatin 3w	Con. boost RT	0.73 (0.34-1.18)	100	0		216	Yes
Gebre-Medhin_2025	Definitive	Cisplatin wk	Cetuximab	1.66 (1.08-2.56)	85.5		89	298	No
Rodriguez_2015	Definitive	Cisplatin 3w	Cis + 5-FU	5.30 (1.30-21.60)	82		71	69	Yes
Eriksen_2025	Definitive	Nimotuzumab	Zalu + Nimo + Cis	1.21 (0.91-1.61)	70		54.5	608	Yes
Bourhis_2012	Definitive	Very accel RT	Carbo + 5-FU	0.81 (0.67-0.99)	67			560	Yes
Martins_2013	Definitive	Cisplatin 3w	Erlotinib + Cis	1.05 (0.56-1.98)	67			204	Yes
Zandberg_2025	Definitive	Pembro + Cis wk	Seq Pembro + Cis	0.51 (0.19-1.37)	65.9		53	80	Yes
Geoffrois_2018	Definitive	Carbo + 5-FU	TPF + Cetux RT	1.12 (0.86-1.46)	64		24	360	Yes
Thomson_2024	Definitive	RT alone	Nimorazole	0.92 (0.65-1.31)	61.3	23.6	42	338	No
Tao_2022	Definitive	Cetuximab	Pembro + Cis wk	0.83 (0.49-1.40)	60.5		27.5	133	No
Nguyen-Tan_2014	Definitive	Cisplatin 3w	AFX + Cis + RT	0.96 (0.79-1.18)	59.75			743	Yes
Ghosh-Laskar_2016	Definitive	RT alone	Cisplatin wk	0.48 (0.24-0.97)	58	16		131	No
Mesia_2015	Definitive	Cisplatin 3w	Pani + Cis	1.63 (0.88-3.02)	52			153	Yes
Merlano_2020	Definitive	Cisplatin 3w	TPF + Cetux RT	1.05 (0.71-1.54)	52			242	Yes
Wozniak_2020	Adjuvant	RT alone	Cisplatin 3w	1.03 (0.57-1.87)	51.5			111	No
Machiels_2024	Definitive	Cisplatin 3w	Pembro + CRT	0.90 (0.71-1.15)	50.5		26.5	804	No

Patil_2019	Definitive	Cisplatin wk	Nimotuzumab	0.84 (0.65-1.08)	50.2	28.95	8.95	536	No
Giralt_2015	Definitive	Cisplatin 3w	Panitumumab	1.59 (0.91-2.79)	48	24	16	152	No
Gregoire_2011	Definitive	Cisplatin 3w	Gefitinib + Cis	1.06 (0.68-1.66)	48			226	Yes
Lee_1995	Definitive	RT alone	Etanidazole	0.95 (0.77-1.17)	47			504	No
Browman_1994	Definitive	RT alone	5-FU	0.73 (0.46-1.15)	42.3	26.9		175	No
Wendt_1998	Definitive	RT alone	Cis + 5-FU	0.35 (0.21-0.57)	42			298	No
Bourhis_2025_TrilynX	Definitive	Cisplatin 3w	Xevinapant + CRT	1.39 (1.04-1.86)	41.5	31.4	0	730	No
Henke_2003	Mixed	RT alone	Erythropoietin	1.39 (1.05-1.84)	41.5	23		351	No
Adelstein_2003	Definitive	RT alone	Cisplatin 3w	0.68 (0.48-0.96)	32.5			182	No
Calais_1999	Definitive	RT alone	Carbo + 5-FU	0.75 (0.56-1.00)	32			222	No
Gupta_2013	Definitive	Cis + 5-FU	Cis + Cape	1.67 (0.72-3.89)	32			138	Yes
Bernier_2004	Adjuvant	RT alone	Cisplatin 3w	0.70 (0.52-0.95)	30	22.5		334	No
Yi_2017	Definitive	RT alone	Cisplatin wk	0.74 (0.50-1.10)	29.65			222	No
Bourhis_2025_NIVOPOSTOP	Adjuvant	Cisplatin 3w	Nivo + Cis 3w	0.84 (0.62-1.15)	17.5	39.5	5	666	Yes
Henk_1977	Definitive	RT alone	Hyperbaric O2	0.69 (0.40-1.20)	9.7	46.6		103	No
Tao_2023	Definitive	Cisplatin 3w	Xevinapant + CRT	0.47 (0.27-0.84)	5		37.5	192	No
Noronha_2025	Adjuvant	Cisplatin wk	Cisplatin 3w	1.21 (0.90-1.62)	1.65			300	No
Forastiere_2013	Definitive	Cisplatin 3w	RT alone	1.08 (0.85-1.39)	0	100		547	No
Bonner_2010	Definitive	RT alone	Cetuximab	0.73 (0.56-0.95)	0			424	No
Dietz_2018	Definitive (larynx preservation)	Post-ind RT	Post-ind Cetux	1.04 (0.60-1.78)	0	100		173	Yes
Fietkau_2020	Definitive	Paclitaxel + Cis	Cis + 5-FU	0.82 (0.54-1.24)	0	9.25		216	No
Zhang_2023	Definitive	Cisplatin 3w	Lobaplatin 3w	0.88 (0.40-1.94)	0	33.5		128	No
Janssens_2012	Definitive	Accelerated RT	ARCON + Accel RT	1.03 (0.73-1.46)	0	100		345	Yes
Cooper_2012	Adjuvant	RT alone	Cisplatin 3w	0.89 (0.70-1.12)				410	No
Caudell_2023	Definitive	Cisplatin 3w	Cis + Cetuximab	0.97 (0.80-1.16)				891	No
Hitt_2022	Definitive	Cisplatin 3w	Cetuximab	1.11 (0.85-1.44)					No
Hoskin_2009	Mixed	RT alone	Erythropoietin	1.04 (0.75-1.43)				300	No
Eschwege_1997	Definitive	RT alone	Etanidazole	1.05 (0.68-1.61)					No
Fazekas_1987	Definitive	RT alone	Misonidazole	1.27 (0.71-2.27)					No
Kiyota_2022	Adjuvant	Cisplatin 3w	Cisplatin wk	0.75 (0.50-1.13)					No

Lee_2021	Definitive	Cisplatin 3w	Avelumab + Cis	1.31 (0.93-1.85)					No
Ghadjar_2012	Definitive	RT alone	Cisplatin daily	0.77 (0.56-1.11)					No
Patil_2023	Mixed	RT alone	Docetaxel wk	0.75 (0.57-0.98)					No
Bachaud_1996	Adjuvant	RT alone	Cisplatin wk	0.26 (0.10-0.71)				88	No
Maddalo_2020	Definitive	Cisplatin wk	Cetuximab	1.41 (0.55-3.59)					No
Heukelom_2016	Definitive	Cisplatin 3w	IA Cisplatin	0.85 (0.62-1.17)				237	No
Bourhis_2011	Definitive	Very accel RT	Cis + 5-FU	0.95 (0.63-1.43)				109	Yes
Gupta_2015	Definitive	Accelerated RT	Cisplatin wk	1.02 (0.60-1.74)				133	Yes
Mell_2024	Definitive	Cetuximab	Durvalumab	1.30 (0.74-2.28)				186	Yes
Janoray_2020	Definitive	Cisplatin 3w	Cetuximab	1.04 (0.55-1.98)				116	Yes
Harrington_2013	Definitive	Cisplatin 3w	Lapatinib + Cis	0.90 (0.44-1.84)				67	Yes
Hassan_Metwally_2015	Definitive	Accelerated RT	Nimo + Accel RT	0.65 (0.35-1.19)				82	Yes
Borel_2024	Mixed	Cisplatin 3w	Cis frac 3w	0.86 (0.50-1.47)				122	Yes

Supplementary Table S13. Era subgroup analysis. Meta-regression by enrolment era with era-stratified P-scores and study classification.

Sheet: Meta-Regression

Covariate	Coefficient	SE	95% CI Lower	95% CI Upper	p-value	N Studies	tau2	R-squared (%)	Interpretation
Enrollment start year (continuous)	0.00918	0.00425	0.00085	0.0175	0.0307	39	0.0513	12.2	Significant effect modification
Era (post-2000 vs pre-2000)	0.18365	0.10004	-0.01244	0.37973	0.0664	39	0.0549	6.1	No significant effect modification
Era (post-2005 vs pre-2005)	0.24326	0.09607	0.05498	0.43155	0.0113	39	0.0495	15.2	Significant effect modification

Sheet: P-scores Cutoff 2000

Treatment	P-score Pre (%)	Rank Pre	P-score Post (%)	Rank Post	|Rank Diff|	|P-score Diff|
Cisplatin wk	95.3	1	74	0.7	0.3	21.3
Cis + 5-FU	93.1	0.9				
Hyperbaric O2	62.1	0.6				
Cetuximab	61.2	0.6	33.4	0.3	0.3	27.8
Carbo + 5-FU	58.7	0.6				
5-FU	58.5	0.6				
Cisplatin daily	54.7	0.5				
Cisplatin 3w	48.6	0.5	46.2	0.5	0	2.39999999999999 9
Etanidazole	27.1	0.3				
RT alone	22.3	0.2	36.3	0.4	0.2	14
Misonidazole	11.6	0.1				
Erythropoietin	6.6	0.1				
Avelumab + Cis			28.2	0.3		
Cis + Cetuximab			49.5	0.5		
Docetaxel wk			60.4	0.6		
IA Cisplatin			59.4	0.6		
Pembro + Cis wk			50.9	0.5		
Lobaplatin 3w			55.7	0.6		
Nimorazole			45.5	0.5		
Nimotuzumab			79.3	0.8		
Panitumumab			18.9	0.2		
Pembro + CRT			55.3	0.6		
Xevinapant + CRT			56.9	0.6		

Sheet: P-scores Cutoff 2005

Treatment	P-score Pre (%)	Rank Pre	P-score Post (%)	Rank Post	|Rank Diff|	|P-score Diff|
Cis + 5-FU	98.5	1				
Cisplatin wk	79.8	0.8	67.4	0.7	0.10000000000000001	12.39999999999999
IA Cisplatin	65.2	0.7				
Hyperbaric O2	62.8	0.6				
Cetuximab	60	0.6	31.9	0.3	0.3	28.1
5-FU	58.4	0.6				
Carbo + 5-FU	57.5	0.6				
Cisplatin daily	53.7	0.5				
Cisplatin 3w	47.6	0.5	46.1	0.5	0	1.5
Etanidazole	25.9	0.3				
RT alone	21.6	0.2	47.9	0.5	0.3	26.3
Misonidazole	12	0.1				
Erythropoietin	7.2	0.1				
Avelumab + Cis			29.7	0.3		
Cis + Cetuximab			49.1	0.5		
Docetaxel wk			65.7	0.7		
Pembro + Cis wk			48.5	0.5		
Lobaplatin 3w			54.9	0.5		
Nimorazole			53.5	0.5		
Nimotuzumab			73.6	0.7		
Panitumumab			20.7	0.2		
Pembro + CRT			54.3	0.5		
Xevinapant + CRT			56.9	0.6		

Sheet: Study Era Classification

Study	Enrollment Start	Setting	Era (2000)	Era (2005)
Henk_1977	1971	Definitive	Pre-2000	Pre-2005
Fazekas_1987	1979	Definitive	Pre-2000	Pre-2005
Bachaud_1996	1984	Adjuvant	Pre-2000	Pre-2005
Browman_1994	1987	Definitive	Pre-2000	Pre-2005
Eschwege_1997	1987	Definitive	Pre-2000	Pre-2005
Lee_1995	1988	Definitive	Pre-2000	Pre-2005
Wendt_1998	1989	Definitive	Pre-2000	Pre-2005
Forastiere_2013	1992	Definitive	Pre-2000	Pre-2005
Adelstein_2003	1992	Definitive	Pre-2000	Pre-2005
Ghadjar_2012	1994	Definitive	Pre-2000	Pre-2005
Bernier_2004	1994	Adjuvant	Pre-2000	Pre-2005
Calais_1999	1994	Definitive	Pre-2000	Pre-2005
Cooper_2012	1995	Adjuvant	Pre-2000	Pre-2005
Henke_2003	1997	Mixed	Pre-2000	Pre-2005
Bonner_2010	1999	Definitive	Pre-2000	Pre-2005

Hoskin_2009	1999	Mixed	Pre-2000	Pre-2005
Heukelom_2016	2000	Definitive	Post-2000	Pre-2005
Yi_2017	2002	Definitive	Post-2000	Pre-2005
Ghosh-Laskar_2016	2002	Definitive	Post-2000	Pre-2005
Caudell_2023	2005	Definitive	Post-2000	Post-2005
Wozniak_2020	2007	Adjuvant	Post-2000	Post-2005
Giralt_2015	2007	Definitive	Post-2000	Post-2005
Hitt_2022	2008	Definitive	Post-2000	Post-2005
Gillison_2019	2011	Definitive	Post-2000	Post-2005
Fietkau_2020	2011	Definitive	Post-2000	Post-2005
Maddalo_2020	2011	Definitive	Post-2000	Post-2005
Mehanna_2019	2012	Definitive	Post-2000	Post-2005
Patil_2019	2012	Definitive	Post-2000	Post-2005
Kiyota_2022	2012	Adjuvant	Post-2000	Post-2005
Noronha_2025	2013	Adjuvant	Post-2000	Post-2005
Gebre-Medhin_2025	2013	Definitive	Post-2000	Post-2005
Thomson_2024	2014	Definitive	Post-2000	Post-2005
Tao_2022	2016	Definitive	Post-2000	Post-2005
Tao_2023	2016	Definitive	Post-2000	Post-2005
Zhang_2023	2016	Definitive	Post-2000	Post-2005
Lee_2021	2016	Definitive	Post-2000	Post-2005
Machiels_2024	2017	Definitive	Post-2000	Post-2005
Patil_2023	2017	Mixed	Post-2000	Post-2005
Bourhis_2025_TrilynX	2020	Definitive	Post-2000	Post-2005
